# Melatonin Modulates Glucose Metabolism Reprogramming via Targeting G6PD to Alleviate Lead‐Induced Hepatocytes Pyroptosis in Common Carp (*Cyprinus carpio* L.)

**DOI:** 10.1002/advs.202501041

**Published:** 2025-08-11

**Authors:** Zhiying Miao, Xiaofeng Ji, Lai Wei, Zhiruo Miao, Shiwen Xu

**Affiliations:** ^1^ College of Life Science Northeast Agricultural University Harbin 150030 China; ^2^ Harbin Hualong Feed Development Co., Ltd. No.1301 Jichang Street Harbin, Asia 150077 China; ^3^ College of Animal Science and Technology Northeast Agricultural University Harbin 150030 China; ^4^ College of Veterinary Medicine Northeast Agricultural University Harbin 150030 China

**Keywords:** glucose metabolism reprogramming, glucose‐6‐phosphate dehydrogenase, histone H3 lysine 18 lactylation, lead, melatonin

## Abstract

Lead (Pb) is a prevalent toxic contaminant that accumulates in freshwater ecosystems, posing severe toxicity to non‐target species such as fish and contributing to the pathogenesis of liver disease. Melatonin (Mel) is a well‐known natural antioxidant that has been found to improve liver function through its potent anti‐inflammatory properties. However, whether and how Mel alleviates Pb‐triggered hepatotoxicity remains unclear. Mitochondria play a vital role in glucose metabolism, and glucose metabolic reprogramming is characterized by elevated glycolysis, resulting in lactate accumulation, which is a precursor for histone lactylation, an epigenetic modification. In this study, it is demonstrated that Pb triggers glucose metabolism reprogramming, resulting in lactate accumulation. Specifically, lactate links glycolysis and mitochondrial homeostasis via histone H3 lysine 18 lactylation (H3K18la), which modulates the activity of dynamin‐related protein 1 (DRP1). Furthermore, DRP1 actively mediates mitochondrial fragmentation, thereby facilitating inflammatory signals derived from the cyclic GMP‐AMP synthase (cGAS)‐stimulator of interferon genes (STING) pathway. Additionally, the results first demonstrate that Mel redirects glucose carbon utilization from glycolysis to the pentose phosphate pathway (PPP) by targeting glucose‐6‐phosphate dehydrogenase (G6PD). In summary, Mel targets G6PD to suppress glycolysis‐driven H3K18la and DRP1 transcription, thereby maintaining mitochondrial homeostasis to alleviate hepatocytes pyroptosis dependent on cGAS‐STING pathway under Pb exposure.

## Graphical abstract

This study reveals that Lead reprograms glucose metabolism to enhance H3K18la, driving DRP1‐dependent mitochondrial DNA leakage and cGAS‐STING‐mediated pyroptosis. Melatonin targets G6PD to suppress glycolysis‐driven H3K18la and restore mitochondrial homeostasis, thereby alleviating Lead‐induced liver injury in common carp through disruption of the mtDNA‐cGAS‐STING axis. The results present a metabolic‐epigenetic crosstalk underlying Lead hepatotoxicity and the therapeutic potential of Melatonin.

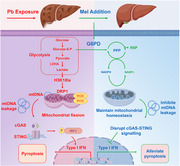

## 1 Introduction

Lead (Pb) is a persistent and non‐essential heavy metal, has emerged as a critical environmental contaminant due to its pervasive presence in aquatic ecosystems. Recognized by the United States Environmental Protection Agency (USEPA) as one of 275 priority‐controlled contaminants by the United States Environmental Protection Agency (USEPA),^[^ ^1^ ^]^ Pb contamination remains a global concern. Recent monitoring data reveal alarming contamination levels in Chinese freshwater systems, with Pb concentrations reaching 4.13 mg kg^−1^ in fish specimens from major river basins‐exceeding both the USEPA recommended threshold of 0.3 mg kg^−1^ and the European Union (EU) limit of 0.2 mg kg^−1^ by over 20‐fold.^[^ ^2^ ^]^ As similarly reported in the study of Izmir Bay, the Pb content in the central and peripheral sediments of the bay ranged from 9.8 to 119 µg g^−1^ and from 3.1 to 94 µg g^−1^, respectively, exceeding the background value. Additionally, as the main fish species in the Izmir Bay area, *Mullus barbatus* and *Solea vulgaris* had the highest Pb content in the body, reaching 478 and 491 µg kg^−1^, respectively.^[^ ^3^ ^]^ Previous evidence has revealed that the liver is particularly susceptible to Pb accumulation compared to other tissues in fish, including the kidney, gill, and gut.^[^ ^4^ ^]^ While it is known that Pb can be damaging to the liver, causing hydropic degeneration, capsular fibrosis, and hepatocyte necrosis in rodent models,^[^ ^5^ ^]^ this particular aspect of Pb toxicity has not received sufficient research focus or attention. Pb exposure disrupts glucose metabolism by impairing glycogenolysis and gluconeogenic enzyme activity,^[^ ^6^, ^7^ ^]^ with documented hepatotoxic effects in Institute of Cancer Research (ICR) mice and Japanese quails (*Coturnix japonica*).^[^ ^8^, ^9^ ^]^ Recent studies reveal that Pb triggers hepatocyte pyroptosis in murine models,^[^ ^10^ ^]^ a process implicated in non‐alcoholic fatty liver disease (NAFLD) pathogenesis,^[^ ^11^ ^]^ suggesting that inhibiting pyroptosis could hold potential for the development of therapeutic strategies for liver disorders. Pyroptosis is mediated by endoplasmic reticulum (ER) stress‐mediated activation of the unfolded protein response (UPR), GSDMD‐dependent pore formation, and crosstalk between lipid peroxidation and ferroptosis. However, the mechanisms driving Pb‐induced pyroptosis in *Cyprinus carpio* hepatocytes remain elusive.

As the rate‐limiting enzyme in the pentose phosphate pathway (PPP), glucose‐6‐phosphate dehydrogenase (G6PD) provides a glucose‐oxidizing pathway, consuming glucose‐6‐phosphate to produce nicotinamide adenine dinucleotide phosphate (NADPH), which is required to sustain intracellular redox homeostasis.^[^ ^13^ ^]^ Accumulating evidence suggests that an increased dependence on the glucose metabolism reprogramming for inflammatory damage.^[^ ^14^, ^15^, ^16^ ^]^ Glycolysis utilizes the glucose converted to pyruvate, which enters the mitochondria to participate in the ATP synthesis process via tricarboxylic acid (TCA) cycle and oxidative phosphorylation (OXPHOS). Lactate as the primary metabolic product of glycolysis, which aggregates and amplifies inflammatory signaling to cause tissue damage.^[^ ^11^, ^17^ ^]^ Lactylation, a newly identified posttranslational modification, mediates protein functional regulation through lactyl group conjugation utilizing glycolysis‐derived lactate.^[^ ^18^ ^]^ Meanwhile, the glucose metabolic perturbations‐enhanced mitochondrial OXPHOS and consequent increase in reactive oxygen species (ROS) production are considered the emerging hallmarks of mitochondrial fragmentation and dysfunction.^[^ ^19^ ^]^ The resulting mitochondrial dysfunction feedback exacerbates the overgeneration of ROS and further intensifies mitochondrial fragmentation. A growing number of findings highlight that mitochondrial DNA (mtDNA) is more susceptible to oxidative stress than nuclear DNA,^[^ ^20^ ^]^ and the leakage of mtDNA is recognized as a crucial trigger of inflammatory response, particularly the cyclic GMP‐AMP synthase (cGAS)‐stimulator of those interferon genes (STING) signaling pathway activation.^[^ ^21^, ^22^ ^]^ A recent study further demonstrated that the effects of STING‐regulated network not only induces type‐I interferon (IFN) responses to amplify inflammatory cytokine release, but also participate in nucleotide‐binding oligomerization domain, leucine‐rich repeat and pyrin domain‐containing 3 (NLRP3) inflammasome activation.^[^ ^23^ ^]^

Melatonin (N‐acetyl‐5‐methoxytryptamine; Mel), a naturally occurring indoleamine with eco‐physiological compatibility, orchestrates diverse physiological functions across multiple organ systems.^[^ ^24^, ^25^, ^26^ ^]^ Notably, the programming function of Mel on glucose metabolism has been reported and achieved by facilitating PPP.^[^ ^27^ ^]^ However, critical questions remain unresolved: Does Pb impacts lactylation modifications by modulating glycolytic reprogramming? Does it further exacerbate pyroptosis and thereby worsen hepatic inflammatory damage? Can Mel ameliorate Pb‐impaired glucose metabolism and subsequently attenuate inflammatory responses? Accumulating evidence has established a mechanistic link between glycolytic reprogramming and programmed cell death modalities, particularly pyroptosis.^[^ ^28^, ^29^, ^30^ ^]^ Although lactylation, a recently identified post‐translational modification, is intrinsically linked to glucose metabolism, its functional crosstalk with pyroptosis and the underlying regulatory mechanisms remain poorly characterized.

Given the global prevalence of Pb contamination, this study established Pb exposure or/and Mel supplement models in carp liver and L8824 cells to address three objectives: Elucidate the regulatory role of Mel in Pb‐driven glucose metabolic reprogramming; Dissect the mechanism by which Mel restores mitochondrial dynamic homeostasis under Pb‐triggered oxidative stress; Assess inhibitory effect of Mel on Pb‐mediated hepatocyte pyroptosis and its anti‐inflammatory potential against Pb‐induced hepatic injury. Through integrated multi‐omics approaches and functional validation experiments, we systematically investigated the tripartite interplay among Pb‐induced glycolytic reprogramming, mitochondrial dyshomeostasis, and pyroptosis, while delineating counteractive mechanisms of Mel. Our findings unveil novel detoxification mechanisms of Mel against Pb‐induced hepatotoxicity, including the first demonstration of its antioxidative action by binding with G6PD, thereby establishing a pivotal role of Mel in mitigating Pb‐induced liver injury in *Cyprinus carpio*.

## 2 Results

### 2.1 Geographical Distribution of Pb Contamination in the World

**Figure 1:**
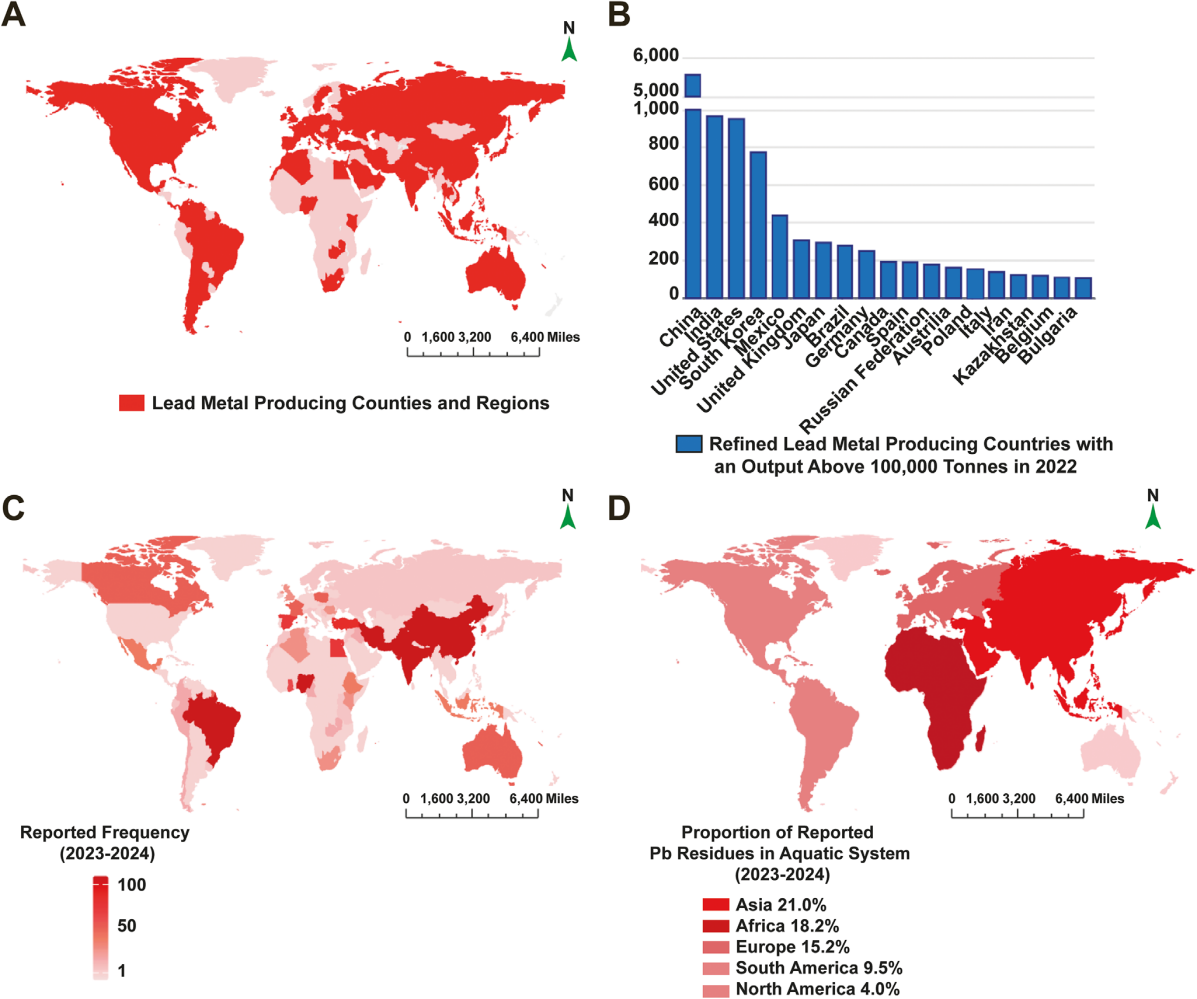
Geographical distribution map of Pb residues. A) Pb metal producing countries and regions worldwide in 2022. B) Refined Pb metal producing countries with an output over 100 000 tonnes in 2022. C) Frequency of countries and regions in 330 Pb environmental residue reports in 2023–2024. D) Reported frequency of Pb residue in freshwater system in 2023–2024.

As depicted in the geographical map of Pb (**Figure** **1**A,B), global Pb production involves over 80 countries engaged in Pb smelting and refining activities, with China, India, the United States, South Korea, and Mexico ranking as the top five refined Pb producers.

Preliminary screening of Pb residue data (Figure 1C) identified China, India, Pakistan, the United States, Brazil, Iran, Bangladesh, Nigeria, Turkey, and Egypt as the top ten countries with the highest reported frequencies of Pb residues, underscoring the global pervasiveness of Pb contamination. Our analysis of Pb residues in aquatic systems (Figure 1D) revealed that Asia accounts for the highest regional proportion (21.0%) of reported cases, while toxicological investigations in other regions remain critically understudied.

### 2.2 Mel Improves Pb‐Induced Hepatitis Injury by Alleviating Hepatocyte Pyroptosis

**Figure 2:**
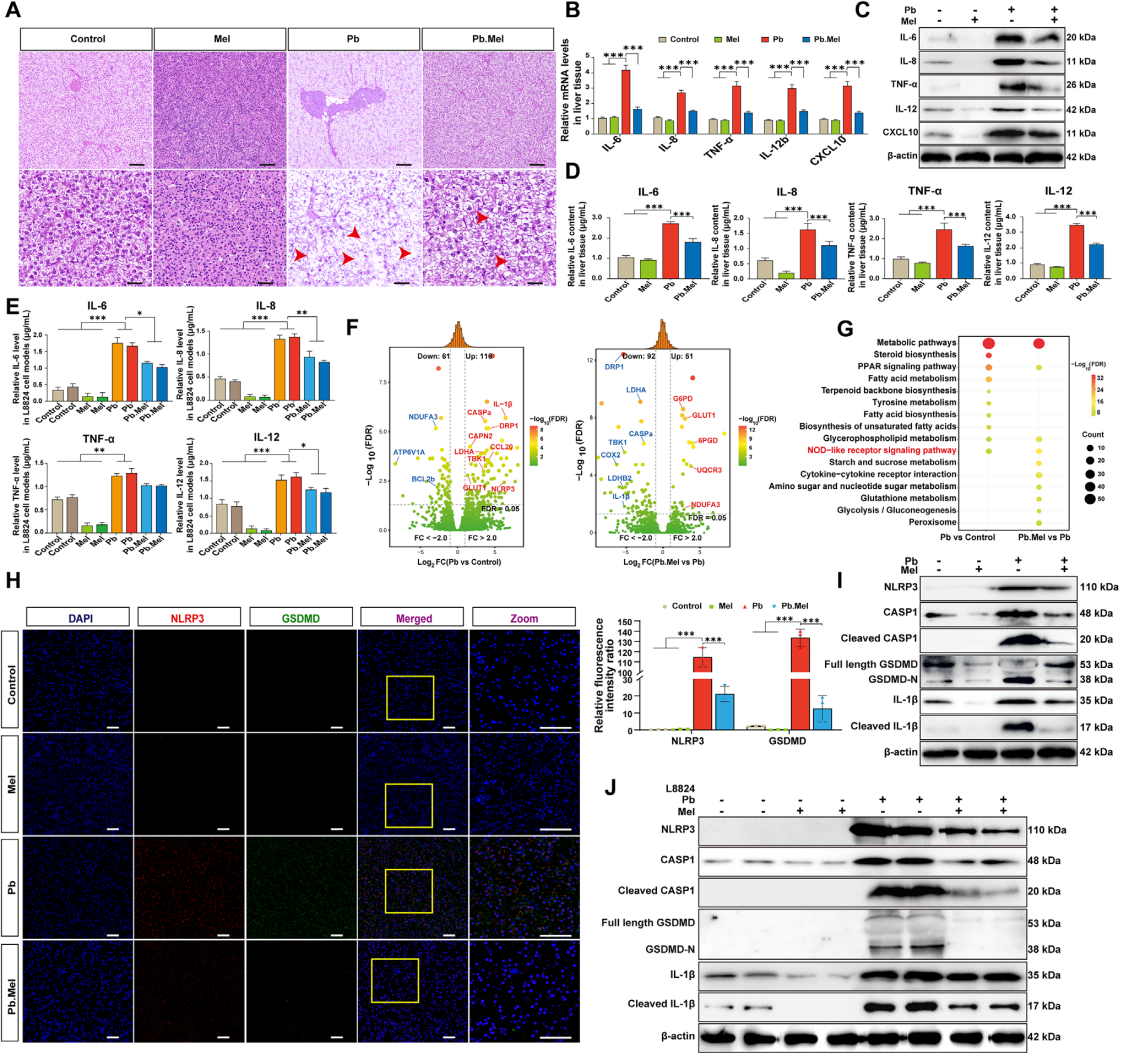
Mel improves Pb‐induced hepatitis injury by relieving hepatocyte pyroptosis. A) H&E staining of liver tissue with Pb and/or Mel treatment. B) The mRNA expression of marker inflammatory genes in liver tissues. Each value represents the mean ± SD, ^*^ *p* < 0.05, ^**^ *p* < 0.01, and ^***^ *p* < 0.001 by one‐way ANOVA with Tukey's multiple comparison test. C) The protein expression of marker inflammatory genes in liver tissues. D) The content of marker inflammatory genes in liver tissues determined by ELISA kits (*n* = 6) by one‐way ANOVA. E) The marker inflammatory cytokine content in the L8824 cell models (*n* = 6) by one‐way ANOVA. F) The volcano plot of profile gene expressions (log_2_|Fold change| > 1 and adjusted *p*‐values < 0.05 (FDR < 0.05)). G) The enriched KEGG pathways between Pb and Control, and between Pb.Mel and Pb. H) The dual‐immunofluorescent staining of liver tissues with antibody of NLRP3 (Red), GSDMD (Green) and DAPI (Blue) (Scale bar = 50 µm). I) The protein level of pyroptosis‐related genes in liver tissue. J) The protein level of pyroptosis‐related genes in vitro.

To assess the inflammatory alterations of liver tissue upon Pb exposure, the hematoxylin and eosin (H&E) staining on the groups is illustrated in **Figure** **2**A, Pb exposure induced pronounced histopathological alterations, including hepatocyte vacuolization, cytoplasmic hyalinization, and necrosis (red arrowheads), whereas Mel cotreatment substantially alleviated these pathological features. Then, the expression of marker inflammatory genes, including IL‐6, IL‐8, TNF‐α, IL‐12, and CXCL10 were significantly (*p <* 0.001) up‐regulated in the Pb group, while it was significantly (*p <* 0.001) down‐regulated (*p <* 0.001) in the Pb.Mel group both at mRNA and protein levels (Figure 2B,C; Figure S1A, Supporting Information). To further validate these findings, we established the L8824 hepatocyte model. Quantification of inflammatory cytokines (Figure S2B, Supporting Information) demonstrated that Pb exposure significantly elevated cytokine levels (*p <* 0.001), which were attenuated by Mel treatment (*p <* 0.05) in a protein‐dependent manner. Furthermore, the content of IL‐6, TNF‐α, IL‐8, and IL‐12 was detected with ELISA kits both in vivo and in vitro (Figure 2D,E), demonstrating that Pb exposure significantly (*p <* 0.001) enhanced inflammatory cytokines production and exhibited a notable (*p <* 0.05) remission with Mel addition.

The transcriptome sequence analysis was constructed to profile gene expressions in hepatocytes of common carps treated with Pb and/or Mel. Differentially expressed genes (DEGs) were identified using thresholds of log_2_|Fold change| > 1 and adjusted *p <* 0.05 (FDR < 0.05). Specifically, 116 genes were significantly up‐regulated and 61 genes were down‐regulated with Pb exposure, while 51 genes were significantly up‐regulated and 93 genes were down‐regulated after Mel addition as compared to the Pb group (Figure 2F). Subsequent gene expression profiling and Gene Ontology (GO) enrichment analysis were conducted as depicted in Figure S2A (Supporting Information). As expected, the Kyoto Encyclopedia of Genes and Genomes (KEGG) enrichment analysis revealed Pb exposure activated multiple inflammatory pathways, particularly the NOD‐like receptor signaling pathway (Figure 2G; Figure S2B, Supporting Information). This finding was corroborated by pathway analysis of inflammation‐related DEGs (Figure S2C, Supporting Information), suggesting the involvement of pyroptosis in Pb‐mediated hepatic inflammatory injury. Subsequently, the expression of pyroptosis‐related genes was further detected. The dual‐immunofluorescence staining of anti‐NLRP3 and anti‐GSDMD (Figure 2H) manifested that the expression of NLRP3 and GSDMD expression was significantly (*p <* 0.001) enhanced with Pb exposure, which was effectively reversed by Mel treatment. Consistently, protein levels of pyroptosis‐related genes, including NLRP3, Cleaved CASP1, GSDMD‐N, and Cleaved IL‐1β, showed significant (*p <* 0.05) elevation in both liver tissues and L8824 cell models under Pb exposure (Figure 2I,J; Figure S4, Supporting Information), elucidating that Pb exposure‐mediated pyroptosis contributes to haptic inflammatory injury. Although Figure S2A (Supporting Information) initially highlights Pb‐induced transcriptional changes (Pb vs Control), subsequent pathway analyses (Figure 2G; Figure S2B,C, Supporting Information) and experimental validation of pyroptosis markers (Figure 2H–J; Figure S3, Supporting Information) collectively demonstrate that Mel mitigates Pb‐mediated pyroptosis.

### 2.3 Mel Alleviates Pb‐Induced Lactate Accumulation by Targeting G6PD

**Figure 3:**
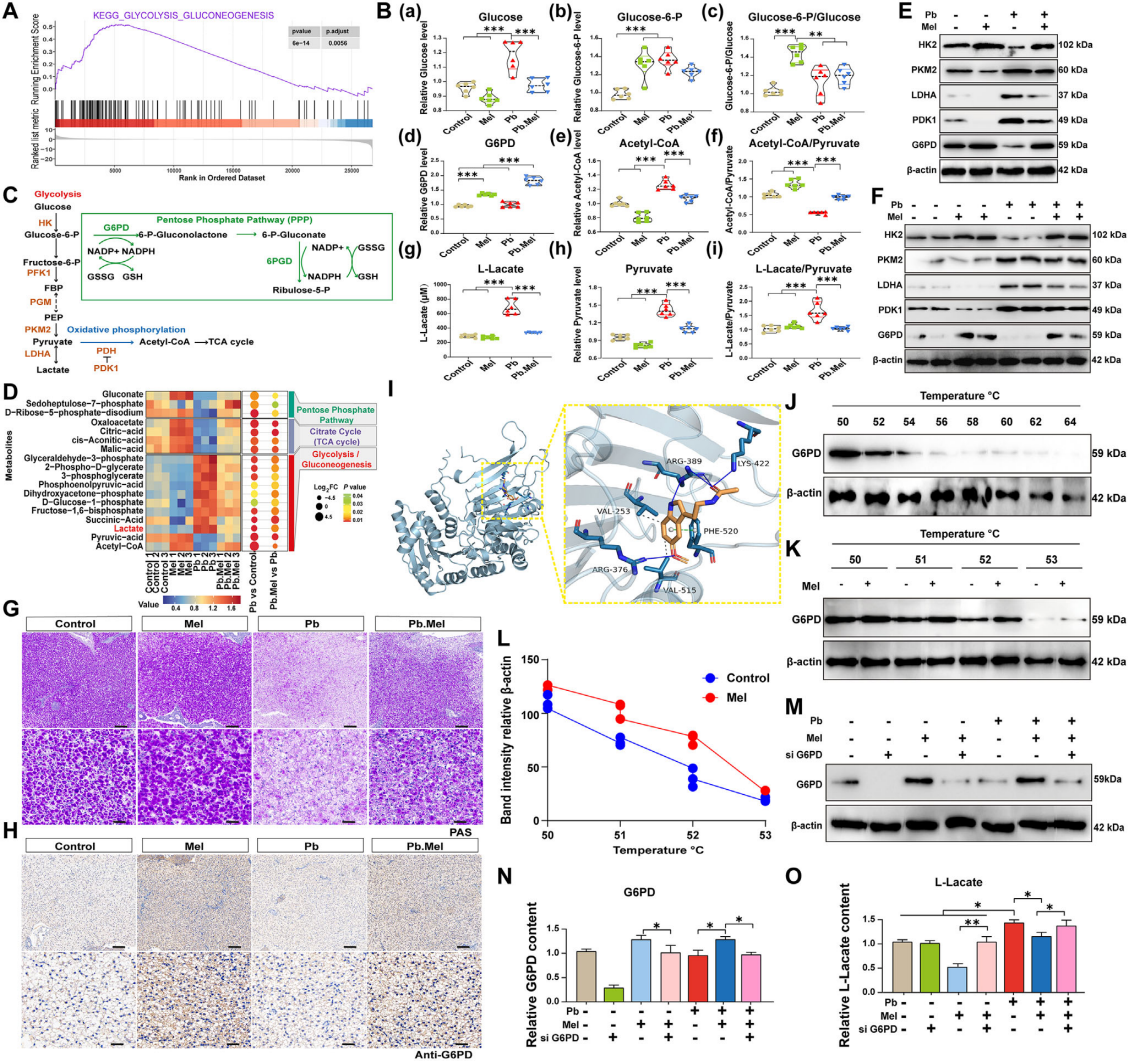
Mel targets G6PD to alleviate Pb‐induced lactate accumulation. A) The results of GSEA enrichment in the transcriptomics of carp liver. B) The content of crucial glucose metabolism‐related metabolites and enzymes (*n* = 6) by one‐way ANOVA. C) The related pathway of glucose metabolism. D) The enrichment of metabolites was analyzed by targeted metabolomics. E) The expression of glycolysis‐related genes in carp liver. F) Protein levels of glycolytic‐related genes in vitro. G) PAS staining of carp liver (Scale bar = 50 µm/20 µm in upper/lower panel). H) IHC of anti‐G6PD staining (Scale bar = 50 µm/20 µm in upper/lower panel). I) Molecular docking prediction of Mel targeting G6PD. J) CETSA results G6PD and Mel. K) CETSA results of G6PD in vitro. L) Statistic results of CETSA. M) Protein level of G6PD in the si G6PD models with Pb and/or Mel treatment. N) The content of G6PD in vitro models (*n* = 6) by one‐way ANOVA with Tukey's multiple comparison test. O) Lactate content in vitro (*n* = 6) by one‐way ANOVA.

Gene set enrichment analysis (GSEA) of carp liver transcriptomes revealed significant enrichment of glycolysis pathways in the Pb‐exposed group compared to the control (**Figure** **3**A). Subsequent metabolic profiling of liver tissues showed that Pb exposure significantly altered carbon metabolite levels, including a decreased Glucose‐6‐P/Glucose ratio (hexokinase; Figure 3Ba–c), a reduced Acetyl‐CoA/Pyruvate ratio (Figure 3Bd–f), and an elevated Lactate/Pyruvate ratio (lactate dehydrogenase (LDH); Figure 3Bg–i). These metabolic shifts corresponded with increased glycolytic flux, elevated LDH activity, and lactate accumulation in the Pb group. Concurrent downregulation of OXPHOS‐related genes reveals glucose metabolic reprogramming toward glycolysis under Pb exposure (Figure S4A, Supporting Information). Notably, Mel treatment enhanced PPP activity, as evidenced by elevated ATP, NADPH, and GSH levels (Figure 3C; Figure S4B, Supporting Information).

To validate whether the exposure of Pb and Mel affects carbon metabolite levels, the targeted metabolomic analysis were performed as Figure 3D and Figure S5 (Supporting Information), elucidating that Mel supplementation counteracted Pb‐induced glycolytic activation by enhancing PPP flux. Consistently, the expression of glycolysis‐related genes was assessed and displayed the level of HK2 and G6PD were down‐regulated with Pb exposure and elevated with Mel treatment, while PKM2, LDHA, and PDK1 were conversely up‐regulated under Pb exposure (Figure 3E,F). Histological Periodic acid‐Schiff (PAS) staining and G6PD immunohistochemistry (IHC) further supported that G6PD serves as a critical regulator in the diminished role of Mel Pb‐mediated glycolytic profile (Figure 3G,H). Whereafter, molecular docking predicted the targeting interaction between Mel and G6PD (Figure 3l) with a docking score of −6.2 kcal mol^−1^. The rightward shift of the G6PD melting curve upon Mel treatment indicates a direct binding between Mel and G6PD, which was subsequently validated by cellular thermal shift assay (CETSA) (Figure 3J,K). Importantly, G6PD knockdown significantly (*p* < 0.05) abolished the effects of Mel on G6PD elevation (Figure 3L–O), elucidating that G6PD acts as a key molecular mediator in the modulation of Mel on glucose metabolism.

### 2.4 Mel Mitigates H3K18 Lactylation by Targeting G6PD to Improve Pb‐Mediated Mitochondrial Oxidative Stress

**Figure 4:**
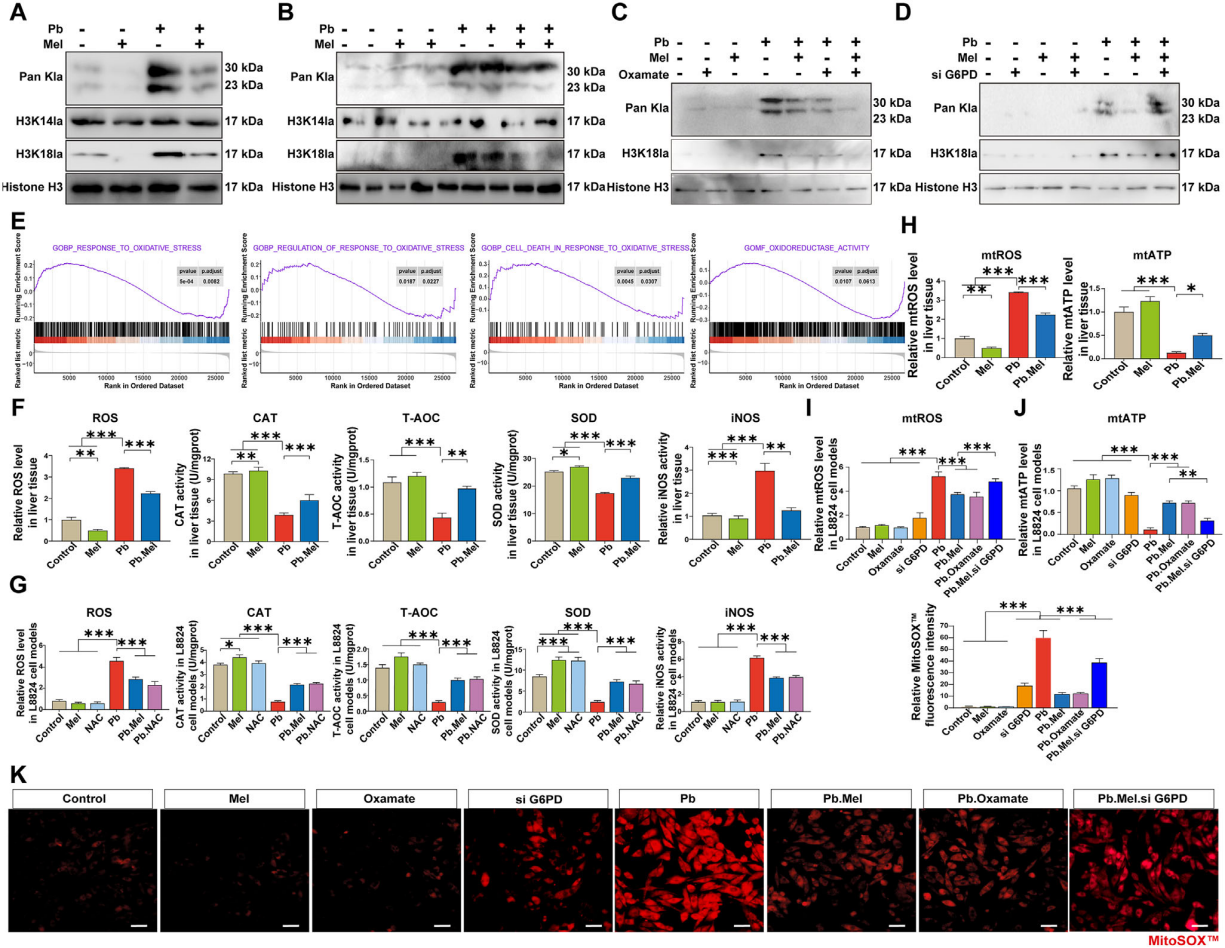
Mel improves Pb‐mediated mitochondrial oxidative stress by alleviating H3K18 lactylation. A) The lactylation modification of carp liver. B) The lactylation modification in vitro. C) The alter of lactylation modification in vitro models using Oxalate as glycolysis inhibitor. D) The lactylation modification changes in si G6PD models. E) Oxidative stress‐related enrichment of GSEA in the liver transcriptome of carp. F) The content of ROS and activities of the CAT, T‐AOC, SOD, iNOS in liver tissues (*n* = 6) by one‐way ANOVA with Tukey's multiple comparison test. G) the content of oxidative stress indicators (*n* = 6). I) Relative mtROS levels in cell models (*n* = 6). J) Relative mtATP levels in vitro (*n* = 6). K) MitoSOX Red staning of cell models (Scale bar = 20 µm).

Given that elevated lactate levels were observed following Pb exposure, we sought to investigate whether histone post‐translational modification contributed to hepatoxicity. Our results demonstrated that both pan‐lysine lactylation (Pan kla) and histone H3K18 lactylation (H3K18la) were increased in the Pb group as compared to both the control and Mel groups (**Figure** **4**A,B). To confirm the involvement of lactate metabolism, we employed Oxamate, a specific LDHA inhibitor, to find out that Mel exhibited alleviated effects on Pb‐induced H3K18la, which is consistent with the addition of Oxamate (Figure 4C). However, this protective effect was abolished in G6PD‐knockdown cell models (Figure 4D), demonstrating that Pb facilities H3K18la via glycolytic lactate accumulation, while Mel ameliorates lactylation by targeting G6PD. Consistent with previous findings, GSEA analysis indicated significant enrichment of the oxidative stress pathway in Pb‐exposed groups (Figure 4E). Subsequent assays demonstrated that Pb exposure markedly reduced CAT, T‐AOC, and SOD activities (*p <* 0.001), concomitantly elevating ROS levels and iNOS activity both in vivo and in vitro. Importantly, these perturbations were effectively rescued by Mel and NAC treatments in cellular models (Figure 4F,G). Then, the mitochondrial ROS (mtROS) was assessed and shown to be significantly (*p <* 0.001) elevated with Pb exposure, while the mitochondrial ATP (mtATP) levels were found to decrease (*p <* 0.001) in the Pb group, both of which were restored with Mel addition (Figure 4H,I). Consistently, the intensified mtROS content and eliminated mtATP level were observed in the Pb group, and remitted by both Mel and Oxamate addition, whereas the mitigated impact of Mel was significantly (*p <* 0.001) compromised in si G6PD models (Figure 4J,K).

### 2.5 Mel Relieves Pb‐Induced mtDNA Leakage via Diminishing Immoderate Mitochondrial Fission

**Figure 5:**
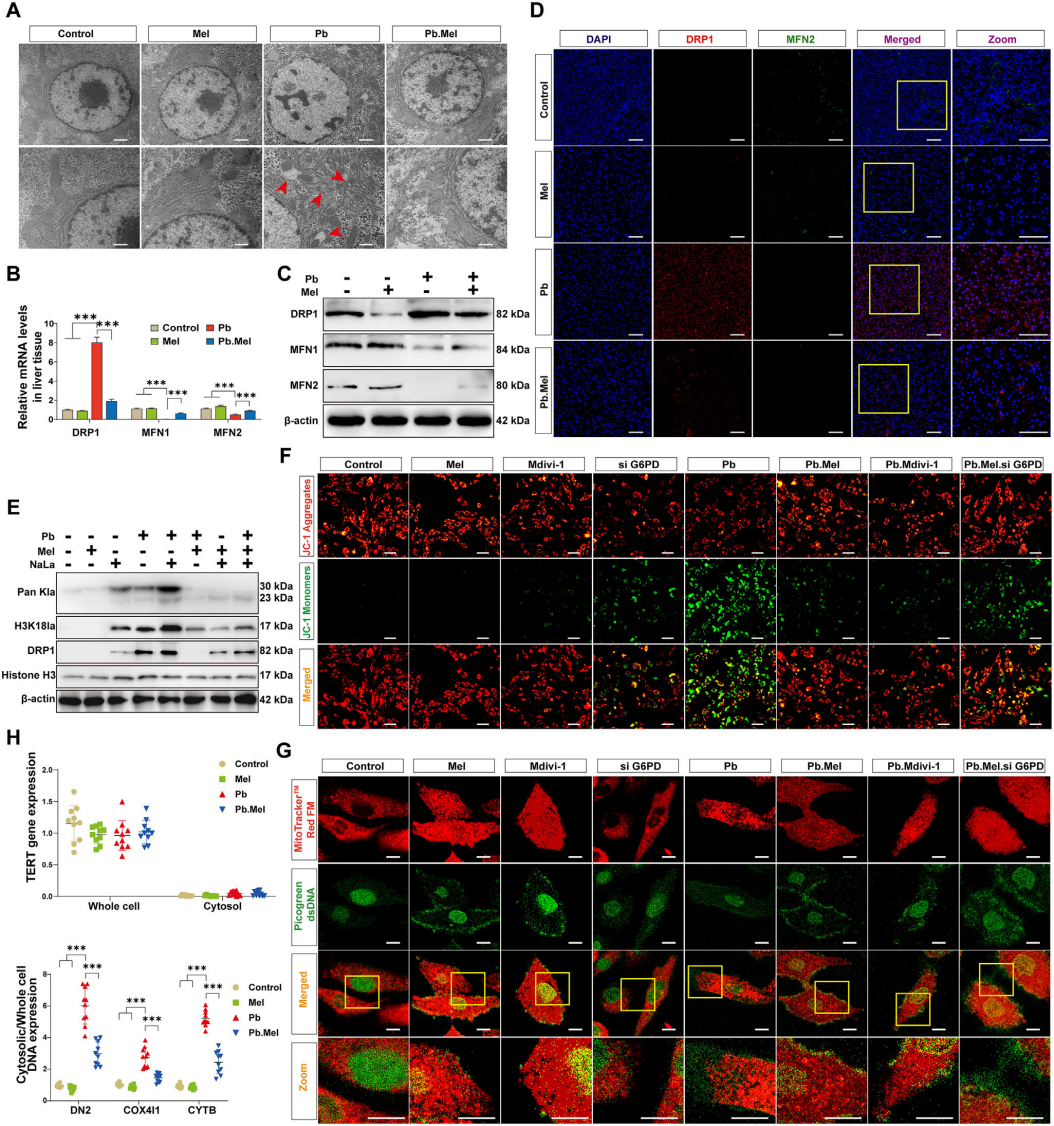
Mel inhibits mitochondrial fission and alleviates Pb‐triggered mtDNA leakage. A) The ultrastructural observation carp liver. B) The mRNA expression of mitochondrial‐dynamic balance‐related genes (*n* = 6) by two‐way ANOVA. C) Protein level of DRP1, MFN1 and MFN2 genes in liver tissue. D) IF results of DRP1 (Red), MFN2 (Green) and DAPI (Blue) in liver (Scale bar = 50 µm). E) The lactylation modification correlated with DRP1 expression. F) The JC‐1 staining of the cell models (Scale bar = 20 µm). G) The observation of mtDNA leakage by staining with Picogreen dsDNA quantitation reagent (Green) and MitoTracker Red dye (Red) (Scale bar = 5 µm). H) Quantification of nuclear gene (TERT) expression and cytoplastic mtDNA levels (*n* = 10) by two‐way ANOVA.

According to our previous findings of Mel‐mediated mitigation of Pb‐induced mitochondrial dysfunction, we further determined ultrastructural alterations using transmission electron microscopy (TEM). As shown in **Figure** **5**A, evident mitochondrial swelling, cristae disruption, and excessive mitochondrial fission (red arrowhead) were evident in the Pb group, all of which were remarkably alleviated in the Pb.Mel group. Additionally, the expression of mitochondrial dynamic balance‐related genes was estimated in mRNA and protein‐dependent manner, revealing the enhanced expression of DRP1 and eliminated level of MFN1 and MFN2 upon Pb exposure, both of which were effectively reversed by Mel supplementation, elucidating that the Pb‐intensified immoderate mitochondrial fission was alleviated by Mel (Figure 5B–D; Figure S6A, Supporting Information). Mechanistic investigations demonstrated coordinated elevation of DRP1 and H3K18la following Pb/NaLa treatments, while both parameters were suppressed by Oxamate and Mel treatment (Figure 5E; Figure S6B, Supporting Information), implying that H3K18la active DRP1 expression under Pb treatment. Furthermore, the mitochondrial membrane potential (ΔΨm) contributed to the expected retrievement by Mel and Mdivi‐1 supplement upon Pb exposure (Figure 5F; Figure S6C,D, Supporting Information). Given the evident overlapping between mitochondrial fission and mtDNA leakage, the Picogreen double‐stranded DNA (dsDNA) quantitation reagent, along with MitoTracker Red dye, was applied in Figure 5G. The confocal images revealed an obvious intensification in the amount of dsDNA staining areas without overlapping with stained mitochondria under Pb exposure, implying that Pb deteriorates mitochondrial nucleoid architecture, disorganization, and exacerbates mtDNA leakage. Crucially, Mel and Mdivi‐1 co‐treatment markedly attenuated mtDNA leakage, while this mitigation effect was interrupted in si G6PD models, illuminating that Mel functions as a G6PD‐dependent regulation of fission‐mediated mtDNA release. Additionally, Tert expression was not detected in the cytoplasmic DNA, implying that the presence of dsDNA in the cytoplasm did not derive from the nucleus, and revealing a significant increase in intact mtDNA levels (ND2, COX4I1, and CYTB) in the cytosolic fraction in the Pb group (Figure 5H). Therefore, Pb‐induced accumulation of mtDNA was attenuated by Mel treatment.

### 2.6 Mel Inhibits Mitochondrial Fission to Alleviate Pb‐Induced Pyroptosis via cGAS‐STING Axis

**Figure 6:**
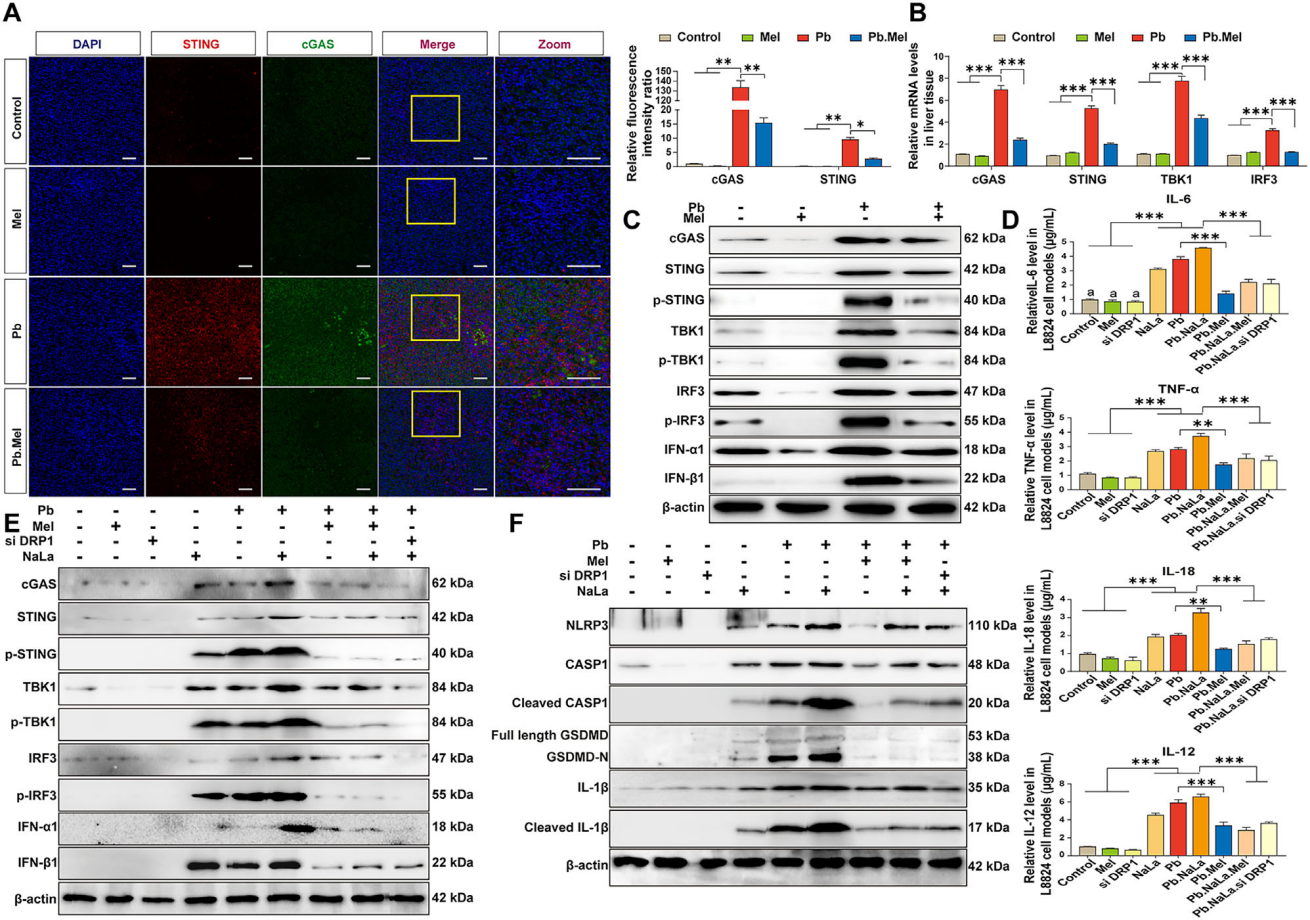
Mel suppresses Pb‐mediated abnormal mitochondrial fission and pyroptosis through cGAS‐STING axis. A) IF results and quantification of STING (Red), cGAS (Green) and DAPI (Blue) in liver (Scale bar = 50 µm). B) The mRNA levels of cGAS‐STING signaling pathway‐related genes (*n* = 6) by two‐way ANOVA with Tukey's multiple comparison test. C) Protein levels of genes related to cGAS‐STING signal. D) The content of inflammatory cytokines in cell models (*n* = 6) by one‐way ANOVA. E) The protein level of cGAS‐STING signaling‐related genes in vitro. F) The protein expression of pyroptosis‐related genes in cell model.

**Figure 7:**
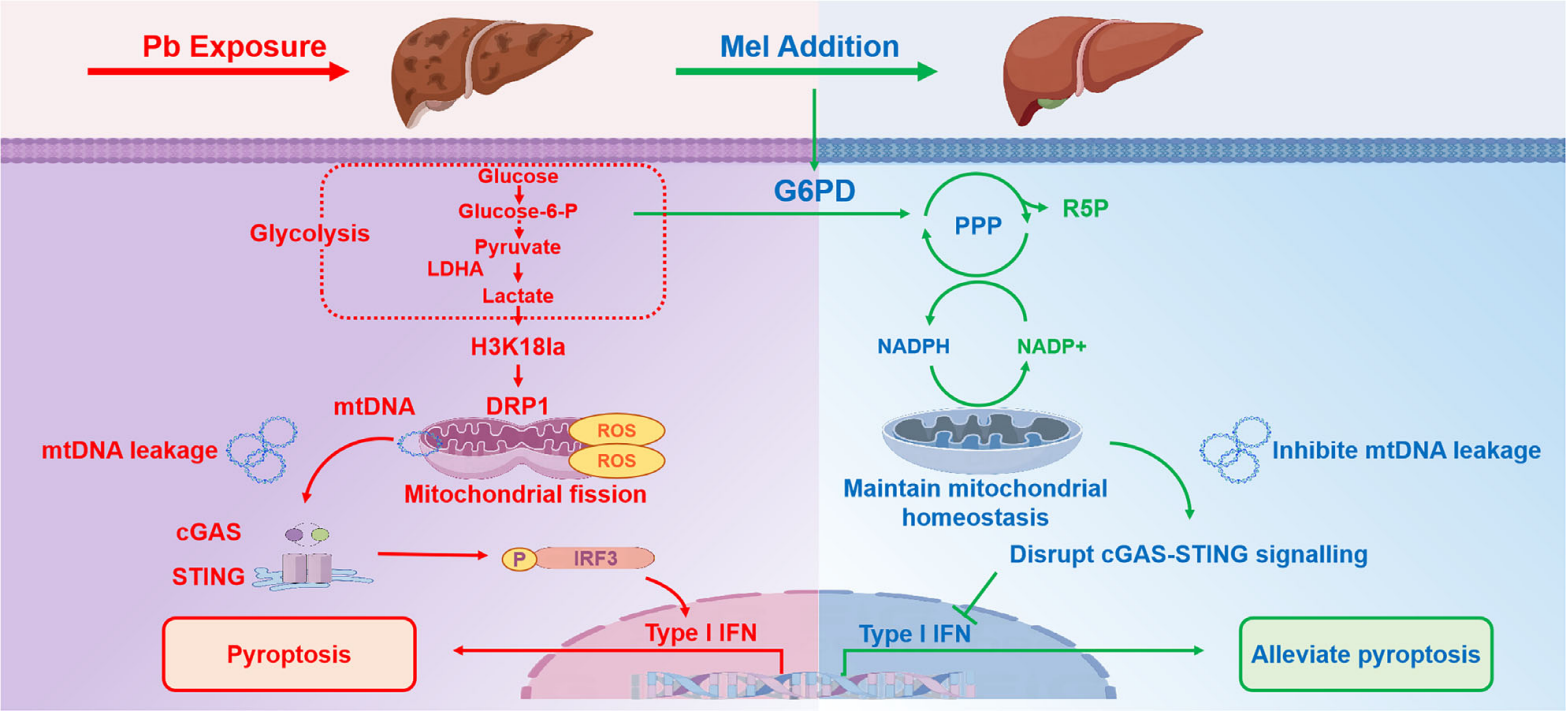
The working model illustrating Mel alleviating Pb‐induced hepatic inflammatory damage progression by targeting G6PD to eliminate Pb‐elevated glycolysis and H3K18la, suppress cGAS‐STING signaling activation by disrupting Pb‐derived DRP1‐dependent mitochondrial fission and mtDNA leakage, ultimately attenuate Pb‐triggered liver injury.

To assess the role of cGAS‐STING signaling in Pb‐induced hepatotoxicity, dual‐immunofluorescence staining of anti‐cGAS and anti‐STING revealed enhanced (*p <* 0.01) cGAS/STING signals in the Pb group and moderated with Mel addition (**Figure** **6**A). Moreover, the expression of cGAS‐STING signaling‐related genes was continuously evaluated in both mRNA and protein‐dependent manners (Figure 6B,C), with the aggregation of cGAS, p‐STING, p‐TBK1, p‐IRF3, IFN‐α1, and IFN‐β1 detected upon Pb exposure. Also, it was reduced (*p <* 0.05) as expected with Mel addition, indicating the participation of cGAS‐STING signaling participates in the Pb‐mediated hepatocytes inflammatory response. To further confirm the regulatory role of cGAS‐STING on pyroptotic cell death and inflammatory response, the si STING knockdown models were established (Figure S7, Supporting Information). Key pyroptosis‐related proteins, including NLRP3, Cleaved CASP1, GSDMD‐N, and Cleaved IL‐1β, were markedly down‐regulated in si STING models upon Pb exposure as compared to the Pb group. Furthermore, Pb‐induced upregulation of pro‐inflammatory cytokines (IL‐6, IL‐8, TNF‐α, IL‐12) and chemokine CXCL10 was substantially reversed in the si STING model. These data collectively demonstrate that cGAS‐STING pathway activation is indispensable for Pb‐driven hepatic inflammation. Consistent with these findings, the content of typical pro‐inflammatory cytokines was significantly (*p <* 0.001) increased with Pb and NaLa, which was notably (*p <* 0.05) mitigated with Mel addition or in si DRP1 knockdown models (Figure 6D; Figure S7, Supporting Information). These observations correspond to the consistent expression patterns of genes related to the cGAS‐STING signaling pathway and pyroptosis, identifying that Mel plays an anti‐inflammatory role by regulating DRP1 to inhibit hepatocyte pyroptotic death via cGAS‐STING axis (**Figure** **7**).

## 3 Discussion

Although extensive studies have been conducted worldwide on Pb residues and their toxicity in freshwater ecosystems, our understanding of effective mitigation strategies and detoxification mechanisms remains limited. With continuous investigation, the metabolic toxicity caused by Pb has been gradually recognized, while research focus remains disproportionately skewed toward neurotoxicity compared to hepatic metabolic dysregulation. Given that the liver serves as the primary bioaccumulative organ of Pb in fish and modulates crucial metabolism processes, this study systematically investigated Pb hepatotoxicity mechanisms in common carp. However, Mel has been characterized with its critical role in the detoxification mechanism and hepatoprotective properties, the underlying mechanism remains elusive. In this study, we found that Mel efficiently alleviates Pb‐induced hepatic function impairment. And integrated transcriptomic and targeted metabolomic analyses identified glycolytic reprogramming as a hallmark of Pb‐induced pyroptosis. Mechanistically, we elucidated that Mel targets G6PD to alleviate Pb‐triggered glycolysis and lactylation modification. Notably, the exacerbated mitochondrial dysfunction and mtDNA leakage induced by Pb were effectively ameliorated by Mel addition.

Our primary findings from GSEA analysis in the transcriptomics of carp liver revealed significant enrichment of glycolytic pathways following Pb exposure, leading us to hypothesize that Pb induces hepatic inflammation through glycolysis‐driven metabolic reprogramming to potentiate hepatotoxicity. Several lines of evidence support that glucose metabolism disorder is the key driver of toxicity caused by Pb exposure. A recent study reported that the impairment of glucose metabolism aggravated in Pb‐exposed mice.^[^ ^31^ ^]^ And the elevated glycolysis was also found in Pb‐treated *Rana chensinensis* larvae.^[^ ^32^ ^]^ Consistent with these findings, Pb exposure causes a remarkable shift from OXPHOS to glycolysis in SH‐SY5Y cell.^[^ ^33^ ^]^ Our results revealed that Pb exposure concurrently elevates Pyruvate and Acetyl‐CoA levels with distinct magnitudes of increase, as Pyruvate accumulation exceeds that of Acetyl‐CoA. This observation suggests two critical insights: 1) Pb exposure potently enhances pyruvate production through glycolytic activation; 2) The conversion of Pyruvate to Acetyl‐CoA is likely subject to multi‐tiered regulatory constraints. Mechanistically, we identified that Pb exposure significantly up‐regulates PDK1 expression, which phosphorylates and inhibits pyruvate dehydrogenase (PDH) complex activity, thereby restricting Pyruvate entry into the TCA cycle. Notably, while this study focused on lactate accumulation induced by Pb exposure, its downstream effects on TCA cycle dynamics remain unexplored, representing a limitation of the current research. Building upon these findings, we further demonstrated that activated glycolysis with elevated lactate content was a hallmark of Pb‐exposed groups, culminating in enhanced histone lactylation. It is widely known that histone lactylation has been demonstrated to play a crucial role in maintaining physiological states and initiating various pathological states through regulating gene transcription.^[^ ^18^, ^34^ ^]^ Building on these insights, we subsequently found that elevated H3K18la enhances DRP1 expression, which complements the mechanism by which Pb can induce excess mitochondrial fission found in previous studies.^[^ ^35^, ^36^ ^]^ Therefore, our findings advance the understanding of the mutual regulation of metabolism and inflammatory response on the metabolism‐epigenetics axis upon Pb exposure. Notably, multiple studies have revealed that Mel exerts a beneficial effect on aerobic glycolysis suppression and is directly relevant to cell survival.^[^ ^37^, ^38^ ^]^ A recent study refers that Mel maintains mitochondrial integrity and elevates glycolysis in mesothelial cells,^[^ ^39^ ^]^ but studies of the underlying mechanisms remain unexplored. Mechanically, several lines of evidence support that G6PD plays an important role in the modulation of Mel on glucose metabolism.^[^ ^40^, ^41^ ^]^ We predicted a potential interaction between Mel and G6PD, which was subsequently validated by CETSA experiments. However, this stabilization effect diminished sharply ≈53 °C, likely because the interaction is mediated by hydrogen bonds and hydrophobic interactions, which are highly sensitive to thermal disruption. Furthermore, the enzymatic activity and protein levels of G6PD in the Mel‐treated group were significantly elevated compared to those in the Control group, indicating that Mel exerts dual regulatory effects on G6PD, encompassing both direct binding and upregulated expression. Conceivably, the attenuated effects of Mel on glycolysis and lactate accumulation were abrogated in the si G6PD model, further supporting G6PD as a pivotal mediator of Mel‐induced metabolic reprogramming. Although prior studies have reported anti‐fission properties of Mel,^[^ ^42^, ^43^ ^]^ their focus has centered on correcting mitochondrial dynamic imbalances and functional deficits, leaving its epigenetic regulatory role unexplored. Our results suggested that Pb‐induced excessive mitochondrial fission is attributed to glycolysis‐derived H3K18la and DRP1 expression enhancement, and then Mel performed moderating effects on mitochondrial fragmentation by reducing the level of lactylation modification. Meanwhile, Mel displayed a significant inhibitory effect on H3K18la and DRP1 expression under NaLa treatment. These findings provide new insights into the mechanism of Mel treatment, indicating new prospects for the further study of mitochondria.

The immoderate mitochondrial fission is considered to be the initial event of Pb‐induced mitochondrial dysfunction,^[^ ^36^, ^44^, ^45^ ^]^ characterized by persistent overproduction of mtROS.^[^ ^46^ ^]^ Our results consistently showed mitochondrial fragmentation observed by ultrastructure analysis, elevated mtROS levels, and depletion of mtATP and ΔΨm under Pb exposure, indicating that Pb mediated mitochondrial fission and dysfunction. Then, it has been considered that DRP1‐dependent mtDNA leakage is required for cGAS‐STING signaling activation.^[^ ^34^, ^47^ ^]^ Analogously, the cGAS‐STING pathway is sensitive to DRP1 overexpression.^[^ ^47^ ^]^ Our results revealed that a large amount of mtDNA was translocated from mitochondria to the cytoplasm in the Pb group, as shown by costaining with Picogreen dsDNA quantitation reagent and MitoTracker Red dye. Moreover, the expression peaks of cGAS and STING were also located in the Pb group, suggesting that Pb exposure obviously facilitated mtDNA release and triggered the activation of cGAS‐STING signaling. Subsequently, cGAS‐STING signaling has emerged as a key mediator of inflammation and has been implicated in the regulation of tissue inflammatory damage.^[^ ^48^ ^]^ The leakage of mtDNA and the activation of cGAS‐STING signaling have been verified as contributing factors to the progression of liver fibrosis.^[^ ^49^ ^]^ Meanwhile, the contribution of cGAS‐STING axis and interferon genes in the inflammatory response of bovine hepatocytes treated with hydrogen peroxide has been illuminated.^[^ ^50^ ^]^ Additionally, elevated cGAS‐STING signaling enhanced the secretion of inflammatory cytokines such as TNF‐α and IL‐1β in mice with lipopolysaccharide‐induced liver injury.^[^ ^47^ ^]^ Indeed, both in vivo with Pb exposure and in vitro with Pb and/or NaLa exposure, enhanced expression of cGAS‐STING‐related genes (TBK and IRF3) and IFN responder‐related genes was detected, while a significant elimination was observed in si DRP1 cell models. It was further demonstrated that Pb activated cGAS‐STING and its downstream signal through intensified lactylation modification and DRP1 expression. Our results also show that pyroptosis is a responsive event that depends on the cGAS‐STING signal, which is consistent with previous studies,^[^ ^51^, ^52^ ^]^ and exacerbates Pb‐mediated inflammation by generating inflammatory cytokines. The anti‐inflammatory effect of Mel is particularly prominent, among which the inhibition of cGAS‐STING signaling pathway^[^ ^31^, ^53^ ^]^ and the suppression of pyroptotic cell death are also extremely evident.^[^ ^39^, ^54^ ^]^ Therefore, based on the previous results, Mel down‐regulates glycolysis‐dependent mitochondrial fission, which can alleviate cGAS/STING‐mediated inflammation. Supporting this hypothesis, we observed that Mel supplementation up‐regulated the NADPH content and further restored mtATP and ATP levels that were depleted under Pb exposure. Given that NADPH provides high‐energy electrons for the OXPHOS process and plays an important role in reduction metabolism,^[^ ^55^ ^]^ Mel promotes NADPH to attenuate Pb‐induced mitochondrial dysfunction by enhancing electron transport chain efficiency via NADPH‐dependent antioxidant systems.^[^ ^56^, ^57^ ^]^ Additionally, we found that compared with Pb and/or NaLa exposure, the expression of pyroptosis‐related genes and content of inflammatory cytokines decreased, while these benefits were disrupted in the si DRP1 model, implying that DRP1‐dependent mitochondrial homeostasis is an integrating factor for the mitigated function of Mel on glycolysis‐derived pyroptosis and the generation of inflammatory cytokines. Our study elucidated for the first time the mitigating role of Mel in alleviating pyroptosis‐derived inflammatory damage via DRP1‐cGAS‐STING axis, providing an experimental basis for subsequent research.

## 4 Conclusion

In summary, this study provides novel insights into glycolytic metabolism reprogramming‐epigenetic regulation in response to Pb exposure. It can be concluded that Pb‐enhanced hepatic lactate accumulation drives mitochondrial fission through histone lactylation modifications, particularly H3K18la, thereby regulating DRP1 expression. This subsequently enhances mtDNA leakage and promotes pyroptosis via the cGAS‐STING pathway. Notably, our study presents the first evidence that Mel ameliorates glycolytic dysregulation through G6PD targeting, conferring sustained protection against Pb‐induced hepatotoxicity. This is achieved by suppressing DRP1‐mediated mitochondrial fission, reducing mtDNA release, and attenuating inflammatory responses via the cGAS‐STING axis. Collectively, our findings establish that Mel alleviates Pb‐induced hepatocytes pyroptosis in common carp by modulating glucose metabolism reprogramming through G6PD targeting.

## 5 Experimental Section

### Study Approval

The animal welfare and experimental protocols strictly adhered to the guidelines set by the Committee for the Protection and Use of Laboratory Animals of Northeast Agricultural University (NEAUEC20230155620).

### Reagents

Lead nitrate (Cat#: 467790), Melatonin (Cat#: M5250), Uranyl acetate (Cat#: 201030), 2.5% glutaraldehyde (Cat#: G5882), 1% periodic acid (Cat#: 3951), Ethanol (Cat#: 459828), Acetone (Cat#: 179124), Paraffin (Cat#: 1.07151), Resin (Cat#: 45345), 3% H_2_O_2_ (Cat#: 88597) and Schiff reagent (Cat#: S5133) were purchased from Sigma. 4% paraformaldehyde (Cat#: P0099), Hematoxylin (Cat#: C0107), DAPI (Cat#: C1006) and NP‐40 lysis buffer (Cat#: P0013F)were purchased from Beyotime. Leibovitz's L‐15 medium (Cat#: BL313A) was purchased from Biosharp. Fetal bovine serum was purchased from Biological Industries. Penicillin‐Streptomycin Solution (Cat#: 15140122), Lipofectamine 2000 (Cat#: 11668500), Opti‐MEM (Cat#: 31985070), MitoSOX Red (Cat#: M36008), MitoTracker Red (Cat#: M7512) were purchased from Thermo Fisher Scientific. Picogreen dsDNA quantitation reagent (Cat#: 12641ES01) was purchased from Yeasen. Ac‐YVAD‐cmk (Cat#: HY‐16990), Oxamic acid sodium (Oxamate; Cat#: HY‐W013032A), N‐Arachidonoyl‐L‐alanine (NaLa; Cat#: HY‐134545) and Mdivi‐1 (Cat#: HY‐15886) were purchased from MCE.

### Pb and/or Mel Exposure Models Establishment

A total of 120 common carps (226 ± 8.62 g) were obtained from a commercial fish pond upstream of the Songhua River in Harbin, China, and randomly distributed into the laboratory tanks (60 cm × 30 cm × 26 cm) filled with 15 L dechlorinated water that had been continuously aerated for 72 h to eliminate excess chlorine. Each experimental group consisted of 6 parallel tanks (*n* = 6), with 5 fish housed per tank. After two weeks of adaptation, four treatment groups were established by random division of the laboratory tanks, and were set up as the Control group without any addition, Mel group with 0.2 mg L^−1^ Mel, Pb group with 0.5 mg L^−1^ Pb(NO_3_)_2_ based on local aquatic environmental concentrations^[^ ^58^, ^59^ ^]^ and Pb.Mel group both 0.5 mg L^−1^ Pb(NO_3_)_2_ and 0.2 mg L^−1^ Mel added together. The fish were fed twice daily with half‐volume water replacement using dechlorinated tap water, and adding Pb and or Mel to the water body according to the dose. At the end of the experiment, after a duration of 30 days, the carp were humanely euthanized. The body cavity was opened by a ventral midline incision from the cloaca to the posterior edge of the operculum, carefully avoiding damage to internal organs. The liver was gently separated from the gut and other adjacent tissues using blunt forceps and excised intact into a pre‐chilled sterile Petri dish. Samples were aliquoted for further experiments.

### Pb Distribution Map

To identify the residue of Pb in the world, literature from 2023–2024 was systematically reviewed through the NCBI website (https://www.ncbi.nlm.nih.gov/) and China National Knowledge Infrastructure (http://www.cnki.net) with keywords “Pb”, “Environment”, and “Contaminate”. From 330 eligible studies (summarized in Table S1, Supporting Information), a geographical distribution map of Pb residues was constructed. Additionally, 2022 refined Pb production data from the International Lead and Zinc Study Group (https://www.ilzsg.org/) were analyzed to generate corresponding geographical maps and histograms (Table S2, Supporting Information).

### Cell Model Establishment

The L8824 (grass carp hepatocytes) cell line used in this study was purchased from American Type Culture Collection (ATCC) and stored in the liquid nitrogen cell storage container. Upon obtaining, the cryovial was rapidly thawed in a 37 °C water bath, and the cell suspension was transferred to 8 mL of Leibovitz's L‐15 medium supplemented with 10% fetal bovine serum and 1% Penicillin‐Streptomycin Solution. The cells were then routinely incubated at 27 °C with 5% CO_2_. To assess the cytotoxicity of Pb on L8824 cells, the Cell Counting Kit‐8 (CCK8; Beyotime Biotechnology, China) assay was employed. The cells were incubated in 96‐well plates and exposed to different concentrations of Pb and Mel. Based on the results of cell viability (Figure S8, Supporting Information), concentrations of 50 µm Pb and/or 6 nm Mel were selected for subsequent incubation with L88244 cells. Detailed experimental methods can be found in the supplementary materials. The siRNAs were synthesized by RiboBio Co., Ltd. Subsequently, si DRP1 plasmid, si G6PD plasmid, si STING, or negative control (NC) plasmid were respectively transfected into L8824 cells with Lipofectamine 2000 in 1 mL of Opti‐MEM medium.

### Histological, Ultrastructural, and Immunofluorescence Observations

To ascertain the histological and ultrastructural changes in liver tissue caused by Pb and Mel, H&E staining and TEM were utilized in this study. Liver samples were primarily fixed in 4% paraformaldehyde or 2.5% glutaraldehyde solution, dehydrated with ethanol and/or acetone. After complete dehydration, the liver samples were embedded in paraffin blocks and sectioned at 5 µm thickness for H&E staining. The remaining samples were resin embedded for TEM and ultrathin‐sectioned at 50 nm with a diamond knife on an ultramicrotome. The sections were collected on copper grids, then sequentially stained with uranyl acetate and lead citrate. And the samples were observed by transmission electron microscope (H‐7800, HITACHI, Japan) or optical microscope (Olympus, Japan).

For immunohistochemistry (IHC), antigen retrieval was performed by microwave heating in citrate buffer, followed by endogenous peroxidase blocking with 3% H^2^O^2^ Sections were incubated with anti‐G6PD antibody (1:200, Servicebio, China) overnight at 4 °C, then treated with HRP‐conjugated secondary antibody (1:500, Servicebio, China) and visualized using DAB substrate. PAS staining was conducted by oxidizing glycogens with 1% periodic acid for 10 min, reacting with Schiff reagent for 30 min, and counterstaining with hematoxylin.

To examine the effects of Pb and Mel on the hepatocytes in common carps, dual‐immunofluorescence staining method was employed in this study by following the instructional protocols. The tissues were embedded in paraffin, and sectioned into slices to respectively incubated with primary antibodies of anti‐NLRP3 (Servicebio, China) and anti‐GSDMD (Servicebio, China), anti‐DRP1 (Servicebio, China) and anti‐MFN2 (Servicebio, China), anti‐cGAS (Servicebio, China) and anti‐STING (Servicebio, China) at 4 °C overnight. Subsequently, secondary antibodies were applied to the slices, followed by re‐staining with DAPI (Servicebio, China). The fluorescence microscope (Thermo Fisher Scientific, USA) was operated to observe and capture images.

### Inflammatory Cytokines and Glucose Metabolites Determination

The content of inflammatory cytokines was assessed by ELISA kits, following the instructions. And glucose metabolites and enzymes with commercial kits from Beijing Solarbio Science & Technology Co., China, including Glucose content Assay Kit (BC2505), Acetyl‐CoA Content Assay Kit (BC0980), L‐Lactate Content Assay Kit (BC2230), and Pyruvate Content Assay Kit (BC2205). Then the content of Glucose 6 Phosphate (Glucose‐6‐P, AB83426) and Glucose 6 Phosphate Dehydrogenase (G6PD, AB102529) were determined with commercial kits from Abcam (Cambridge, UK) following standard protocols.

### Oxidative Parameters and ATP Determination

The liver tissues were homogenized and then subjected to centrifuge at 2500 rpm for 10 min and supernatants were analyzed for oxidative stress markers including ROS (E004‐1‐1), Catalase (CAT, A007‐1‐1), Total Antioxidative Capacity (T‐AOC, A015‐1‐2), Total Superoxide Dismutase (SOD, A001‐3‐1), Inducible Nitric Oxide Sythase (iNOS, A014‐1‐2), Nicotinamide Adenine Dinucleotide Phosphate (NADPH, A115‐1‐1) and Reduced Glutathione (GSH, A006‐1‐1) using Nanjing Jiancheng Bioengineering Institute Kits. The ATP levels in the groups and L8824 cell models were evaluated using the ATP assay kit (A095‐1‐1, Nanjing Jiancheng, China). Subsequently, the mtROS and mtATP levels were primarily determined by employing the tissue Mitochondria Isolation Kit (C3606, Beyotime, China), followed by the ROS Assay Kit (E004‐1‐1, Nanjing Jiancheng, China) or ATP Assay Kit.

The generation of mtROS in various L8824 cell models was also observed using MitoSOX Red. The L8824 cells in each group underwent pre‐treatment and were seeded into 6‐well plates. After incubation with MitoSOX Red (5 µm) for 10 min at 27 °C, the cells from each group were washed three times with PBS buffer. Then, the cells in each group were counterstained with DAPI (Beyotime Biotech., China) for 10 min. Fluorescence images were acquired using a fluorescence microscope.

### Mitochondrial Function and DNA Integrity Assessment

Mitochondrial membrane potential (MMP) was determined using JC‐1 Assay Kit (Beyotime Biotechnology, China) by following the instructional protocols. The emission of JC‐1 monomers (green fluorescence, 530 nm excitation/590 nm emission) and JC‐1 aggregates (red fluorescence, 485 nm excitation/535 nm emission) was observed using a fluorescence microscope (Thermo Fisher Scientific, USA) and flow cytometry (BD Biosciences, USA).

The TIANamp Genomic DNA Kit (Tiangen Biotech, China) was conducted according to established protocols and described previously.^[^ ^11^ ^]^ to purify total cellular DNA. For cytosolic DNA fractionation, ice‐cold 0.1% NP‐40 lysis buffer was applied for 20 min. Following centrifugation at 15 000 rpm (4 °C, 20 min), the cytosolic fraction was subjected to identical DNA purification procedures. Mitochondrial DNA quantification was performed with mitochondrially encoded genes, including NADH dehydrogenase subunit 2 (ND2; NC_001606.1); cytochrome c oxidase subunit 4I1 (COX4I1; LOC109049772); cytochrome c reductase cytochrome b (CYTB; NC_018035.1), and using telomerase reverse transcriptase (TERT; XM_042745372.1) as the normalization control (Table S3, Supporting Information).

To observe the mtDNA leakage of each group, MitoTracker Red and Picogreen dsDNA quantitation reagent were applied. Cells in each group were initially rinsed with PBS and incubated with MitoTracker Red (0.2 µm) for 30 min, then counter‐stained with Picogreen working solution (1:200) for 10 min. Images were captured using a fluorescence microscope.

### Transcriptomics and Targeted Metabolomics Analysis

The global transcription profiling of the common carp liver tissues was performed by Wuhan Bioacme Co., Ltd, following the operational instructions (Tables S4 and S5, Supporting Information). Illumina HiSeqTM2000 was applied to establish the sequencing library, and obtained raw sequencing reads were primarily filtrated to the Clean Data by quality control (Fastp V0.19.4). HISAT2 (v2.2.1) software was employed to align Clean Data with the common carp reference genome, as well as featureCounts v2.0.3 was operated to calculate reads. DESeq2 (v1.34.0) was used for standardization and differential analysis, clusterProfiler (v4.2.2) for KEGG and GO enrichment, and GSEA (v4.3.2) with Hallmark gene sets were used for pathway enrichment analysis. The DEGs were preliminarily screened using thresholds of log_2_|Fold change| > 1 and adjusted *p*‐values *<* 0.05 (FDR < 0.05) (Tables S6 and S7, Supporting Information). The pheatmap (v1.0.12) with z‐score normalization and EnhancedVolcano (v1.14.0) were respectively performed for visualization of Heatmap and Volcano plot. For targeted metabolomics analysis, the metabolites were ormarly etracted from liver samples with 80% methanol‐water with ultrasonication.

### Real‐Time Quantitative PCR (RT‐qPCR)

Total RNA extraction, complementary DNA (cDNA) synthesis, and qRT‐PCR were performed according to the instructions of the BioRT mRNA cDNA Synthesis Kit (Bioer Technology, China) as introduced previously.^[^ ^60^ ^]^ The primer sequences for qRT‐PCR are provided in Table S8 (Supporting Information). β‐actin was used as an internal reference. The relative mRNA abundances were calculated using 2^−ΔΔCt^ method.

### Western Blot Analysis

The western blot technique was used to visualize the protein levels of the following indicators in carp liver and L8824 cells. Referring to the previous study,^[^ ^61^ ^]^ total protein was extracted with PMSF (Beyotime Biotechnology, China). And protein bands were transferred to PVDF membranes at 200 mA for 60–130 min. After blocking, the bands were incubated with diluted primary antibody overnight. The used antibodies dilution factors were provided in Table S9 (Supporting Information). After incubating with secondary antibodies against rabbit IgG (1:10000, Immunoway, China), the protein band intensities were quantified by using chemiluminescence system (Applygen Technologies, China), X‐ray films (TransGern Biotech, China), and Image J software.

### Statistical Analysis

All statistical analyses were conducted using GraphPad Prism software (version 8.2, Graph Pad Software, USA). To compare the responses mediated by Pb and Mel in vivo and in vitro, one‐way or two‐way analysis of variance (ANOVA) with Tukey's multiple comparison test was employed. The data homogeneity and variance normality were assessed using Levene's test and the Shapiro‐Wilk test, respectively. And all data were performed as mean ± standard deviation (SD), and statistically significant differences were considered at *p <* 0.05, indicated by ^*^, ^*^ *p <* 0.05, ^**^ *p <* 0.01, and ^***^ *p <* 0.001.

## Conflict of Interest

The authors declare no conflict of interest.

## Author Contributions

Z.Y.M. and X.F.J. contributed to conceptualization, methodology, investigation, and writing of the original draft. L.W. and Z.R.M. were responsible for visualization, funding acquisition, writing, reviewing, and editing. S.W.X. handled project administration and supervision. All authors approved the final version of this manuscript.

## Supporting information



Supporting Information

## Data Availability

The data that support the findings of this study are available from the corresponding author upon reasonable request.
