# Correction to “Melatonin Modulates Glucose Metabolism Reprogramming via Targeting G6PD to Alleviate Lead‐Induced Hepatocytes Pyroptosis in Common Carp (*Cyprinus carpio* L.)”

**DOI:** 10.1002/advs.74877

**Published:** 2026-03-23

Zhiying Miao, Xiaofeng Ji, Lai Wei, Zhiruo Miao, Shiwen Xu^*^. Melatonin Modulates Glucose Metabolism Reprogramming via Targeting G6PD to Alleviate Lead‐Induced Hepatocytes Pyroptosis in Common Carp (*Cyprinus carpio* L.).

DOI: 10.1002/advs.202501041. Adv. Sci., November 2025, 12(41), e01041.

During assembly of the PAS staining image, an inappropriate field of view was selected. As a result, the high‐magnification inset was not fully contained within the low‐magnification overview, causing misalignment. The corrected image 3(G) is shown below.


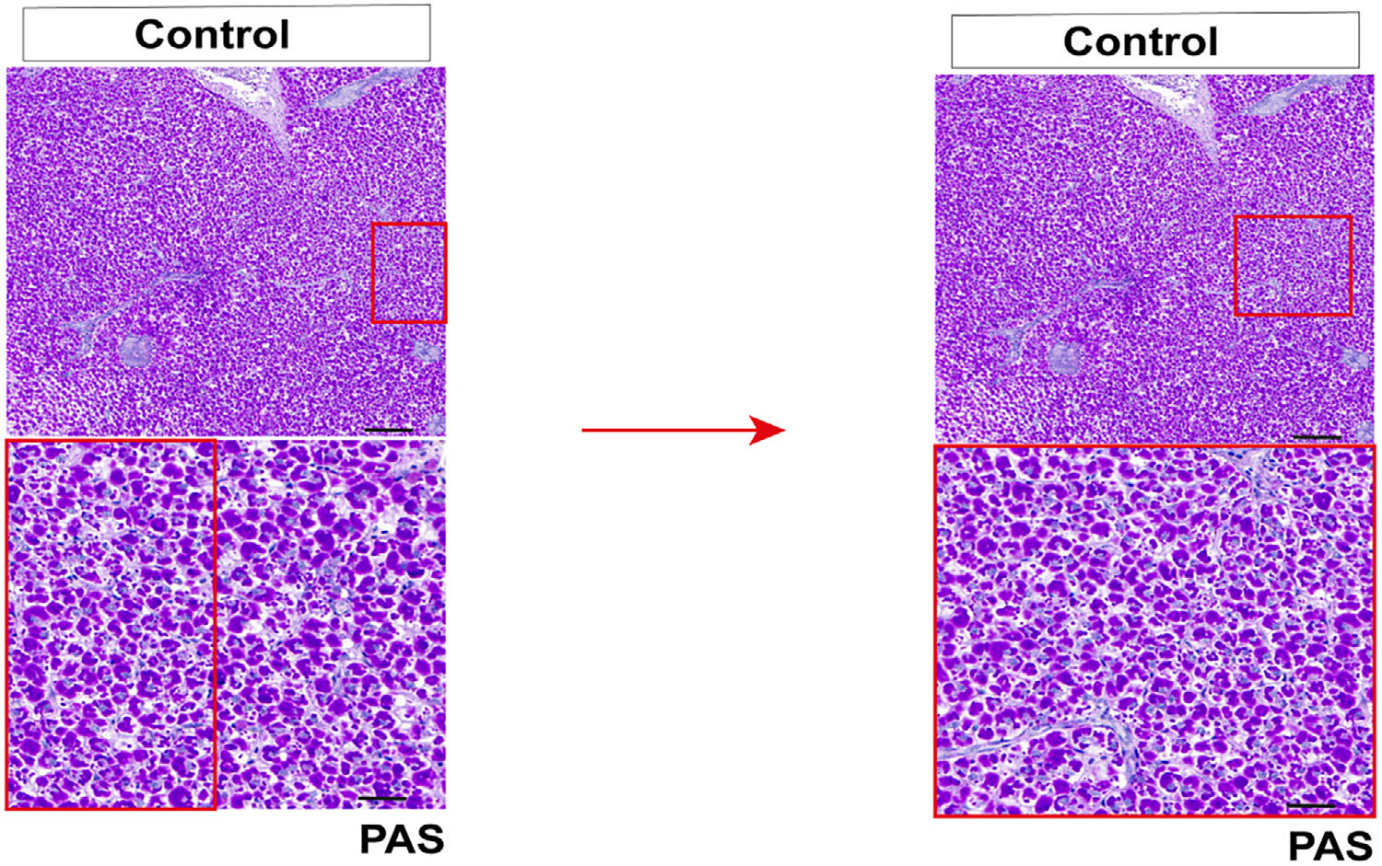


Figure 3 (G) PAS staining of carp liver (Scale bar = 50 µm/20 µm in upper/lower panel).

Corrected Figure 3 is provided below.


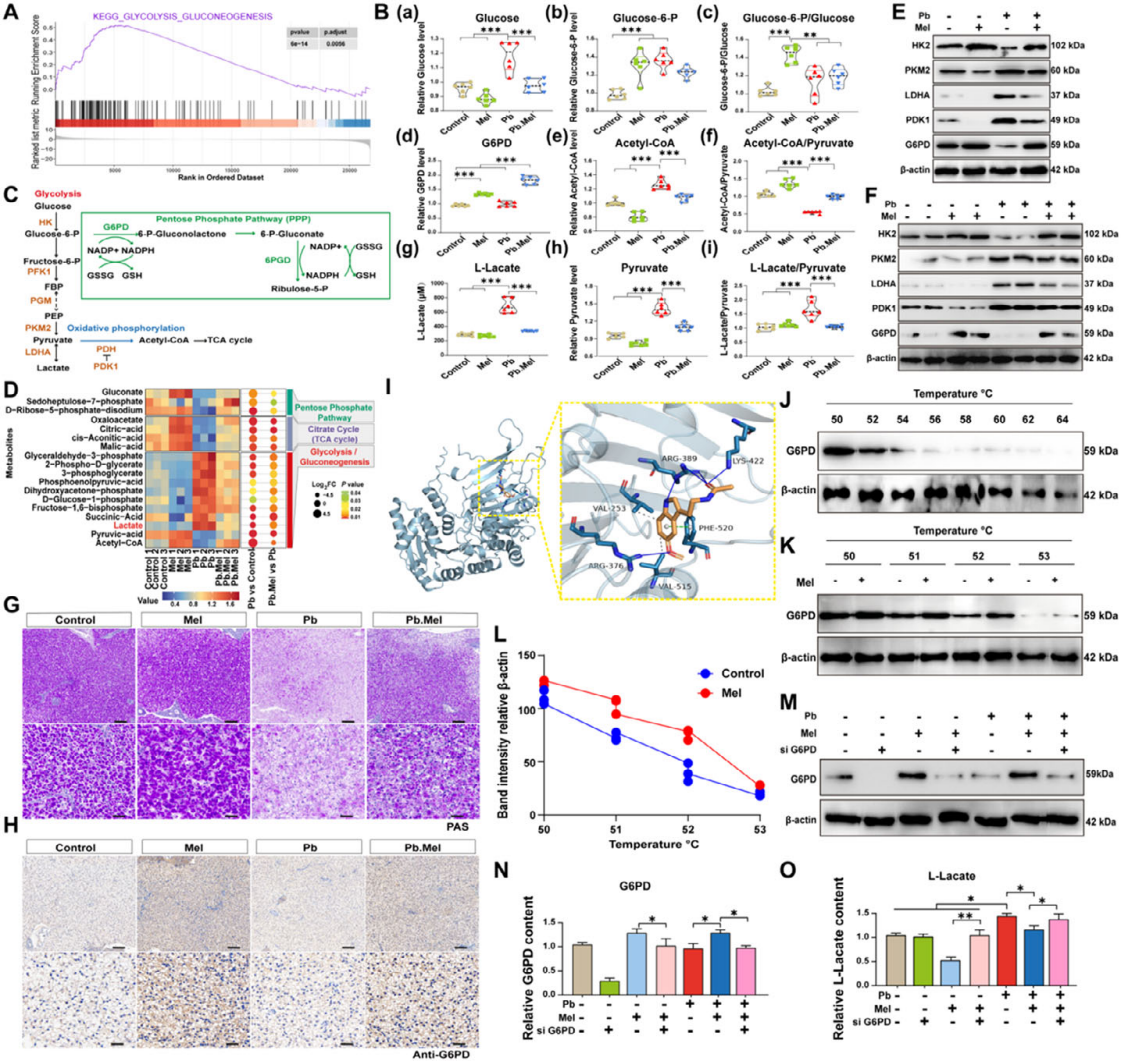


**Figure 3**. Mel targets G6PD to alleviate Pb‐induced lactate accumulation. A) The results of GSEA enrichment in the transcriptomics of carp liver. B) The content of crucial glucose metabolism‐related metabolites and enzymes (*n* = 6) by one‐way ANOVA. C) The related pathway of glucose metabolism. D) The enrichment of metabolites was analyzed by targeted metabolomics. E) The expression of glycolysis‐related genes in carp liver. F) Protein levels of glycolytic‐related genes in vitro. G) PAS staining of carp liver (Scale bar = 50 µm/20 µm in upper/lower panel). H) IHC of anti‐G6PD staining (Scale bar = 50 µm/20 µm in upper/lower panel). I) Molecular docking prediction of Mel targeting G6PD. J) CETSA results G6PD and Mel. K) CETSA results of G6PD in vitro. L) Statistic results of CETSA. M) Protein level of G6PD in the si G6PD models with Pb and/or Mel treatment. N) The content of G6PD in vitro models (*n* = 6) by one‐way ANOVA with Tukey's multiple comparison test. O) Lactate content in vitro (*n* = 6) by one‐way ANOVA.

During the assembly of Figure 4B, the protein bands were misaligned with their experimental groups. The corrected image 4(B) is provided below.


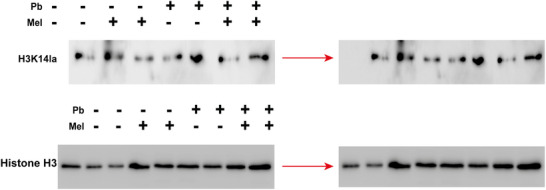


Figure 4 (B) The lactylation modification in vitro.

Corrected Figure 4 is provided below.


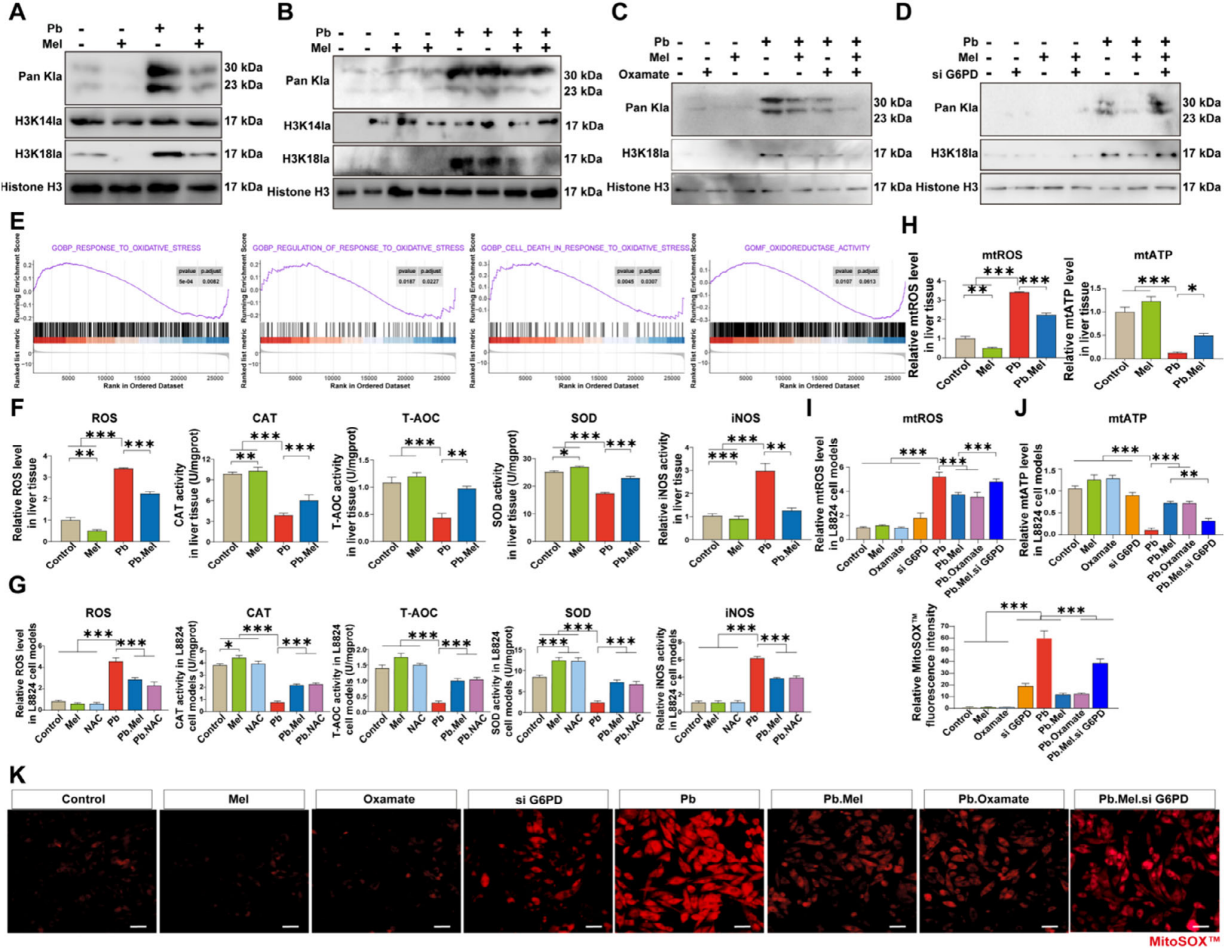


**Figure 4**. Mel improves Pb‐mediated mitochondrial oxidative stress by alleviating H3K18 lactylation. A) The lactylation modification of carp liver. B) The lactylation modification in vitro. C) The alter of lactylation modification in vitro models using Oxalate as glycolysis inhibitor. D) The lactylation modification changes in si G6PD models. E) Oxidative stress‐related enrichment of GSEA in the liver transcriptome of carp. F) The content of ROS and activities of the CAT, T‐AOC, SOD, iNOS in liver tissues (*n* = 6) by one‐way ANOVA with Tukey's multiple comparison test. G) the content of oxidative stress indicators (*n* = 6). I) Relative mtROS levels in cell models (*n* = 6). J) Relative mtATP levels in vitro (*n* = 6). K) MitoSOX Red staning of cell models (Scale bar = 20 µm).

The authors apologize for this error and for the inconvenience it may have caused.

