# Supplementary material for: Melatonin Modulates Glucose Metabolism Reprogramming via Targeting G6PD to Alleviate Lead‐Induced Hepatocytes Pyroptosis in Common Carp (Cyprinus carpio L.)
Source: Adv Sci (Weinh). 2025 Aug 11;12(41):e01041. doi: 10.1002/advs.202501041 (PMC12591103; doi:10.1002/advs.202501041)
Supplement: Supplementary file 1 — Supporting Information [file ADVS-12-e01041-s001.docx]

# Supplementary Tables

## **Table S1.** The screened literature.

| **Article Title** | **Continent** | **County, Regions** | **Sample** |
| --- | --- | --- | --- |
| Current situation of lead (Pb) exposure in raptors and waterfowl in Japan and difference in sensitivity to in vitro lead exposure among avian species | Asia | Japan | Waterfowl and Avian Species |
| Elevated lead (Pb) in urban European starling (*Sturnus vulgaris*) feathers is not correlated to physiology or behavior. | North America | Metro-Atlanta | European starlings (Sturnus vulgaris) |
| High precision blood lead radiogenic isotope signatures in a community exposed to Pb contaminated soils and implications for the current Pb exposure of the European population. | Europ | Liège (Belgium) | Soil and Drinking Water |
| Identification of copper and lead pollution elements based on spectra of corn leaves in different leaf layers. | Asia | Beijing Olympic Park | Soil and Plant |
| Lead and copper removal from sterile dumps by phytoremediation with Robinia pseudoacacia | Europ | Almașu Mare commune of Alba County (Romania) | Soil |
| Exposure to Sublethal Concentrations of Lead (Pb) Affects Ecologically Relevant Behaviors in House Sparrows (*Passer domesticus*) | North America | Newport News, Williamsburg, and York counties in Virginia, USA | Avian species |
| Ineffectiveness of phosphorus-containing amendments to reduce Pb bioaccessibility in an urban alkaline soil. | North America | Cleveland | Soil |
| Solidification/stabilization of lead-contaminated soil using alkali-activated volcanic ash | Asia | Tehran | Soil |
| A review of heavy metals pollution in riverine sediment from various Asian and European countries: Distribution, sources, and environmental risk. | Europ and Asia | Iran, Turkey, Spain, Vietnam, Pakistan, Malaysia, Taiwan, Chin, Nigeria, Bangladesh, Japan | Watershed |
| Interactions between blood lead (Pb) concentration, oxidative stress, cellular immune response and reproductive status in livestock from a mining area | Europ | Iberian Peninsula | Soil and Animal |
| Lead Speciation, Bioaccessibility, and Sources for a Contaminated Subset of House Dust and Soils Collected from Similar United States Residences. | North America | United States | House Dust and Soils |
| Trace metal partitioning in the parnaíba delta in dry season, equatorial coast of Brazil. | South America | Parnaiba River Delta, Brazil | Deposit Sediment |
| Trace elements and heavy metals in black vultures (*Coragyps atratus*) and Turkiye vultures (Cathartes aura) in the southeastern United States. | North America | Southeastern United States | Avian species Falconiformes |
| Geochemical elements in suspended particulate matter of Ensenada de La Paz Lagoon, Baja California Peninsula, Mexico: Sources, distribution, mass balance and ecotoxicological risks. | North America | Baja California Sur, Mexico | Suspended Particulate |
| Integrated environmental assessment of iron ore tailings in floodplain soils and plants after the Fundao Dam disaster in Brazil. | South America | Doce River Basin, Brazil | Soil |
| Effects of Lead (Pb) from Smelter Operations in an Urban Terrestrial Food Chain at a Colorado Superfund Site. | North America | Colorado Superfund Site,United States | Soil |
| Ecological risk assessment for metals in sediment and waters from the Brazilian Amazon region. | South America | Amazonas, Brazil | Deposit Sediment |
| Contamination of As, Cd, Cr, Hg and Pb in soils in Arica commune (Chile). | South America | Arica, Chile | Soil |
| Ecotoxicology applied to conservation: Potential negative metal and metalloid contamination effects on the homeostatic balance of the critically endangered Brazilian guitarfish, *Pseudobatos horkelii* . | South America | Southeastern Brazil | Guitarfish |
| Vertical distribution and trace element contamination in sediment cores affected by gold mining in Colombia. | South America | Atrato River, the Delicias Marsh and the Encaramada Marsh of Colombia | Deposit Sediment |
| Seasonal differences in trace metal concentrations in the major rivers of the hyper-arid southwestern Andes basins of Peru. | South America | Andes Basins, Peru | Water |
| Metal bioaccumulation and genotoxicity in *Oreochromis niloticus* reared in farming pools influenced by mining activities in Napo, in the Ecuadorian Amazonia. | South America | Northern Amazon, Ecuador | Fish |
| Sediment Spatial Distribution and Quality Assessment of Metals in Chinook Salmon and Resident Killer Whale Marine Habitat in British Columbia, Canada. | North America | Northeastern Pacific, Canada | Surface Sediments |
| An assessment of the relation between metal contaminated sediment and freshwater mussel populations in the Big River, Missouri. | North America | Big River in southeast Missouri, United States | Deposit Sediment |
| Heavy Metal Levels and Cancer Risk Assessments of the Commercial Denis, *Sparus aurata* Collected from Bardawil Lake and Private Fish Farm Waters as a Cultured Source, Egypt. | Africa | Bardawil Lake, Egypt | Fish |
| Assessing the spatial distribution of elemental concentrations in surface sediments of Lake Victoria, Kenya: implications for ecological health and management. | Africa | Victoria Lake, Kenya | Deposit Sediment |
| Distribution, speciation, and assessment of heavy metals in sediments from Wadi Asal, Red Sea, Egypt. | Africa | Wadi Asal, Red Sea, Egypt | Deposit Sediment |
| Assessing the ecological and health risks associated with heavy metal pollution levels in sediments of Big Giftun and Abu Minqar Islands, East Hurghada, Red Sea, Egypt. | Africa | Abu Minqar, Egypt | Deposit Sediment |
| Burial leakage: A human accustomed groundwater contaminant sources and health hazards study near cemeteries in Benin City, Nigeria. | Africa | Nigeria Benin | Water |
| Seasonal and spatial contamination of trace elements in sediments and fish tissues (*Mugil Chephalus*) from Annaba gulf (North East of Algeria). | Africa | Annaba Gulf, Algeria | Fish |
| Trace metal load of two urban wetlands with varied catchment activities in Ghana. | Africa | Vaughan Dam,Ghana | Deposit Sediment |
| Bioaccumulation, Bioindication and Health Risk Assessment of Heavy Metals in Cape Horse Mackerel (*Trachurus trachurus*) and Slinger Seabream (*Chrysoblephus puniceus*) in the Durban Basin and Cape Vidal, South Africa. | Africa | Cape Vidal, South Africa | Fish |
| Considerations for environmental biogeochemistry and food security for aquaculture around Lake Victoria, Kenya. | Africa | Victoria Lake, Kenya | Aquaculture and Wild Fish Species |
| Assessment of heavy metal contamination and ecological risk in Morocco's marine and estuarine ecosystems through a combined analysis of surface sediment and bioindicator species: Donax trunculus and Scrobicularia plana. | Africa | Agadir Bay, Southern Morocco | Deposit Sediment |
| Human health risks of metal contamination in Shallow Wells around waste dumpsites in Abeokuta Metropolis, Southwestern, Nigeria. | Africa | Abeokuta Metropolis, Nigeria | Water |
| Benthic foraminifera as bioindicators for the heavy metals in the severely polluted Hurghada Bay, Red Sea coast, Egypt. | Africa | Hurghada Bay, Red Sea Coast, Egypt | Aquatic Organisms |
| Source-specific probabilistic health risk assessment of heavy metals in surface water of the Yangtze River Basin. | Asia | China | Water |
| Interaction between Haematococcus pluvialis microalgae and lead nitrate: lead adsorption from water. | Asia | Tabriz, Iran | Water |
| Health risks and sources of trace elements and black carbon in PM2.5 from 2019 to 2021 in Beijing. | Asia | Beijing, China | Air |
| Impacts of solid waste management site on some toxic elements contamination of the surrounding soil in Akure, Nigeria. | Asia | 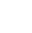Akure, Nigeria | Soil |
| Environmental pollution and human health risk due to tailings storage facilities in China. | Asia | China | Soil |
| The significance of lead entering the human food chain via livestock ingestion from the agricultural use of biosolids, with special reference to the UK. | Europ | United Kingdom | Soil |
| Assessment of Toxic Element Contamination in Honey, Milk, and Eggs from Algiers (Algeria) Using Inductively Coupled Plasma Mass Spectrometry (ICP-MS): Exploring Health Implications of Pollution. | Africa | Algiers, Algeria | Food |
| Biomonitoring and Biomathematical Modeling of Health Risks Associated with Dumpsite Grown Vegetables in Lagos State. | Asia | Lagos, Nigeria | Soil Plant |
| Prevalence rates of neurodegenerative diseases versus human exposures to heavy metals across the United States. | North America | United States | Infant Blood; Sewage Sludge |
| Association of the external environmental exposome and obesity: A comprehensive nationwide study in 2019 among Chinese children and adolescents. | Asia | China | Children and Adolescents |
| Lead exposure across the life course and age at death. | North America | United States | Human |
| Experiments and modeling to develop a Pistia stratiotes based Floating Vegetated System (FVS) for the removal of heavy metals (Pb, Zn, Cr, Cu, Ni). | Asia | Odisha, India | Water |
| Assessment of essential and potentially toxic metals in raw cow milk from Mukaturi town, Oromia Regional State, Ethiopia. | Africa | Oromia Region of Ethiopia | Food Milk |
| Integrated application of multiple indicators and geographic information system-based approaches for comprehensive assessment of environmental impacts of toxic metals-contaminated agricultural soils and vegetables. | Asia | Fars, Iran | Soils and Vegetables |
| Current situation of lead (Pb) exposure in raptors and waterfowl in Japan and difference in sensitivity to in vitro lead exposure among avian species. | Asia | Japan | Avian species |
| Deterioration phenomenon of Pb-contaminated aqueous solution remediation and enhancement mechanism of nano-hydroxyapatite-assisted biomineralization. | Asia | China | Water |
| Rapid and sensitive in situ detection of heavy metals in fish using enhanced Raman spectroscopy. | Africa | Egypt | Fish |
| Speciation, bioaccumulation, and toxicity of the newly deposited atmospheric heavy metals in soil-earthworm (*Eisenia fetida*) system near a large copper smelter. | Asia | Jiangxi, China | Earthworm |
| Analysis and Evaluation of Heavy Metal Pollution in Farmland Soil in China: A Meta-analysis | Asia | Yunnan, China | Soil |
| Bacterial Community Structure of Typical Lake Sediments in Yinchuan City and Its Response to Heavy Metals. | Asia | Ningxia, China | Water |
| Entrapment of atmospheric particle bound heavy metals by ferns as evidenced by lead (Pb) isotope and MixSIAR: Implications for improving air quality. | Asia | China | Air |
| Exposure risks of lead and other metals to humans: A consideration of specific size fraction and methodology. | Asia | Shaanxi, China | Food |
| Historical construction, quantitative source identification and risk assessment of heavy metals contamination in sediments from the Pearl River Estuary, South China. | Asia | Guangdong, China | Deposit Sediment |
| Compositional-geochemical characterization of lead (Pb) anomalies and Pb-induced human health risk in urban topsoil. | Asia | Yerevan, United States | Soil |
| Bioaccumulation and health risk assessment of trace elements in Tilapia (*Oreochromis mossambicus*) from selected inland water bodies. | Asia | Tamil Nadu, India | Fish |
| Risk assessment of four toxic heavy metals in terrestrial and aquatic ecosystems around BSCIC tannery industrial estate of Savar, Dhaka, Bangladesh. | Asia | Dhaka, Bangladesh | Water |
| Accumulation of Cd and Pb in sediments and Asian swamp eels (*Monopterus albus*) from downstream area of Cisadane River, Indonesia. | Asia | Indonesia | Water |
| Incidence of microplastic contamination in fishes of the Ramsar Wetland, Loktak - The world's only floating lake from the Indian Himalayan region. | Asia | Himalayan, India | Fish |
| Integrated assessment of potentially toxic elements in soil of the Kangdian metallogenic province: A two-point machine learning approach. | Asia | China | Soil |
| On the electrokinetic remediation of Pb-contaminated soil: A coupled electro-transport-reaction modelling study based on chemical reaction kinetics. | Asia | China | Soil |
| Estimated Childhood Lead Exposure From Drinking Water in Chicago. | North America | United States | Water |
| Effects of land use/cover change on heavy metal distribution of soils in wetlands and ecological risk assessment. | Asia | Turkiye | Soil |
| Tolerance of the Australian halophyte, beaded samphire, Sarcocornia quinqueflora, to Pb and Zn under glasshouse conditions: Evaluating metal uptake and partitioning, photosynthetic performance, biomass, and growth. | Oceania | Australia | Soil Plant |
| Biotransformation of Pb and As from sewage sludge and food waste by black soldier fly larvae: Migration mechanism of bacterial community and metalloregulatory protein scales. | Asia | Hubei, China | Food |
| Assessment of Microplastics and Potentially Toxic Elements in Surface Sediments of the River Kelvin, Central Scotland, United Kingdom. | Europ | United Kingdom | Deposit Sediment |
| Geochemical evaluation, ecological and human health risk assessment of potentially toxic elements in urban soil, Southern India. | Asia | Chennai, India | Soil |
| Temporal changes in metal and arsenic concentrations in blood and feathers of tropical seabirds after one of the largest environmental disasters associated with mining. | Oceania | Australia | Avian species |
| Intelligent monitoring of the available lead (Pb) and cadmium (Cd) in soil samples based on half adder and half subtractor molecular logic gates. | Asia | Guangzhou, China | Soil |
| Neurological risks arising from the bioaccumulation of heavy metal contaminants: A focus on mercury. | Asia | China | Water and Sediments |
| Lead, Cadmium, and Arsenic in Raw Milk Produced in the Vicinity of a Mini Mineral Concentrator in the Central Andes and Health Risk. | South America | Peru | Food |
| Lactational Exposure of Human Infants to Metal (loid)s: A Comparison of Industrial and Urban Inhabitants in North of the Persian Gulf. | Asia | Bushehr, Iran | Human |
| Lead and copper removal from sterile dumps by phytoremediation with *Robinia pseudoacacia*. | Europ | Romania | Sterile Dump |
| Bioremediation of Pb contaminated water using a novel Bacillus sp. strain MHSD_36 isolated from *Solanum nigrum*. | Africa | South Africa | Water |
| Occurrence of toxic elements in river areas along drains and groundwater resources: source of contamination and associated health risk. | Asia | Britannica, India | Water |
| Pb isotopic fingerprinting of uranium pollution: New insight on uranium transport in stream-river sediments. | Asia | Guangdong, China | Deposit Sediment |
| Driving factors for distribution and transformation of heavy metals speciation in a zinc smelting site. | Asia | Hunan, China | Soil |
| Heavy metals bioaccumulation in free-ranging South American rattlesnakes (*Crotalus durissus*) in Southeastern Brazil. | South America | Brazil | Reptilia species |
| Co-pyrolysis of alkali-fused fly ash and corn stover to synthesize biochar composites for remediating lead-contaminated soil. | Asia | Shanxi, China | Soil |
| Metal accumulation in salt marsh soils along the East Coast of the United States. | North America | Maine, United States | Soil |
| Comprehensive Elemental Profiling of Romanian Honey: Exploring Regional Variance, Honey Types, and Analyzed Metals for Sustainable Apicultural and Environmental Practices. | Europ | Romania | Food |
| Soil pollution indices and health risk assessment of metal(loid)s in the agricultural soil of pistachio orchards. | Asia | Khorasan Razavi, Iran | Soil |
| Assessment of bioremediation potential of *Calotropis procera* and *Nerium oleander* for sustainable management of vehicular released metals in roadside soils. | Asia | Lahore, Pakistan | Soil |
| Assessment of heavy metals level in chicken with indeterminate analysis in localities of Lahore, Pakistan. | Asia | Lahore, Pakistan | Avian species |
| Heavy metals in water and sediment of Cikijing River, Rancaekek District, West Java: Contamination distribution and ecological risk assessment. | Asia | Indonesia | Deposit Sediment |
| Growth-dependent cr(VI) reduction by Alteromonas sp. ORB2 under haloalkaline conditions: toxicity, removal mechanism and effect of heavy metals. | Asia | Kalpakkam, India | Soil |
| Evaluation of potentially toxic elements in soils developed on limestone and lead-zinc mine sites in parts of southeastern Nigeria. | Africa | Nigeria | Soil |
| Association between exposure to heavy metals in atmospheric particulate matter and sleep quality: A nationwide data linkage study. | Asia | Seoul, South Korea | Air |
| Contaminant transport from solid waste dumps: Implications for environmental degradation with a focus on capital territory regions. | Asia | Islamabad & Lohsar, Pakistan | Water |
| Evaluation of soil pollution by heavy metal using index calculations and multivariate statistical analysis. | Europ | Aksaray, Turkiye | Soil |
| Homemade weaning foods as a source of lead and mercury exposure in Korean infants - A dietary risk assessment study. | Asia | Seoul, South Korea | Food |
| Heavy metal contamination and environmental risk assessment: a case study of surface water in the Bahr Mouse stream, East Nile Delta, Egypt. | Africa | East Nile Delta, Egyp | Water |
| Prediction of heavy metal lead contamination accident in Three Gorges Reservoir Area. | Asia | Sichuan, China | Water |
| Exposure to Sublethal Concentrations of Lead (Pb) Affects Ecologically Relevant Behaviors in House Sparrows (*Passer domesticus*). | North America | California, United States | Avian species |
| The longevity evaluation of multi-metal stabilization by MgO in Pb/Zn smelter-contaminated soils. | Asia | China | Soil |
| Occurrence and toxicological relevance of pesticides and trace metals in agricultural soils, sediments, and water of the Sogamoso River basin, Colombia. | South America | Colombia | Water |
| Chemical speciation and comprehensive risk assessment of metals in sediments from Nabq protectorate, the Red Sea using individual and synergistic indices. | Africa | Egypt | Deposit Sediment |
| Potentially toxic elements fluxes in (210)Pb-dated sediment cores from a large coastal lagoon (southern Gulf of Mexico) under environmental stress. | North America | Mexico | Deposit Sediment |
| Airborne (210)Pb, Si, Zn and Pb as tracers for atmospheric pollution in Helsinki metropolitan area. | Europ | Helsinki, Finland | Air |
| Blood lead, cadmium and hair mercury concentrations and association with soil, dust and occupational factors in e-waste recycling workers in Bangladesh. | Asia | Bangladesh | Soil,Dust |
| Assessment of heavy metal accumulation and potential risks in surface sediment of estuary area: A case study of Dagu river. | Asia | Shandong, China | Water |
| Effect of peanut shell and wheat straw biochar on the availability of Cd and Pb in a soil-rice (*Oryza sativa* L.) system | Asia | Hunan, China | Soil |
| Effects of combined amendments on heavy metal accumulation in rice (*Oryza sativa* L.) planted on contaminated paddy soil | Asia | Hunan, China | Soil |
| Mitigation effects of silicon rich amendments on heavy metal accumulation in rice (*Oryza sativa* L.) planted on multi-metal contaminated acidic soil | Asia | Guangdong, China | Soil |
| Variations in heavy metal accumulation, growth and yield of rice plants grown at different sewage sludge amendment rates | Asia | Uttar Pradesh, India | Soil |
| Remediation of Cd, Pb and as Co-contaminated Paddy Soil by Applying Different Amendments | Asia | Guangxi, China | Soil |
| Size-resolved distribution of trace elements in lysimeter soil solutions under contrasting long-term agricultural management to assess their bioavailability. | North America | Edmonton, Canada | Soil |
| Health Risk Assessment of Heavy Metals in Soils of a City in Guangdong Province Based on Source Oriented and Monte Carlo Models | Asia | Guangzhou, China | Soil |
| Differentiating anthropogenic effects from natural metal(loid) levels in residential soil near a zinc smelter in South Korea. | Asia | Yuseong-Gu, South Korea | Soil |
| Effect of biochar, zeolite and bentonite on physiological and biochemical parameters and lead and zinc uptake by maize (*Zea mays* L.) plants grown in contaminated soil. | Asia | Bahawalpur, Pakistan | Soil |
| Biochar and nano-hydroxyapatite combined remediation of soil surrounding tailings area: Multi-metal(loid)s fixation and soybean rhizosphere soil microbial improvement. | Asia | Beijing, China | Soil |
| Effects of nitrogen regulation on heavy metal phytoextraction efficiency (*Leucaena leucocephala*): application of a nitrogen fertilizer and a fungal agent. | North America | Ontario, Canada | Soil |
| The effect of soil types, pH, and geographical locations on carcinogenic metal buildup in *Oryza sativa* cultivated in Ghana. | Africa | Ghana | Soil |
| Removal of toxic metals from sewage sludge by EDTA and hydrodynamic cavitation and use of the sludge as fertilizer. | Europ | Ljubljana, Slovenia | Sewage Sludge |
| Relation between solid phase speciation and oral/lung bioaccessibility of metal(loid)s polluted soils in inhabited area: Contribution of synchrotron-based experiment. | Europ | Viviez, France | Soil |
| A new simple index for characterizing the labile heavy metal concentration in soil by diffusive gradients in thin films technique. | Asia | Beijing, China | Soil |
| The formation of multi-metal(loid)s contaminated groundwater at smelting site: Critical role of natural colloids. | Asia | Nanjing, China | Water |
| Comprehensive assessment of the microbial community structure in a typical lead-zinc mine soil. | Asia | Hunan, China | Soil |
| The Impact of Inoculation of Two Strains of Rhizobacteria on Radionuclide Transfer in Sesbania Grandiflora. | Asia | Damascus, Syria | Soil |
| Errors in agricultural practices increase the toxicity of heavy metals in the food chain at Ishwardi Upazila in Bangladesh. | Asia | Rajshahi, Bangladesh | Soil |
| Heavy metals drive microbial community assembly process in farmland with long-term biosolids application. | Asia | Beijing, China | Soil |
| Wide Riparian Zones Inhibited Trace Element Loss in Mining Wastelands by Reducing Surface Runoff and Trace Elements in Sediment. | Asia | Yunan, China | Deposit Sediment |
| Neighborhood-scale lead (Pb) speciation in Akron, Ohio (USA) soils: primary sources, post-deposition diagenesis, and high concentrations of labile Pb. | North America | Ohio, United States | Soil |
| Health risk assessment of heavy metals in some vegetables-Erbil City-Kurdistan Region of Iraq. | Asia | Kurdistan, Iraq | Soil |
| Health and ecological risk of heavy metals in agricultural soils related to Tungsten mining in Southern Jiangxi Province, China. | Asia | Jiangxi, China | Soil |
| Maintaining the cultivation of vegetables with low Pb accumulation while remediating the soil of an allotment garden (Nantes, France) by phytoextraction. | Europ | Nantes, France | Soil |
| Ecological risk assessment of heavy metals in desulfurized seawater discharged from a coal-fired power plant in Qingdao. | Asia | Shandong, China | Seawater |
| Role of the molecular structure of humified organic matter in rice plant response to environmental lead pollution. | South America | Seropedica, Brazil | Soil |
| Influence of conditioner and straw on the herbaceous plant-based phytoremediation copper tailings: a field trial at Liujiagou tailings pond, China. | Asia | Jiangxi, China | Soil |
| Biogenic manganese oxides promote metal(loid) remediation by shaping microbial communities in biological aqua crust. | Asia | Guangzhou Tailing Area of Meizhou, China | Water |
| Mechanism and effectiveness of enzymatically induced phosphate precipitation (EIPP) in stabilizing coexisting lead, zinc, and cadmium in tailings. | Asia | Sichuan Tailing Area of Miyi, China | Water |
| Mineral and potentially toxic element profiles in the soil-feed-animal continuum: Implications for public, environmental, and livestock health in three pasture-based sheep farming systems. | Europ | Caceres, Spain | Soil |
| Lead exposure and its relationship with fecal cortisol levels in black howler monkeys (*Alouatta pigra*). | North America | Mexico | Soil and animal |
| United States house dust Pb concentrations are influenced by soil, paint, and house age: insights from a national survey. | North America | United States | Soil |
| Phytostabilization of metal(loid)s by ten emergent macrophytes following a 90-day exposure to industrially contaminated groundwater. | Europ | Burgos, Spain | Water |
| Organic Materials Promote *Rhododendron simsii* Growth and Rhizosphere Soil Properties in a Lead-Zinc Mining Wasteland. | Asia | Yunnan Tailing Area, China | Soil |
| Evaluation of heavy metal speciation distribution in soil and the accumulation characteristics in wild plants: A study on naturally aged abandoned farmland adjacent to tailings. | Asia | Gansu Baiyin, China | Soil |
| Integrated assessment of the pollution and risk of heavy metals in soils near chemical industry parks along the middle Yangtze River. | Asia | Industrial Parks along the Middle Yangtze River, China | Soil |
| Assessment of potential ecological risk of heavy metal contamination of agricultural soils in Kazakhstan. | Asia | Almaty, Kazakhstan | Soil |
| Effect and potential mechanisms of sludge-derived chromium, nickel, and lead on soil nitrification: Implications for sustainable land utilization of digested sludge. | Asia | Hongxing village in Harbin, China | Soil |
| Health risk assessment of heavy metals based on source analysis and Monte Carlo in the downstream basin of the Zishui. | Europ | Monte Carlo, Monaco | Water |
| Human health risk assessment of metal-contaminated soils in Sydney estuary catchment (Australia) | Oceania | Sydney, Australia | Catchment Soil |
| Characteristics, source analysis, and health risk assessment of potentially toxic elements pollution in soil of dense molybdenum tailing ponds area in central China. | Asia | East Qinling, China | Soil |
| Microecological characteristics of water bodies/sediments and microbial remediation strategies after 50 years of pollution exposure in ammunition destruction sites in China. | Asia | Ammunition Destruction Sites in Jilin, China | Water and Sediments |
| Effect of biological sewage sludge and its derived biochar on accumulation of potentially toxic elements by corn (*Zea mays* L.). | Asia | Chaharmhal-Bakhtiari, Iran | Sewage Sludge |
| Intercropping of *Pteris vittata* and maize on multimetal contaminated soil can achieve remediation and safe agricultural production. | Asia | Beijing and Shaanxi, China | Soil |
| Blocking Effects of Foliar Conditioners on Cadmium, Arsenic, and Lead Accumulation in Wheat Grain in Compound-contaminated Farmland. | Asia | Hebei, China | Soil |
| Characteristics and Identification Priority Source of Heavy Metals Pollution in Farmland Soils in the Yellow River Basin. | Asia | Yellow River Basin, China | Soil |
| Phytoremediation of pollutants in oil-contaminated soils by *Alhagi camelorum*: evaluation and modeling. | Asia | Gachsaran Oil Field, Iran | Soil |
| Health risk assessment and potential sources of metals in riparian soils of the Wujiang River, China. | Asia | Wujiang River Basin, China | Soil |
| Bioaccessibility of lead and cadmium in soils around typical lead-acid power plants and their effect on gut microorganisms. | Asia | Jinan, China | Soil |
| Composition characteristics and metal binding behavior of macrophyte-derived DOM (MDOM) under microbial combined photodegradation: A state closer to actual macrophytic lakes. | Asia | Jinan, China | Water |
| Organic amendment application affects the release behaviour, bioavailability, and speciation of heavy metals in zinc smelting slag: Insight into dissolved organic matter. | Asia | Shandong Dongping Lake, China | Water |
| The impact of recycling polyaluminium chloride and anionic polyacrylamide water treatment residuals on heavy metal adsorption in soils: implications for stormwater bioretention systems. | Asia | Shanxi, China | Water |
| Comparison of organic and synthetic amendments for poplar phytomanagement in copper and lead-contaminated calcareous soil. | Asia | Jinan, China | Soil |
| Mineralogical and sorption characterization of lateritic soils from Southwestern Nigeria for use as landfill liners. | Asia | Baiyin Sewage Irrigation Area, China | Soil |
| Impact of lead and zinc heavy metal pollution on the growth and phytoremediation potential of Sulla carnosa in Sebkha el Kalbia, Tunisia. | Africa | Sebkha el Kalbia, Tunisia | Soil |
| Alginate-encapsulated biochar as an effective soil ameliorant for reducing Pb phytoavailability to lettuce (*Lactuca sativa* L.). | Asia | Chuncheon, South Korea | Soil |
| Application of thermally treated sludge residues on an e-waste contaminated soil: effects on PTE bioavailability, soil physicochemical and biological properties, and *L. perenne* growth. | Asia | Hangzhou, China | Sewage Sludge |
| Post-sorption of Cd, Pb, and Zn onto peat, compost, and biochar: Short-term effects of ecotoxicity and bioaccessibility. | South America | Registro, Brazil | Soil |
| Ecological and human health hazards of soil heavy metals after wildfire: A case study of Liangshan Yi autonomous prefecture, China. | Asia | Sichuan, China | Soil |
| Flooding regimes alleviate lead toxicity and enhance phytostabilization of *salix*: Evidence from physiological responses and iron-plaque formation. | Asia | Guangzhou Local Wetland China | Soil |
| Synergistic effect of pyrene and heavy metals (Zn, Pb, and Cd) on phytoremediation potential of *Medicago sativa* L. (alfalfa) in multi-contaminated soil. | Asia | Rajasthan, India | Soil |
| Discrimination of metal contaminant sources in river sediments influenced by mining and smelting activities using stable Pb and Zn isotopes. | Asia | Daejeon, South Korea | Deposit Sediment |
| Can blood morphology, oxidative stress, and cholinesterase activity determine health status of pigeon *Columba livia f. urbana*? | Europ | Slupsk, Poland | Avian species |
| Alleviating the adverse effects of Cd-Pb contamination through the application of silicon fertilizer: Enhancing soil microbial diversity and mitigating heavy metal contamination. | Asia | Yunnan, China | Soil |
| Trace element accumulation behavior, ability, and propensity of *Taraxacum officinale* F.H. Wigg (Dandelion). | Asia | Ankara, Turkiye | Soil |
| Urban green space area mitigates the accumulation of heavy metals in urban soils. | Asia | Shanghai, China | Soil |
| Effect of montmorillonite modified straw biochar on transfer behavior of lead and copper in the historical mining areas of dry-hot valleys. | Asia | Yunnan-Guizhou Plateau and Panxi Plateau, China | Soil |
| Pine needles as bioindicator and biomagnetic indicator of selected metals in the street dust, a case study from southeastern Iran. | Asia | Kerman, Iran | Dust |
| Phytoremediation and environmental effects of three Amaranthaceae plants in contaminated soil under intercropping systems. | Asia | Changsha, China | Soil |
| Metal pollution drives earthworm biodiversity in urban lawns. | Europ | Paris, France | Soil |
| The leaching behavior of heavy metal from contaminated mining soil: The effect of rainfall conditions and the impact on surrounding agricultural lands. | Asia | Yaoposhan polymetallic mining area and the downstream village Xuya, China | Soil |
| Heavy metals in onion (*Allium cepa* L.) and environmental and health risks. | Europ | Nitra, Slovakia | Soil |
| Exploring geochemical distribution of potentially toxic elements (PTEs) in wetland and agricultural soils and associated health risks. | Asia | Telangana, India | Soil |
| Evaluation of the Effects of Wastewater Irrigation on Heavy Metal Accumulation in Vegetables and Human Health in the Cauliflower Example : Heavy Metal Accumulation in Cauliflower. | Asia | Usak, Turkiye | Wastewater, Soil and Plant |
| Assessment of heavy metals accumulation by vegetables irrigated with different stages of textile wastewater for evaluation of food and health risk. | Asia | Bangladesh | Wastewater, Soil and Plant |
| Effects of three plant growth-promoting bacterial symbiosis with ryegrass for remediation of Cd, Pb, and Zn soil in a mining area. | Asia | Dabao Mountain Mining Area, China | Soil and Plant |
| A comprehensive investigation of geoenvironmental pollution and health effects from municipal solid waste landfills. | Europ | Polish, Czech | Waste Landfill |
| Plant testing with hemp and miscanthus to assess phytomanagement options including biostimulants and mycorrhizae on a metal-contaminated soil to provide biomass for sustainable biofuel production. | Europ | Metaleurop Nord Pb/Zn Smelter, France | Soil |
| Source apportionment and source-specific risk assessment of bioavailable metals in river sediments of an anthropogenically influenced watershed in China. | Asia | China | Deposit Sediment |
| Road dust exposure and human corneal damage in a plateau high geological background provincial capital city: Spatial distribution, sources, bioaccessibility, and cytotoxicity of dust heavy metals. | Asia | Kunming, China | Soil and Dust |
| Elevated lead (Pb) in urban European starling (*Sturnus vulgaris*) feathers is not correlated to physiology or behavior. | North America | Ontario, Canada | Avian species |
| Health risk assessment and quality reference values of potentially toxic elements in soils of the Southwestern Amazonas State - Brazil. | South America | Amazonas State, Brazil | Soil |
| Effects of elevation and geomorphology on cadmium, lead and chromium enrichment in paddy soil and rice: A case study in the Xiangtan basin of China. | Asia | Xiangtan Basin, China | Soil |
| Critical evaluation of the performance of rhamnolipids as surfactants for (phyto)extraction of Cd, Cu, Fe, Pb and Zn from copper smelter-affected soil. | Europ | Copper Smelter in Glogow, Poland | Soil |
| Screening of heavy metal-resistant rhizobial and non-rhizobial microflora isolated from *Trifolium* sp. growing in mining areas. | Africa | Mining Areas, Algeria | Soil |
| Ethnobotanical, ecological and health risk assessment of some selected wild medicinal plants collected along mafic and Ultra Mafic rocks of Northwest Pakistan. | Asia | Mafic and Ultramafic Regions, Pakistan | Soil and Plant |
| Spatial distribution of Pb and Zn in soils under native vegetation in Southeast Brazil. | South America | Minas Gerais, Brazil | Soil |
| Potentially toxic elements contamination in the water resources: an integrated risk assessment approach in the upper Citarum watershed area. | Asia | Citarum Watershed of West Java, Indonesia | Water |
| Enhancing Maize Yield and Soil Health through the Residual Impact of Nanomaterials in Contaminated Soils to Sustain Food. | Africa | Kafr El-Zayat, Egypt | Soil |
| Metal pollution in the topsoil of lands adjacent to Sahiwal Coal Fired Power Plant (SCFPP) in Sahiwal, Pakistan. | Asia | Sahiwal, Pakistan | Soil |
| Cadmium and lead accumulation in important food crops due to wastewater irrigation: Pollution index and health risks assessment. | Asia | Faisalabad and Multan, Pakistan | Wastewater, Soil and Plant |
| Total contents, fractionation and bioaccessibility of nine heavy metals in household dust from 14 cities in China. | Asia | China | Dust |
| Uncovering the impact of mega-scale shipbreaking yards on soil and crop quality in Bangladesh: A spatiotemporal dynamics and associated health risks of metal/loid contamination. | Asia | Chittagong, Bangladesh | Soil and Plant |
| In vitro bioaccessibility round robin testing for arsenic and lead in standard reference materials and soil samples. | North America | British Columbia, Canada | Soil |
| Metals and arsenic distribution in stray dogs' tissues around a lead-zinc mine in Kabwe, Zambia. | Africa | Kabwe, Zambia | Stray Dogs |
| Accumulation of copper and lead in ruminants grazing on a contaminated shooting range in Nordland County, Norway. | Europ | Nordland County, Norway | Ruminants |
| Human health risk assessment of metals in soil samples of a Brazilian city with a historic contamination complex. | South America | Rio Grande, Brazil | Soil |
| The impact of olive mill wastewater on soil properties, nutrient and heavy metal availability - A study case from Syrian vertisols. | Asia | Damascus, Syrian | Soil and Plant |
| Groundwater for drinking and industrial purposes: A study of water stability and human health risk assessment from black sand mineral rich coastal region of Kerala, India. | Asia | Kerala, India | Water |
| Honey Bees and Associated Matrices as Biomonitors of Soil Trace Elements: Assessment of their Sensitivity in a Regional Rural Environment. | Europ | Cesky les Protected Landscape Area, Czech | Honey Bees |
| Effect of metal fractions on rice grain metal uptake and biological parameters in mica mines waste contaminated soils. | Asia | Koderma and Giridih, India | Soil and Plant |
| Microbes-assisted phytoremediation of lead and petroleum hydrocarbons contaminated water by water hyacinth. | Asia | Faisalabad, Pakistan | Wastewater |
| Exposure and Health Risks Posed by Potentially Toxic Elements in Soils of Metal Fabrication Workshops in Mbarara City, Uganda. | Africa | Mbarara, Uganda | Soil |
| Impacts of long-term irrigation with coalmine effluent contaminated water on trace metal contamination of topsoil and potato tubers in Dinajpur area, Bangladesh. | Asia | Dinajpur, Bangladesh | Soil and Plant |
| The environmental impact of heavy metals in sediments of main valleys in the eastern side of Mosul City, Iraq. | Asia | Mosul, Iraq | Deposit Sediment |
| Heavy metals contamination and ecological risks in agricultural soils of Uşak, western Türkiye: a geostatistical and multivariate analysis. | Asia | Usak, Turkiye | Soil |
| Contamination level, spatial distribution, and sources of potentially toxic elements in indoor settled household dusts in Tehran, Iran. | Asia | Tehran, Iran | Dust |
| Risk assessment and source identification of soil heavy metals: a case study of farmland soil along a river in the southeast of a mining area in Southwest China. | Asia | Yunnan, China | Soil |
| Multiple potentially toxic elements in urban gardens from a Brazilian industrialized city. | South America | Santo Andre, Brazil | Soil |
| Contamination and health risks brought by arsenic, lead and cadmium in a water-soil-plant system nearby a non-ferrous metal mining area. | Asia | Guangxi Nandan Pb-Zn Mining Area, China | Soil |
| Groundwater heavy metal(loid)s risk prediction based on topsoil contamination and aquifer vulnerability at a zinc smelting site. | Asia | Zinc Smelting Site, China | Water |
| Geochemical characteristics and health risks of heavy metals in agricultural soils and crops from a coal mining area in Anhui province, China. | Asia | Anhui, China | Soil and Plant |
| Remediation options to reduce bioaccessible and bioavailable lead and arsenic at a smelter impacted site - consideration of treatment efficacy. | South America | Adelaide, Australia | Soil |
| Transfer of heavy metals from soil to tea and the potential human health risk in a regional high geochemical background area in southwest China. | Asia | Fengqing, Linxiang, Yongde, Mangshi, Longling, and Yunlong of Yunnan, China | Soil and Plant |
| Characterization of soil trace metal pollution, source identification, and health risk assessment in the middle reaches of the Guihe River Basin. | Asia | Guihe River Basin, China | Water |
| Influence of brassinosteroid and silicon on growth, antioxidant enzymes, and metal uptake of leafy vegetables under wastewater irrigation. | Asia | Sargodha, Pakistan | Wastewater, Soil and Plant |
| Spatio-temporal variability of public water supply characteristics and associated health hazards for children and adults in selected locations of Ambala, India. | Asia | Ambala, India | Water |
| Synergistic effect from combined use of scrap-recycling slag and hydrated lime to stabilize Pb and Zn in highly contaminated soil. | Asia | Wonju, South Korea | Soil |
| Bioaccumulation and sources of metal(loid)s in fish species from a subtropical river in Bangladesh: a public health concern. | Asia | Rupsha River Basin, Bangladesh | Water and Fish Species |
| Evaluating the capacity of heavy metal pollution enrichment in green vegetation in the industrial zone, Northwest China. | Asia | Xinjiang, China | Soil and Plant |
| Tracing the footprints of Arctic pollution: Spatial variations in toxic and essential elements in Svalbard reindeer (Rangifer tarandus platyrhynchus) faeces. | Europ | Nordenskiold Coast, Norway | Faeces |
| Heavy elements in indoor dust from Serbian households: pollution status, sources, and potential health risks. | Europ | Vojvodina, Serbia | Dust |
| Ecological and health risk assessment of heavy metals in agricultural soils from northern China. | Asia | Baiyin Mining Areas, China | Soil |
| Assessment of heavy metal distribution and bioaccumulation in soil and plants near coal mining areas: implications for environmental pollution and health risks. | Asia | Peshawar, Pakistan | Soil and Plant |
| Characteristics of soil contamination by potentially toxic elements in mine areas of Mongolia. | Asia | Mongolia | Soil |
| Ecotoxicity of heavy metals in soil around long-term e-waste recycling sites in Tema and Ashaiman areas of Ghana. | Africa | Tema and Ashaiman, Ghana | Soil |
| Soil heavy metal source apportionment and environmental differentiation study in Dulan County, Qinghai Province, using geodetector analysis. | Asia | Qinghai, China | Soil |
| Human activities contributing to the accumulation of high-risk trace metal(loid)s in soils of China's five major urban agglomerations. | Asia | Beijing, Tianjin, Hebei, Chengdu, Chongqing, Yangtze River Delta, Pearl River Delta of China | Soil |
| Chronic drought alters extractable concentrations of mineral elements in Mediterranean forest soils. | Europ | Natural Holm Oak Forest in the Prades Mountains in Catalonia, Spain | Soil |
| Analysis and pollution evaluation of heavy metal content in soil of the Yellow River Wetland Reserve in Henan. | Asia | Henan, China | Deposit Sediment |
| Response of Carrot (*Daucus carota* L.) to Multi-Contaminated Soil from Historic Mining and Smelting Activities. | Europ | Prague, Czech | Soil |
| Heavy metal contamination and health risk assessment of horticultural crops in two sub-cities of Addis Ababa, Ethiopia. | Africa | Addis Ababa, Ethiopia | Soil |
| Metal phytostabilization by mastic shrub (*Pistacia lentiscus* L.) and its root-associated bacteria in different habitats of Sardinian abandoned mining areas (Italy). | Europ | Italy | Soil |
| Contamination and ecological risk assessment of Cr, As, Cd and Pb in water and sediment of the southeastern Bay of Bengal coast in a developing country. | Asia | Eastern Bay of Bengal Coast, Bangladesh | Deposit Sediment |
| Seagrass soils sequester up to half the metal emissions of one of the world's largest smelters. | South America | Germein Bay on the Northeastern Side of the Spencer Gulf, Australia | Soil |
| Distribution and in-vitro bioaccessibility of potentially toxic metals in surface soils from a mining and a non-mining community in Ghana: implications for human health. | Africa | Kenyasi, Ghana | Soil |
| Toxic elements pollution risk as affected by various input sources in soils of greenhouses, kiwifruit orchards, cereal fields, and forest/grassland. | Asia | Shaanxi, China | Soil and Plant |
| Source-specific probabilistic risk evaluation of potentially toxic metal(loid)s in fine dust of college campuses based on positive matrix factorization and Monte Carlo simulation. | Asia | Xian, China | Dust |
| Association between heavy metal uptake and growth and reproduction in the anecic earthworm, *Alma nilotica* (Grube 1855). | Africa | Dschang, Cameroon | Soil |
| Field study of irrigation strategies with treated wastewater and saline water on heavy metal accumulation in barley grain. | Asia | Birjand, Iran | Soil |
| Assessment of the Wanyu River (China) based on a water, sediment and hydrobiont framework. | Asia | Wanyu River Basin, China | Water and Sediments |
| Evaluating trace elements in urban forest soils across three contrasting New England USA towns and cities by pXRF and mass spectrometry. | North America | Hartford, Lexington MA, Springfield MA of New England, United States | Soil |
| Distribution of Inorganic Contaminants Along the Coast of Ciudad de la Costa, Uruguay. | South America | Ciudad de la Costa, Uruguay | Water and Sand |
| Changes of heavy metal concentrations in farmland soils affected by non-ferrous metal smelting in China: A meta-analysis. | Asia | Smelting Areas, China | Soil |
| Ecotoxicological risks of metals in the subsistence food garden soils of Watut River floodplains, Papua New Guinea. | Oceania | New Guinea, Papua | Soil |
| A circular economy approach to drinking water treatment residue management in a catchment impacted by historic metal mines. | Europ | Whiteheaps Mine Area, United Kingdom | Soil and Water |
| Contamination, ecological-health risks, and sources of potentially toxic elements in road-dust sediments and soils of the largest urban riverfront scenic park in China. | Asia | Yellow River Basin, China | Soil,Water, Sediment |
| Heavy metals pollution of soil in central plains urban agglomeration (CPUA), China: human health risk assessment based on Monte Carlo simulation. | Asia | Hainan, China | Soil |
| Dietary sources apportionment and health risk assessment for trace elements among residents of the Tethys-Himalayan tectonic domain in Tibet, China. | Asia | Tibet, China | Soil and Plant |
| A predictive assessment of the uranium ore tailings impact on surface water contamination: Case study of the city of Kamianske, Ukraine. | Europ | Kamianske Dnipro River Basin, Ukraine | Water |
| Evaluating natural and anthropogenic inputs on the distribution of potentially toxic elements in urban soil of Valdivia, Chile. | South America | Valdivia, Chile | Soil |
| Metal bioaccumulation in spontaneously grown aquatic macrophytes in Fe-rich substrates of a passive treatment plant for acid mine drainage. | Europ | Huelva, Spain | Water and Plant |
| New method for risk assessment in environmental health: The paradigm of heavy metals in honey. | Europ | Bucharest around Industral Areas, RomaniA | Food Linden, Rapeseed and Polyfloral Honey |
| Landfill leachate has multiple negative impacts on soil health indicators in Hyrcanian forest, northern Iran. | Asia | Hyrcanian Forest, Iran | Soil |
| Variations in elemental composition of rice (*Oryza sativa* L.) with different cultivation areas of Ethiopia. | Africa | Gondar, Ethiopia | Soil |
| Impacts of the steel industry on sediment pollution by heavy metals in urban water system. | Asia | Maanshan Iron Mine Zone, China | Soil and Sediment |
| Urinary concentrations of heavy metals in pregnant women living near a petrochemical area according to the industrial activity. | Europ | Tarragona, Spain | Human Urine and Liver |
| Geochemical fractionation, bioaccessibility and ecological risk of metallic elements in the weathering profiles of typical skarn-type copper tailings from Tongling, China. | Asia | Tongling, China | Soil |
| Health Risk Assessment of Heavy Metals in Agricultural Soils Around the Gangue Heap of Coal Mine Based on Monte Carlo Simulation | Asia | Zhejiang, China | Soil |
| Engineered DNA molecular machine for ultrasensitive detection of environmental lead pollution. | Asia | Chaohu Lake (Water, Soil and Fish)and Bohai Sea, China | Water and Fish Species |
| Assessment of atmospheric heavy metal pollution in Qinghai-Tibet Plateau: Using mosses as biomonitor. | Asia | Qinghai-Tibet Plateau, China | Soil and Plant |
| Contamination, ecological, and human health risks of heavy metals in water from a Pb-Zn-F mining area, North Eastern Nigeria. | Africa | Mining Area of Arufu, Nigeria | Water |
| An improved comprehensive model for assessing the heavy metals exposure towards waterbirds: A case report from Black-necked cranes (*Grus nigricollis*) in Caohai Wetland, China. | Asia | China | Avian Species Black-necked Cranes |
| A systematic review on metal contamination due to mining activities in the Amazon basin and associated environmental hazards. | South America | Amazon Basin, Mexico | Water and Sediments |
| Watershed-scale assessment of environmental background values of soil potential toxic elements from the Caatinga and Atlantic forest ecotone in Brazil. | South America | Caatinga and Atlantic Forest, Brazil | Soil |
| Accumulation, potential risk and source identification of toxic metal elements in soil: a case study of a coal-fired power plant in Western China. | Asia | Coal-fired Power Plant of Arid, China | Soil |
| Spatial distribution, source apportionment and potential ecological risk assessment of trace metals in surface soils in the upstream region of the Guanzhong Basin, China. | Asia | Guanzhong Basin, China | Soil |
| Perception and legacy of soil chromium and lead contamination in an operational small-scale coal mining community. | Asia | Rangpur, Bangladesh | Soil |
| Trace element contamination in soils surrounding the open-cast coal mines of eastern Raniganj basin, India. | Asia | Raniganj Basin, India | Soil |
| Spatial distribution characteristics and risk assessment of soil heavy metal pollution around typical coal gangue hill located in Fengfeng Mining area. | Asia | Fengfeng Mining Area, China | Soil |
| Pollution Characteristics, Source Identification, and Health Risk of Heavy Metals in the Soil-Vegetable System in Two Districts of Bangladesh. | Asia | Bogura and Narsingdi, Bangladesh | Soil |
| Environmental forensic approach towards unraveling contamination sources with receptor models: A case study in Nakdong River, South Korea. | Asia | Nakdong River， South Korea | Water |
| The effects of soil intake on the growth performance, rumen microbial community and tissue mineral deposition of German Mutton Merino sheep. | Asia | Heilongjiang， China | Soil and Animal(Sheep Liver and Kidney ) |
| Heavy metals distribution characteristics, source analysis, and risk evaluation of soils around mines, quarries, and other special areas in a region of northwestern Yunnan, China. | Asia | Yunnan， China | Soil |
| Assessment of Potentially Toxic Metals in Fish from Lake Manyara, Northern Tanzania. | Africa | Manyara Lake，Tanzania | Water and Fish Species |
| Bioaccessibility and children health risk assessment of soil-laden heavy metals from school playground and public parks in Accra, Ghana. | Africa | Accra， Ghana | Soil |
| Pesticide, allergen, PCB, and lead measurements in childcare centers located on tribal lands in the Pacific Northwest, United States. | North America | Portland Area Indian Country， United States | Soil and Dust |
| Impacts of a lead smelter in East Java, Indonesia: degree of contamination, spatial distribution, ecological risk, and health risk assessment of potentially toxic elements in soils. | Asia | Java， Indonesia | Soil |
| Contents and health risk assessments of selected heavy metals in vegetables produced through irrigation with effluent-impacted river. | Africa | Hirna Town of West Hararghe, Ethiopia | Water, Soil and Plant |
| Determination of heavy metals (Pb, Cr, As, Hg, and Cd) into the body organs of selected fish, water, sediment, and soil samples from Head Punjnad and Head Taunsa, Punjab, Pakistan. | Asia | Punjab, Pakistan | Water, Soil, Sediment and Fish |
| Phytoremediation strategies for heavy metal-contaminated soil by selecting native plants near mining areas in Inner Mongolia. | Asia | Inner Mongolia, China | Soil and Plant |
| Trace elements concentrations in soil contaminate corn in the vicinity of a cement-manufacturing plant: potential health implications. | Africa | Obajana, Nigeria | Soil and Plant |
| Integrated multiphase ecological risk assessment of heavy metals for migratory water birds in wetland ecosystem: A case study of Dongzhangwu Wetland, China. | Asia | Dongzhangwu Wetland, China | Avian species |
| Deterministic and Probabilistic Health Risk Assessment of Toxic Metals in the Daily Diets of Residents in Industrial Regions of Northern Ningxia, China. | Asia | Northern Ningxia, China | Water and Food |
| Assessment of roadside pollution by heavy metals: A case study from the District of Bani Kinanah, Irbid, Northern Jordan. | Asia | Irbid, Jordan | Soil |
| Mechanisms of lead uptake and accumulation in wheat grains based on atmospheric deposition-soil sources. | Asia | Zhengzhou, China | Air, Soil and Plant |
| [Translocation, Accumulation, and Comprehensive Risk Assessment of Heavy Metals in Soil-Crop Systems in an Old Industrial City, Shizuishan, Ningxia, Northwest China]. | Asia | Ningxia, China | Soil |
| Pollution Characteristics, Source Analysis, and Risk Assessment of Heavy Metals in the Surrounding Farmlands of Manganese Mining Area | Asia | Manganese Mining Area of Guizhou, China | Soil |
| Ecological risk evaluation and sensitivity analysis of heavy metals on soil organisms under human activities in the Tibet Plateau, China. | Asia | Tibet Plateau, China | Soil |
| The concentration of potentially toxic elements (PTEs) in Iranian rice: a dietary health risk assessment study. | Asia | Tehran, Iran | Food Rice |
| Artisanal gold mining in Kakamega and Vihiga counties, Kenya: potential human exposure and health risk. | Africa | Kakamega and Vihiga, Kenya | Soil |
| Concentrations and health risk appraisal of heavy metals and volatile organic compounds in soils of automobile mechanic villages in Ogun State, Nigeria. | Africa | Ogun， Nigeria | Soil |
| Geochemical composition and potential health risks of geophagic materials: an example from a rural area in the Limpopo Province of South Africa. | Africa | Fetakgomo Tubatse of Municipality Area in the Limpopo， South Africa | Soil |
| The importance of using soil series-based geochemical background values when calculating the enrichment factor in agricultural areas. | Asia | Kahramanmaras, Turkiye | Soil |
| Health risk assessment of potentially toxic elements (PTEs) concentrations in soil and fruits of selected perennial economic trees growing naturally in the vicinity of the abandoned mining ponds in Kuba, Bokkos Local Government Area (LGA) Plateau State, Nigeria. | Africa | Plateau State, Nigeria | Soil and Plant |
| Effects of atmospheric deposition on heavy metals accumulation in agricultural soils: Evidence from field monitoring and Pb isotope analysis. | Asia | China | Air and Soil |
| Elucidating Heavy Metals Concentration and Distribution in Wild Edible Morels and the Associated Soil at Different Altitudinal Zones of Pakistan: a Health Risk Implications Study. | Asia | Azad Kashmir, Murree, Swat, and Skardu of Pakistan | Soil and Plant |
| Effects of long-term zinc smelting activities on the distribution and health risk of heavy metals in agricultural soils of Guizhou province, China. | Asia | Guizhou, China | Soil |
| Oral bioaccessibility of potentially toxic elements (PTEs) and related health risk in urban playground soil from a medieval bell metal industrial town Khagra, India. | Asia | Khagra, India | Soil |
| Monitoring trace element concentrations with environmentally friendly biomonitors in Artvin, Turkiye. | Asia | Artvin, Turkiye | Soil |
| Assessing Unequal Airborne Exposure to Lead Associated With Race in the USA. | North America | United States | Air |
| Mobility, bioaccumulation in plants, and risk assessment of metals in soils. | Europ | Krakow, Poland | Soil and Plant |
| Investigating metal pollution in the food chain surrounding a lead-zinc mine (Northwestern Iran); an evaluation of health risks to humans and animals. | Asia | Arouns Mine Tailings, Iran | Soil |
| Contamination levels of and potential risks from metal(loid)s in soil-crop systems in high geological background areas. | Asia | Yunnan, China | Soil |
| Biotransfer of heavy metals along the soil-plant-edible insect-human food chain in Africa. | Africa | Kitwe, Zambia | Soil and Plant |
| Anthropogenic processes drive heterogeneous distributions of toxic elements in shallow groundwater around a smelting site. | Asia | Abandoned Smelting Site in Southern China | Water |
| Distribution, speciation, and bioaccumulation of potentially toxic elements in the grey mangroves at Indian Sundarbans, in relation to vessel movements. | Asia | Sundarbans, Indian | Soil |
| Evaluation of heavy metals contamination in cereals, vegetables and fruits with probabilistic health hazard in a highly polluted megacity. | Asia | Bangladesh | Food |
| Can mollusks or insects serve as bioindicators of the risk element polluted area? Gastropods (Gastropoda) versus leaf beetles (Coleoptera: Chrysomelidae). | Europ | Mining/Smelting Areas in Pribram of Central Bohemia, Czech | Soil and animal |
| The Concentrations, Sources, Ecological, and Human Health Risk Assessment of Heavy Metals in Roadside Soils of Six Cities in Shanxi Province, China. | Asia | Shanxi, China | Soil |
| Spatial distribution of heavy metals in soils around cement factory and health risk assessment: a case study of Canakkale-Ezine (NW Turkiye). | Asia | Canakkale-Ezine, Turkiye | Soil |
| Heavy metals in fish, rice, and human hair and health risk assessment in Wuhan city, central China. | Asia | Wuhan, China | Rice, Fish and Human Hair |
| Health risk assessment and bioaccumulation of potentially toxic metals from water, soil, and forages near coal mines of district Chakwal, Punjab, Pakistan. | Asia | Pakistan | Wastewater and Plant |
| Contamination and risk surveillance of potentially toxic elements in different land-use urban soils of Osogbo, Southwestern Nigeria. | Africa | Solid Waste Landfill of Industrial Area, Heavy Traffic Area, Residential Area with Commercial Activities of Nigeria | Soil |
| Human health risk associated with metal exposure at Agbogbloshie e-waste site and the surrounding neighbourhood in Accra, Ghana. | Africa | Accra Burning and Dismantling Sites of Ghana | Soil |
| Environmental risk associated with accumulation of toxic metalloids in soils of the Odra River floodplain-case study of the assessment based on total concentrations, fractionation and geochemical indices. | Europ | Odra River, Poland | Soil |
| Assessment of potentially toxic metal(loid)s contamination in soil near the industrial landfill and impact on human health: an evaluation of risk. | Asia | Gujrat, Pakistan | Soil |
| Spatial distribution, sources and health risk assessment of heavy metals in topsoil around oil and natural gas drilling sites, Andhra Pradesh, India. | Asia | Andhra Pradesh, India | Soil |
| Evaluation of heavy metals in ground and surface water in Ranipet, India utilizing HPI model. | Asia | Ranipet, India | Water |
| Distribution of Minor and Major Metallic Elements in Residential Indoor Dust: A Case Study in Latvia. | Europ | Riga, Latvia | Dust |
| Pollution and risk assessment of potentially toxic elements in soils from industrial and mining sites across China. | Asia | Industrial and Mining Sites of China | Soil |
| Pollution Characteristics and Risk Assessment of Heavy Metals in Surface Dusts and Surrounding Green Land Soils from Yellow River Custom Tourist Line in Lanzhou | Asia | Yellow River Custom Tourist Line in Lanzhou, China | Soil and Dust |
| Seasonal variation and risks of potentially toxic elements in agricultural lowlands of central Cameroon. | Africa | Cameroon | Soil |
| Environmental impacts of an unlined municipal solid waste landfill on groundwater and surface water quality in Ibadan, Nigeria. | Africa | Ibadan, Nigeria | Water |
| Understanding heavy metal distribution in timberline vegetations: A case from the Gongga Mountain, eastern Tibetan Plateau. | Asia | Tibetan Plateau, China | Plant (Branch and Bark) |
| Long-Term Changes in the Pollution of Warta River Bottom Sediments with Heavy Metals, Poland-Case Study. | Europ | Warta River, Poland | Deposit Sediment |
| Pollution evaluation and source identification of heavy metals in soil around steel factories located in Lanshan District, Rizhao City, eastern China. | Asia | Rizhao in LanshanDistrict, China | Soil |
| Spatial distribution and pollution evaluation in dry riverbeds affected by mine tailings. | Europ | Mar Menor, Spain | Deposit Sediment |
| Effect of past century mining activities on sediment properties and toxicity to freshwater organisms in northern Sweden. | Europ | Sweden | Deposit Sediment |
| Active moss biomonitoring of airborne potentially toxic elements in recreational areas of Moscow. | Europ | Moscow, Russia | Moss |
| Phytoaccumulation of trace elements (As, Cd, Co, Cu, Pb, Zn) by *Nicotiana glauca* and *Euphorbia segetalis* growing in a Technosol developed on legacy mine wastes (Domingo Rubio wetland, SW Spain). | Europ | Domingo Rubio Wetland, Spain | Soil and Plant |
| Temporal and spatial trends in lead levels in the blood and down of Black Stork nestlings in central Europe. | Europ | Poland | Avian Species Black Stork Nestlings |
| 12 Years of honey surveys in northern Italy: How anthropic activities can influence honey quality. | Europ | Lombardy, Italy | Honey |
| Long-term monitoring of exposure to toxic and essential metals and metalloids in the tawny owl (*Strix aluco*): Temporal trends and influence of spatial patterns. | Europ | France | Avian species Tawny Owl |

## **Table S2.** Refined Pb metal. producing countries with an output above 100,000 tonnes in 2022.

| **Counties and Regions** | **Refined Pb Metal Producing Amount (kT)** |
| --- | --- |
| China | 5226 |
| India | 966 |
| United States | 953 |
| South Korea | 773 |
| Mexico | 437 |
| United Kingdom | 307 |
| Japan | 295 |
| Brazil | 278 |
| Germany | 251 |
| Canada | 194 |
| Spain | 192 |
| Russian Federation | 180 |
| Australia | 164 |
| Poland | 153 |
| Italy | 141 |
| Iran | 125 |
| Kazakhstan | 121 |
| Belgium | 110 |
| Bulgaria | 105 |

## **Table S3.** The primer sequences synthesis for mtDNA analysis.

| **Gene** | **ID** | **Forward** | **Reverse** |
| --- | --- | --- | --- |
| ND2 | NC_001606.1 | AGGCGCCACAGTAATCACAA | TTGCGGGGGTGAAGTTTTCT |
| COX4I1 | LOC109049772 | GCTGTCGTACGCAGAGATGA | AATCCATGGACGGGGTTCAC |
| CYTB | NC_018035.1 | AGGCGCCACAGTAATCACAA | TTGCGGGGGTGAAGTTTTCT |
| TERT | XM_042745372.1 | TGCTGAAGGGCATCGCTAAA | GCAGCTTCTTTATGACCGCG |

## **Table S4.** Statistical table of sequencing data.

| Sample | Raw reads | Clean reads | Non-unique | Unique | Mapped rate | Concordant pair alignment rate |
| --- | --- | --- | --- | --- | --- | --- |
| Control.1 | 53898548 | 45003060 | 10291763 | 34711297 | 83.50% | 80.47% |
| Control.2 | 48425690 | 39942387 | 7894233 | 32048154 | 82.48% | 78.29% |
| Control.3 | 45551844 | 37766162 | 7821826 | 29944336 | 82.91% | 78.47% |
| Mel.1 | 49971962 | 39909555 | 5921708 | 33987847 | 79.86% | 75.37% |
| Mel.2 | 45722500 | 36599938 | 5424193 | 31175745 | 80.05% | 75.44% |
| Mel.3 | 35372694 | 28073843 | 4200064 | 23873779 | 79.37% | 74.73% |
| Pb.1 | 43995736 | 36195046 | 6758463 | 29436583 | 82.27% | 78.35% |
| Pb.2 | 46507742 | 38399130 | 7593391 | 30805739 | 82.57% | 79.00% |
| Pb.3 | 44897958 | 37223221 | 7209432 | 30013789 | 82.91% | 79.44% |
| Pb.Mel.1 | 56392406 | 45046584 | 6461742 | 38584842 | 79.88% | 75.24% |
| Pb.Mel.2 | 40933972 | 32916402 | 4526990 | 28389412 | 80.41% | 75.86% |
| Pb.Mel.2 | 40933972 | 32916402 | 4526990 | 29200540 | 80.95% | 76.48% |

## **Table S5.** The inventory of software used in transcriptomics and targeted metabolomics analysis.

| **Analysis** | **Tool** | **Version** |
| --- | --- | --- |
| RNA-seq | HISAT2 | 2.2.1 |
| RNA-seq | DESeq2 | 1.34.0 |
| Metabolomics | Skyline | 21.1 |
| Pathway | clusterProfiler | 4.2.2 |

## **Table S6.** The subset of DEGs between the Pb and Control groups using thresholds of FDR < 0.05.

| **Gene ID** | **Log_2_\|Fold change\| (Pb vs. Control)** | ***P* Value** | **FDR (padj)** |
| --- | --- | --- | --- |
| ENSCCRG00000027595 | 4.686798416 | 8.80552E-14 | 1.42368E-09 |
| ENSCCRG00000029429 | -2.554102419 | 5.73895E-13 | 6.18583E-09 |
| ENSCCRG00000016592 | 3.959209211 | 3.71354E-11 | 3.00202E-07 |
| ENSCCRG00000057815 | 6.449603607 | 3.66497E-10 | 2.00232E-06 |
| ENSCCRG00000011235 | -2.222641037 | 3.71534E-10 | 2.00232E-06 |
| ENSCCRG00000040063 | 3.711186411 | 6.26057E-10 | 2.89203E-06 |
| ENSCCRG00000046355 | -2.921816716 | 1.80788E-09 | 6.74031E-06 |
| ENSCCRG00000038928 | 4.127387991 | 1.87601E-09 | 6.74031E-06 |
| ENSCCRG00000026810 | 3.883043638 | 2.12098E-09 | 6.8584E-06 |
| ENSCCRG00000036877 | 5.994983046 | 9.62108E-09 | 2.82825E-05 |
| ENSCCRG00000028197 | 1.670196683 | 2.10341E-08 | 5.66798E-05 |
| ENSCCRG00000006614 | 8.007120627 | 2.61355E-08 | 6.50092E-05 |
| ENSCCRG00000016320 | 4.542180482 | 2.94285E-08 | 6.79715E-05 |
| ENSCCRG00000007374 | 7.127559122 | 3.38415E-08 | 7.29532E-05 |
| ENSCCRG00000020927 | -2.083204293 | 4.13849E-08 | 8.36388E-05 |
| ENSCCRG00000075606 | 3.937239207 | 4.69425E-08 | 8.92901E-05 |
| ENSCCRG00000016788 | 3.643248912 | 7.25788E-08 | 0.000126002 |
| ENSCCRG00000035121 | 5.258430632 | 7.40364E-08 | 0.000126002 |
| ENSCCRG00000002396 | 3.140694477 | 8.21819E-08 | 0.000132872 |
| ENSCCRG00000049931 | 2.733104918 | 1.20892E-07 | 0.000186151 |
| ENSCCRG00000025239 | 6.582005725 | 1.50221E-07 | 0.000220104 |
| ENSCCRG00000046168 | -5.348166974 | 1.56556E-07 | 0.000220104 |
| ENSCCRG00000022043 | -2.033049694 | 1.71567E-07 | 0.000228884 |
| ENSCCRG00000048651 | -3.366238192 | 1.79939E-07 | 0.000228884 |
| ENSCCRG00000029096 | 5.85711602 | 1.84036E-07 | 0.000228884 |
| ENSCCRG00000051086 | -3.702231319 | 1.93891E-07 | 0.000232209 |
| ENSCCRG00000026157 | 3.799158026 | 2.3715E-07 | 0.00027255 |
| ENSCCRG00000018675 | -1.729911909 | 2.44432E-07 | 0.00027255 |
| ENSCCRG00000022899 | 4.110347277 | 2.54581E-07 | 0.000274404 |
| ENSCCRG00000043255 | 4.172725823 | 3.40068E-07 | 0.000354723 |
| ENSCCRG00000047473 | 4.450561741 | 4.2779E-07 | 0.000421061 |
| ENSCCRG00020039544 | -8.349743692 | 4.40192E-07 | 0.000421061 |
| ENSCCRG00000017298 | -1.325323363 | 4.42728E-07 | 0.000421061 |
| ENSCCRG00000014564 | 1.820869082 | 4.7389E-07 | 0.00043782 |
| ENSCCRG00000043820 | 3.780119165 | 5.48449E-07 | 0.00048879 |
| ENSCCRG00000012370 | 8.100443102 | 5.59291E-07 | 0.00048879 |
| ENSCCRG00000002176 | 6.16217513 | 6.03143E-07 | 0.000511866 |
| ENSCCRG00000027023 | 2.435650647 | 6.20867E-07 | 0.000511866 |
| ENSCCRG00000005495 | 4.597735298 | 6.33184E-07 | 0.000511866 |
| ENSCCRG00000036257 | -1.3910564 | 6.55376E-07 | 0.000516884 |
| ENSCCRG00000024277 | -1.826794372 | 7.36685E-07 | 0.000567177 |
| ENSCCRG00000050792 | 3.741364748 | 7.66532E-07 | 0.000576432 |
| ENSCCRG00000013831 | -1.356767405 | 7.86829E-07 | 0.000578248 |
| ENSCCRG00000039217 | -1.504762834 | 9.81195E-07 | 0.000705065 |
| ENSCCRG00000037860 | -2.364161974 | 1.32633E-06 | 0.000932349 |
| ENSCCRG00000036364 | -1.136623036 | 1.39223E-06 | 0.000946637 |
| ENSCCRG00000009596 | 4.16333986 | 1.42589E-06 | 0.000946637 |
| ENSCCRG00000042642 | 5.556463782 | 1.43448E-06 | 0.000946637 |
| ENSCCRG00000051148 | 7.113846824 | 1.5076E-06 | 0.000974994 |
| ENSCCRG00000027777 | 3.510014757 | 1.6615E-06 | 0.00103066 |
| ENSCCRG00000007011 | 4.138486147 | 1.68016E-06 | 0.00103066 |
| ENSCCRG00000070078 | 4.061812398 | 1.68929E-06 | 0.00103066 |
| ENSCCRG00000006973 | 2.063125922 | 2.55063E-06 | 0.001527355 |
| ENSCCRG00000047902 | -2.02822967 | 2.70279E-06 | 0.001578428 |
| ENSCCRG00000010087 | 3.504181646 | 2.73355E-06 | 0.001578428 |
| ENSCCRG00000012855 | 6.56988585 | 2.78552E-06 | 0.001580223 |
| ENSCCRG00000046505 | 1.347050198 | 2.93313E-06 | 0.001635269 |
| ENSCCRG00000007581 | -1.223935912 | 3.46991E-06 | 0.001901745 |
| ENSCCRG00000060419 | 2.747433469 | 4.26978E-06 | 0.002301128 |
| ENSCCRG00000008398 | -2.074663859 | 4.45387E-06 | 0.00235757 |
| ENSCCRG00000048424 | 4.923330038 | 4.52033E-06 | 0.00235757 |
| ENSCCRG00000026080 | 2.137615085 | 4.65853E-06 | 0.002391081 |
| ENSCCRG00000024700 | -1.760186043 | 4.77608E-06 | 0.002413116 |
| ENSCCRG00000032574 | 4.54281162 | 5.05795E-06 | 0.002516216 |
| ENSCCRG00000050855 | 3.355058774 | 6.71752E-06 | 0.003266775 |
| ENSCCRG00000049449 | 2.331369501 | 6.76874E-06 | 0.003266775 |
| ENSCCRG00000027031 | -4.141902538 | 6.92503E-06 | 0.003293058 |
| ENSCCRG00000022066 | 7.737381409 | 7.25793E-06 | 0.003401338 |
| ENSCCRG00000022373 | -1.393595493 | 7.47565E-06 | 0.003446785 |
| [ENSCCRG00000082607](https://www.ensembl.org/id/ENSCCRG00000082607.1) | 6.288441869 | 8.22546E-06 | 0.003694147 |
| ENSCCRG00000041669 | 4.061234745 | 9.01171E-06 | 0.003991816 |
| ENSCCRG00000014479 | -2.503580442 | 1.0624E-05 | 0.004642395 |
| ENSCCRG00000021725 | -1.185542615 | 1.15284E-05 | 0.004970415 |
| ENSCCRG00000030941 | 3.702223744 | 1.1794E-05 | 0.00501802 |
| ENSCCRG00000043588 | -2.010948336 | 1.35777E-05 | 0.005701947 |
| ENSCCRG00000051895 | -1.951675207 | 1.48495E-05 | 0.006090642 |
| ENSCCRG00000009930 | 3.616246748 | 1.488E-05 | 0.006090642 |
| ENSCCRG00000017093 | -2.679968305 | 1.62244E-05 | 0.006557903 |
| ENSCCRG00000040493 | -1.399436285 | 1.80326E-05 | 0.0071988 |
| ENSCCRG00000004923 | 1.42704362 | 2.25943E-05 | 0.008883378 |
| ENSCCRG00000011326 | -1.725392248 | 2.28018E-05 | 0.008883378 |
| ENSCCRG00000041530 | 1.887069234 | 2.50312E-05 | 0.009580831 |
| ENSCCRG00000044325 | -2.498384512 | 2.51846E-05 | 0.009580831 |
| ENSCCRG00000033333 | 1.655246272 | 2.55929E-05 | 0.009622943 |
| ENSCCRG00000019153 | -1.693727241 | 2.64462E-05 | 0.009829463 |
| ENSCCRG00000024283 | 2.840623808 | 2.82934E-05 | 0.010396549 |
| ENSCCRG00000023887 | 2.593069298 | 2.89345E-05 | 0.010512648 |
| ENSCCRG00000031943 | -1.102839681 | 2.93999E-05 | 0.01056306 |
| ENSCCRG00000031146 | 2.900640178 | 3.03119E-05 | 0.010771055 |
| ENSCCRG00000002395 | 2.681310051 | 3.17417E-05 | 0.011110927 |
| ENSCCRG00000026762 | 4.225173992 | 3.19556E-05 | 0.011110927 |
| ENSCCRG00000032333 | 5.78925673 | 3.45415E-05 | 0.011827974 |
| ENSCCRG00000028150 | 1.94157032 | 3.51079E-05 | 0.011827974 |
| ENSCCRG00000000691 | 1.663862714 | 3.51152E-05 | 0.011827974 |
| ENSCCRG00000014657 | -1.481538229 | 3.75118E-05 | 0.012504966 |
| ENSCCRG00000034932 | 6.340270685 | 3.93139E-05 | 0.01297197 |
| ENSCCRG00000019217 | 2.645047824 | 4.07103E-05 | 0.013297038 |
| ENSCCRG00000042463 | -1.62827438 | 4.15416E-05 | 0.013355906 |
| ENSCCRG00000042398 | 4.432694643 | 4.17166E-05 | 0.013355906 |
| ENSCCRG00000023681 | 4.944188596 | 4.25752E-05 | 0.013373202 |
| ENSCCRG00000036591 | 4.531394065 | 4.28752E-05 | 0.013373202 |
| ENSCCRG00000010204 | 5.596881588 | 4.30113E-05 | 0.013373202 |
| ENSCCRG00000002244 | 2.718425005 | 4.45851E-05 | 0.013730525 |
| ENSCCRG00000013067 | -2.612128834 | 4.526E-05 | 0.013806867 |
| ENSCCRG00000003151 | 1.315808327 | 4.59535E-05 | 0.013887418 |
| ENSCCRG00000005132 | 1.484707819 | 4.81676E-05 | 0.014330468 |
| ENSCCRG00000041719 | 4.191070598 | 4.83059E-05 | 0.014330468 |
| ENSCCRG00000042808 | -1.475139277 | 5.16365E-05 | 0.015179247 |
| ENSCCRG00000001171 | 1.336952932 | 5.30253E-05 | 0.015447074 |
| ENSCCRG00000042573 | -1.607025125 | 5.42552E-05 | 0.015664253 |
| ENSCCRG00000001135 | 1.976949035 | 5.66113E-05 | 0.01619984 |
| ENSCCRG00000011376 | -1.22897054 | 5.71471E-05 | 0.016209712 |
| ENSCCRG00000009428 | 4.366521836 | 6.01899E-05 | 0.016924352 |
| ENSCCRG00000008685 | 3.982891417 | 6.15126E-05 | 0.017147172 |
| ENSCCRG00000029734 | 2.478687649 | 6.44599E-05 | 0.017707409 |
| ENSCCRG00000021591 | -2.123769942 | 6.46176E-05 | 0.017707409 |
| ENSCCRG00000026159 | -7.351382755 | 6.5719E-05 | 0.017857891 |
| ENSCCRG00000002432 | -1.330578906 | 7.49095E-05 | 0.019965514 |
| ENSCCRG00000003468 | 3.575346164 | 7.51671E-05 | 0.019965514 |
| ENSCCRG00000010468 | -3.675048166 | 7.53276E-05 | 0.019965514 |
| ENSCCRG00000013724 | 3.347952221 | 7.6262E-05 | 0.020048833 |
| ENSCCRG00000049072 | 3.234263269 | 7.81756E-05 | 0.02014985 |
| ENSCCRG00000024177 | 3.669252796 | 7.83744E-05 | 0.02014985 |
| ENSCCRG00000044634 | 4.495304539 | 7.88211E-05 | 0.02014985 |
| ENSCCRG00000041660 | 3.34765893 | 7.9353E-05 | 0.02014985 |
| ENSCCRG00000012803 | 5.069273919 | 7.97619E-05 | 0.02014985 |
| ENSCCRG00000022321 | 3.404958894 | 8.32338E-05 | 0.020863946 |
| ENSCCRG00000011768 | 5.557573511 | 8.5987E-05 | 0.021388265 |
| ENSCCRG00000031432 | 5.904447148 | 8.87404E-05 | 0.021904642 |
| ENSCCRG00000015789 | -1.06398623 | 9.38291E-05 | 0.022985283 |
| ENSCCRG00000022417 | 1.696818995 | 9.84054E-05 | 0.023877296 |
| ENSCCRG00000030012 | 2.640399573 | 9.89472E-05 | 0.023877296 |
| ENSCCRG00000048278 | 1.491518895 | 0.000101871 | 0.024400659 |
| ENSCCRG00000001555 | -1.053811202 | 0.000104123 | 0.02475685 |
| ENSCCRG00000033055 | -3.46306649 | 0.000105229 | 0.024837111 |
| ENSCCRG00000026232 | 2.044047031 | 0.000107213 | 0.025122077 |
| ENSCCRG00000003745 | -2.263777146 | 0.000108703 | 0.025287849 |
| ENSCCRG00000017214 | -2.442238331 | 0.000110389 | 0.025319272 |
| ENSCCRG00000002023 | 1.106044788 | 0.000110404 | 0.025319272 |
| ENSCCRG00000046934 | 1.234245714 | 0.00011985 | 0.027292037 |
| ENSCCRG00000002111 | 1.469281201 | 0.000135032 | 0.030534317 |
| ENSCCRG00000011041 | 3.462304349 | 0.000139381 | 0.031298741 |
| ENSCCRG00000026869 | 5.068476012 | 0.000145553 | 0.032459248 |
| ENSCCRG00000048281 | 4.093844758 | 0.000154417 | 0.034200238 |
| ENSCCRG00000028745 | 4.100136672 | 0.000157378 | 0.034618889 |
| ENSCCRG00000036917 | -1.496976811 | 0.000160637 | 0.03501061 |
| ENSCCRG00000034567 | 5.133016212 | 0.000161324 | 0.03501061 |
| ENSCCRG00000016854 | -1.654266082 | 0.000166561 | 0.035906075 |
| ENSCCRG00000022178 | -2.851598312 | 0.000167925 | 0.035960429 |
| ENSCCRG00000015822 | 1.462254351 | 0.000173254 | 0.036857607 |
| ENSCCRG00000016984 | 2.871364689 | 0.00017446 | 0.036871468 |
| ENSCCRG00000005928 | -2.885345341 | 0.000181605 | 0.038132378 |
| ENSCCRG00000017251 | 3.547519445 | 0.000188413 | 0.039116529 |
| ENSCCRG00000037968 | 1.261821851 | 0.000189221 | 0.039116529 |
| ENSCCRG00000001230 | -3.460005254 | 0.000190335 | 0.039116529 |
| ENSCCRG00000026886 | 3.128065463 | 0.000192007 | 0.039116529 |
| ENSCCRG00000019976 | -1.048523476 | 0.000192341 | 0.039116529 |
| ENSCCRG00000018302 | 1.881869491 | 0.000199036 | 0.040225165 |
| ENSCCRG00000007862 | 3.611757985 | 0.000201109 | 0.040391665 |
| ENSCCRG00000047831 | 2.120616619 | 0.000204597 | 0.040838517 |
| ENSCCRG00000047664 | 1.381691639 | 0.000208312 | 0.041324945 |
| ENSCCRG00000003066 | -1.524566185 | 0.0002122 | 0.041586114 |
| ENSCCRG00000007334 | -1.553993156 | 0.000219231 | 0.042705224 |
| ENSCCRG00000001386 | -1.4230497 | 0.000223721 | 0.043318841 |
| ENSCCRG00000007788 | -1.033144553 | 0.000227327 | 0.043754947 |
| ENSCCRG00000050289 | 6.031250438 | 0.000229927 | 0.043993624 |
| ENSCCRG00000043676 | -6.025776474 | 0.000235583 | 0.044715662 |
| ENSCCRG00000012692 | 3.41534922 | 0.000236466 | 0.044715662 |
| ENSCCRG00000015485 | 1.778471594 | 0.000249417 | 0.04689045 |
| ENSCCRG00000002342 | 2.71303899 | 0.000251695 | 0.04704515 |
| ENSCCRG00000036544 | 3.048036807 | 0.000253367 | 0.047085538 |
| ENSCCRG00000007916 | 3.600483715 | 0.000258492 | 0.047763419 |
| ENSCCRG00000026579 | 2.123811231 | 0.000264027 | 0.048040556 |
| ENSCCRG00000021699 | -1.344812307 | 0.000264192 | 0.048040556 |
| ENSCCRG00000025167 | 1.767531403 | 0.000264449 | 0.048040556 |
| ENSCCRG00000018917 | -1.391742412 | 0.00027645 | 0.049786275 |
| ENSCCRG00000029767 | 4.451153591 | 0.000277138 | 0.049786275 |

## **Table S7.** The subset of DEGs between the Pb.Mel and Pb groups using thresholds of FDR < 0.05.

| **Gene ID** | **Log_2_\|Fold change\| (Pb.Mel vs. Pb)** | ***P* Value** | **FDR (padj)** |
| --- | --- | --- | --- |
| ENSCCRG00000038928 | -3.524210761 | 4.2096E-17 | 6.57623E-13 |
| ENSCCRG00000075606 | -3.088996449 | 7.47747E-14 | 7.78754E-10 |
| ENSCCRG00000045884 | 3.098345762 | 1.55877E-16 | 4.87021E-10 |
| ENSCCRG00000028815 | -8.361788164 | 1.28468E-13 | 1.00347E-09 |
| ENSCCRG00000002344 | 2.73231278 | 4.0629E-13 | 2.53883E-09 |
| ENSCCRG00000024277 | 2.598553687 | 1.30817E-12 | 6.8121E-09 |
| ENSCCRG00000060419 | 2.978644242 | 2.63946E-12 | 1.1781E-08 |
| ENSCCRG00000021077 | -2.95086886 | 4.54868E-12 | 1.77649E-08 |
| ENSCCRG00000047473 | -6.067914124 | 1.39586E-11 | 4.37572E-08 |
| ENSCCRG00000011326 | 2.541926578 | 1.4005E-11 | 4.37572E-08 |
| ENSCCRG00000018675 | 8.315773207 | 4.53696E-11 | 1.28866E-07 |
| ENSCCRG00000014479 | 3.526010013 | 1.9495E-10 | 5.07584E-07 |
| /ENSCCRG00000012575 | 3.935717637 | 2.43821E-10 | 5.85995E-07 |
| ENSCCRG00000017093 | 3.713209159 | 2.94654E-10 | 6.37188E-07 |
| ENSCCRG00000040063 | -2.621534289 | 3.05909E-10 | 6.37188E-07 |
| ENSCCRG00000048651 | 3.878722508 | 6.63737E-10 | 1.29611E-06 |
| ENSCCRG00000070078 | -5.504998541 | 2.33966E-09 | 4.30002E-06 |
| ENSCCRG00000006110 | 2.800680876 | 5.40748E-09 | 9.38618E-06 |
| ENSCCRG00000032884 | -8.730660892 | 7.39388E-09 | 1.21587E-05 |
| ENSCCRG00000010521 | 3.201967108 | 1.07024E-08 | 1.67192E-05 |
| ENSCCRG00000036583 | -2.76582604 | 1.27271E-08 | 1.8334E-05 |
| ENSCCRG00000000020 | -6.294416827 | 1.29096E-08 | 1.8334E-05 |
| ENSCCRG00000025287 | -3.714733068 | 1.39636E-08 | 1.89687E-05 |
| ENSCCRG00000045975 | 3.777268058 | 1.77138E-08 | 2.30604E-05 |
| ENSCCRG00000033829 | -3.349802835 | 5.00193E-08 | 6.25121E-05 |
| ENSCCRG00000005495 | -4.323347886 | 2.27008E-07 | 0.000272794 |
| ENSCCRG00000025366 | 3.02148253 | 2.82032E-07 | 0.000326363 |
| ENSCCRG00000017226 | -4.892345763 | 3.48466E-07 | 0.000388838 |
| ENSCCRG00000045610 | -2.095830821 | 3.78917E-07 | 0.000408237 |
| ENSCCRG00000001566 | -8.622312709 | 4.28429E-07 | 0.000446194 |
| ENSCCRG00000044389 | -5.453317922 | 5.20023E-07 | 0.000524116 |
| ENSCCRG00000047588 | -5.934090491 | 5.82888E-07 | 0.000569117 |
| ENSCCRG00000036257 | 1.35921012 | 6.75997E-07 | 0.000640026 |
| ENSCCRG00015004052 | -5.1461204 | 7.25574E-07 | 0.00066676 |
| ENSCCRG00000034862 | -2.934606366 | 1.02059E-06 | 0.000911069 |
| ENSCCRG00000026886 | -2.872968019 | 1.24958E-06 | 0.001066959 |
| ENSCCRG00000044568 | -2.417530198 | 1.26352E-06 | 0.001066959 |
| ENSCCRG00000041844 | -2.68850682 | 1.77264E-06 | 0.001457483 |
| ENSCCRG00000002048 | -4.012322574 | 1.9376E-06 | 0.00155227 |
| ENSCCRG00000023664 | -4.70136651 | 1.98851E-06 | 0.001553226 |
| ENSCCRG00000018841 | -7.138047954 | 2.10307E-06 | 0.001602641 |
| ENSCCRG00000023670 | -7.938300584 | 2.37408E-06 | 0.001766092 |
| ENSCCRG00000002508 | -5.058155839 | 2.4708E-06 | 0.001795294 |
| ENSCCRG00000050533 | -4.857727837 | 3.06352E-06 | 0.002175378 |
| ENSCCRG00000028699 | -1.886929551 | 3.71319E-06 | 0.002578112 |
| ENSCCRG00000047609 | -2.332267151 | 4.65802E-06 | 0.00316381 |
| ENSCCRG00000019664 | -3.869073089 | 5.81286E-06 | 0.003864193 |
| ENSCCRG00000009197 | -6.339518248 | 6.49716E-06 | 0.004227408 |
| ENSCCRG00000047061 | -2.099730325 | 6.62985E-06 | 0.004227408 |
| ENSCCRG00000009428 | -4.991099586 | 8.55211E-06 | 0.005344043 |
| ENSCCRG00000018705 | -1.867415296 | 9.37317E-06 | 0.005742263 |
| ENSCCRG00000044020 | -5.364493662 | 9.96244E-06 | 0.005985897 |
| ENSCCRG00000006965 | -2.526131067 | 1.03075E-05 | 0.006076363 |
| ENSCCRG00000012900 | -3.62370188 | 1.05143E-05 | 0.006083522 |
| ENSCCRG00000041846 | -3.301477068 | 1.13932E-05 | 0.006472174 |
| ENSCCRG00000007083 | -1.913223924 | 1.35155E-05 | 0.0075407 |
| ENSCCRG00000008398 | 2.534076751 | 1.39524E-05 | 0.007647865 |
| ENSCCRG00000049931 | -1.145887373 | 1.52903E-05 | 0.008194825 |
| ENSCCRG00000034933 | -2.004250309 | 1.54748E-05 | 0.008194825 |
| ENSCCRG00000049041 | 2.3484806 | 1.59533E-05 | 0.008307438 |
| ENSCCRG00000022921 | 1.574937657 | 1.64144E-05 | 0.008329581 |
| ENSCCRG00000006582 | -2.840056444 | 1.65291E-05 | 0.008329581 |
| ENSCCRG00000004766 | -1.671325977 | 1.81776E-05 | 0.00901495 |
| ENSCCRG00000052269 | 2.610960365 | 2.25701E-05 | 0.011018457 |
| ENSCCRG00000006314 | -2.784192779 | 2.32949E-05 | 0.011197328 |
| ENSCCRG00000039203 | 2.602528693 | 2.80533E-05 | 0.013280275 |
| ENSCCRG00000034556 | -2.238522263 | 2.86522E-05 | 0.013361337 |
| ENSCCRG00000019684 | -4.060035807 | 3.07948E-05 | 0.013981093 |
| ENSCCRG00000039781 | 1.943871026 | 3.08762E-05 | 0.013981093 |
| ENSCCRG00000018267 | -3.569326964 | 3.41118E-05 | 0.0149679 |
| ENSCCRG00000006969 | -1.733462983 | 3.41952E-05 | 0.0149679 |
| ENSCCRG00000022066 | -5.248305495 | 3.44927E-05 | 0.0149679 |
| ENSCCRG00000024706 | 1.712159188 | 3.70485E-05 | 0.015856778 |
| ENSCCRG00000013589 | 1.519646396 | 3.82872E-05 | 0.016165479 |
| ENSCCRG00000046820 | 1.271806012 | 3.98696E-05 | 0.016609128 |
| ENSCCRG00000029920 | -6.599708461 | 4.18528E-05 | 0.01720591 |
| ENSCCRG00000012996 | -3.389478128 | 4.3786E-05 | 0.017766888 |
| ENSCCRG00000026359 | 4.007853827 | 4.75412E-05 | 0.018847023 |
| ENSCCRG00000042524 | 7.619051423 | 4.76544E-05 | 0.018847023 |
| ENSCCRG00000020778 | 3.480080448 | 4.97464E-05 | 0.019007518 |
| ENSCCRG00000000546 | 1.320681674 | 5.01524E-05 | 0.019007518 |
| ENSCCRG00000015408 | -4.253330281 | 5.01885E-05 | 0.019007518 |
| ENSCCRG00000025974 | -3.657400412 | 5.04937E-05 | 0.019007518 |
| ENSCCRG00000000740 | -10.09387124 | 5.27136E-05 | 0.019606941 |
| ENSCCRG00000001386 | 1.860245078 | 5.38616E-05 | 0.019798251 |
| ENSCCRG00000043172 | -4.742996867 | 5.58418E-05 | 0.020287446 |
| ENSCCRG00000009741 | 7.9078908 | 5.76208E-05 | 0.020693139 |
| ENSCCRG00000019153 | 1.534413278 | 6.7644E-05 | 0.02401671 |
| ENSCCRG00000013356 | 2.840200048 | 6.98069E-05 | 0.024076638 |
| ENSCCRG00000024877 | -2.228430074 | 6.99055E-05 | 0.024076638 |
| ENSCCRG00000012278 | -6.899847857 | 7.06886E-05 | 0.024076638 |
| ENSCCRG00000048280 | 2.972029917 | 7.08952E-05 | 0.024076638 |
| ENSCCRG00000041782 | 1.689284756 | 7.2172E-05 | 0.024246673 |
| ENSCCRG00000050062 | -2.258043395 | 7.46513E-05 | 0.024812811 |
| ENSCCRG00000024326 | -2.434681293 | 8.30997E-05 | 0.027330183 |
| ENSCCRG00000006022 | -7.083046423 | 9.25209E-05 | 0.030111712 |
| ENSCCRG00000007316 | 2.236389162 | 9.77311E-05 | 0.031435915 |
| ENSCCRG00000009864 | 1.777318399 | 9.91101E-05 | 0.031435915 |
| ENSCCRG00000029767 | -4.128736375 | 9.96081E-05 | 0.031435915 |
| ENSCCRG00000002672 | -3.085743085 | 0.00010233 | 0.031828235 |
| ENSCCRG00000032845 | -1.402936105 | 0.000102889 | 0.031828235 |
| ENSCCRG00000023035 | 2.045475691 | 0.000113793 | 0.034856229 |
| ENSCCRG00000039096 | 1.521771086 | 0.000116103 | 0.035218733 |
| ENSCCRG00000030104 | -2.625440354 | 0.000118586 | 0.03562591 |
| ENSCCRG00000000096 | -3.081664934 | 0.000125065 | 0.037214541 |
| ENSCCRG00000018782 | -3.536983979 | 0.000128403 | 0.037847521 |
| ENSCCRG00000036252 | -1.444894333 | 0.000133507 | 0.038984026 |
| ENSCCRG00000052633 | -3.224854121 | 0.000135037 | 0.03906568 |
| ENSCCRG00000043929 | -3.110597029 | 0.000142153 | 0.040272481 |
| ENSCCRG00000047083 | 4.875256699 | 0.000142471 | 0.040272481 |
| ENSCCRG00000036293 | -2.967464893 | 0.000145278 | 0.040272481 |
| ENSCCRG00000017562 | -2.274574136 | 0.000146578 | 0.040272481 |
| ENSCCRG00000021122 | -2.426722911 | 0.000148269 | 0.040272481 |
| ENSCCRG00000014681 | 1.796819692 | 0.000149049 | 0.040272481 |
| ENSCCRG00000035797 | 6.788643628 | 0.000150876 | 0.040272481 |
| ENSCCRG00000007127 | -1.678080617 | 0.000151674 | 0.040272481 |
| ENSCCRG00000006583 | -2.802960049 | 0.000151679 | 0.040272481 |
| ENSCCRG00000023570 | -3.153148066 | 0.000152098 | 0.040272481 |
| ENSCCRG00000040609 | 1.806653791 | 0.000157103 | 0.041109503 |
| ENSCCRG00000048855 | -2.156702499 | 0.000157891 | 0.041109503 |
| ENSCCRG00000057815 | -4.721411773 | 0.000164473 | 0.041914237 |
| ENSCCRG00000027188 | -2.588166088 | 0.000164695 | 0.041914237 |
| ENSCCRG00000003960 | 3.710887346 | 0.000165006 | 0.041914237 |
| ENSCCRG00000004875 | -1.89572902 | 0.000168707 | 0.042508834 |
| ENSCCRG00000046660 | -2.184977643 | 0.000173345 | 0.043327949 |
| ENSCCRG00000031129 | -1.567001273 | 0.000179402 | 0.044227797 |
| ENSCCRG00000046182 | 4.688241822 | 0.000180974 | 0.044227797 |
| ENSCCRG00000048251 | -5.550429995 | 0.000181192 | 0.044227797 |
| ENSCCRG00000011099 | -3.439321676 | 0.00019012 | 0.046047379 |
| ENSCCRG00000043483 | 4.322161516 | 0.000191888 | 0.046117961 |
| ENSCCRG00000006387 | 3.382856228 | 0.000197116 | 0.046486266 |
| ENSCCRG00000003468 | -3.735254464 | 0.000197143 | 0.046486266 |
| ENSCCRG00000034782 | 1.68984487 | 0.000197966 | 0.046486266 |
| ENSCCRG00000046355 | 2.671535161 | 0.000199371 | 0.046486266 |
| ENSCCRG00000033784 | 2.088680013 | 0.000201796 | 0.046622117 |
| ENSCCRG00000012072 | -1.650463386 | 0.000202938 | 0.046622117 |
| ENSCCRG00000035009 | -6.349814362 | 0.000214733 | 0.048971711 |
| ENSCCRG00000005868 | -1.011335055 | 0.000218696 | 0.04897876 |
| ENSCCRG00000021925 | -3.848744634 | 0.000220088 | 0.04897876 |
| ENSCCRG00000022038 | 4.109518742 | 0.000220571 | 0.04897876 |
| ENSCCRG00000033055 | 2.413739191 | 0.000221538 | 0.04897876 |
| ENSCCRG00000050549 | -4.732718749 | 0.000223413 | 0.04897876 |
| ENSCCRG00000041879 | -6.664210542 | 0.00022417 | 0.04897876 |
| ENSCCRG00000034281 | -5.87319707 | 0.000226127 | 0.049063192 |

## **Table S8.** The primer sequences synthesis for qRT-PCR.

| **Gene** | **ID** | **Forward** | **Reverse** |
| --- | --- | --- | --- |
| IL-6 | LOC109055884 | TCTCTTCCTGTCTGCCGTACTG | TCCCTCTCAAACTGCTGGTCTC |
| IL-8 | LOC109078242 | GGATGATTCCTGCTGGACCAAACTG | TGTTGGCTTGTGACGTTCTTCTGG |
| TNF-α | LOC109063472 | AGGTGATGGTGTCGAGGAGGAAG | AGACTTGTTGAGCGTGAAGCAGAC |
| IL-12b | LOC 109108834 | CAACAGTGGCAGAAGTGGTAGTG | TGTCAGGCATTGGCAGAGGTC |
| CXCL10 | LOC109084082 | CTGCTGTGTGCTCTGTGGTTAG | CTTTGCCTCCCTTTGCCCTTC |
| NDUFA2 | LOC122134707 | CGGCTGTGGTTCGTGGAATCG | ACATCAGTACGCTTAGGGCTTTGC |
| NDUFA3 | LOC109087693 | TTCAGCCAATCCAGAGCGAAAGC | AGCTTGGTTCATCATTCCGGTGTAC |
| NDUFA6 | LOC109079876 | GAGCCGCCGCCGTTAAAGTC | GGTCGTGTCCGTGGTAGAACTTG |
| SDHC | LOC109111213 | TGCGTTGGTGTTACCTGAGAGTTAC | ACAGCAGTGACAATGTAGCCAGAAC |
| UQCRC2 | LOC109049624 | CGGACCAGAGCCCACAGATAGG | CCTTCACACGCCAGCAGAGC |
| UQCRC8 | LOC109056753 | TTAAGGTTGCAGGCGGAAGTTGTAG | AGCTGAGCGATTTCACTGGTCATTC |
| COX6B1 | LOC109055457 | TCGCATCCTTTATCGCATCTCAGTC | TCCTCGTCTGGTTCTGGTTCGG |
| ATP6V1D | LOC109109844 | GCTGCTGGTGACTTCAGTGCTAC | TTCCTCTCGCTCCCGTTCGTC |
| DRP1 | LOC109087292 | ACAACAACATTGAGGAGCAGAGAC | GGCACACGCACATCAAGTAGG |
| MFN1 | LOC109072393 | TCACCTCTTCCTCTGCTCCCTTG | CCACAGAACGCCACACCATTCC |
| MFN2 | LOC109065032 | ACCAGTATGCCTCCTTTCCCTCAG | ATCACTCCGCCAACGACTATAATGC |
| cGAS | LOC109112034 | CCAACCCAAAGCCAAATCCAAAGC | TCCGCAGACTTATCCTCCCTGTC |
| STING | LOC109097113 | AGATGTCAGGTGTGATGGGAGAGG | AGTCCAGCAGCCAAGCAAACAG |
| TBK1 | LOC109074335 | TGGAGCAGTTACCGCAGAAGATTG | TCCTTCACGACTCCTTCCATCTCC |
| IRF3 | LOC109087794 | GTGTGGGTCATCAGGGTAGCAATC | ACCAACTGTTCCTTCACCATCTTCC |
| β-actin | LOC109073280 | CAGATGTGGATCAGCAAGCAGGAG | TTGAGAGGTTTGGATTGGTCGTTCG |

## **Table S9.** The information of used antibodies for western blot.

| **Antibodies** | **Dilution**  **ratio** | **KDa** | **Resource** | **Cat#** |
| --- | --- | --- | --- | --- |
| IL-6 | 1:2000 | 20 | Wanleibio | WL02841 |
| IL-8 | 1:2000 | 11 | ABclonal | A2541 |
| TNF-α | 1:500 | 26 | ABclonal | A23264 |
| IL-12 | 1:2000 | 42 | ABclonal | A25798 |
| CXCL10 | 1:2000 | 11 | ABclonal | A1457 |
| NLRP3 | 1:2000 | 110 | ABclonal | A5652 |
| CASP1 | 1:1000 | 48 | ABclonal | A20470 |
| Cleaved CASP1 (p20) | 1:1000 | 20 | Wanleibio | WL02996a |
| Full length+N terminal GSDMD | 1:1000 | 53/38 | ABclonal | A20197 |
| IL-1β | 1:1000 | 35 | ABclonal | A1112 |
| Cleaved IL-1β | 1:1000 | 17 | Invitrogen | PA5-105048 |
| HK2 | 1:2000 | 102 | ImmunoWay | YM8118 |
| PKM2 | 1:1000 | 60 | ImmunoWay | YT3777 |
| LDHA | 1:1500 | 37 | ABclonal | A1146 |
| PDK1 | 1:1500 | 49 | ABclonal | A0834 |
| G6PD | 1:1000 | 59 | ABclonal | A13983 |
| Pan Kla | 1:1000 | 30/23 | ABclonal | A23004 |
| H3K14la | 1:1000 | 17 | ABclonal | A18808 |
| H3K18la | 1:1000 | 17 | ABclonal | A18807 |
| Histone H3 | 1:1000 | 17 | ABclonal | A2348 |
| DRP1 | 1:1000 | 82 | ABclonal | A21968 |
| MFN1 | 1:1000 | 84 | ABclonal | A9880 |
| MFN2 | 1:500 | 80 | Wanleibio | WL06347 |
| cGAS | 1:500 | 62 | ABclonal | A8335 |
| STING | 1:1500 | 42 | ABclonal | A3575 |
| Phosphorylated STING | 1:1000 | 40 | ABclona | AP1369 |
| TBK1 | 1:2000 | 84 | ABclonal | A2573 |
| Phosphorylated TBK1 | 1:1000 | 84 | ABclona | AP1026 |
| IRF3 | 1:1000 | 47 | ABclonal | A2172 |
| Phosphorylated IRF3 | 1:1000 | 55 | ABclona | AP0995 |
| IFN-α1 | 1:500 | 18 | ABclonal | A0285 |
| IFN-β1 | 1:200 | 22 | Invitrogen | PA5-20390 |
| β-actin | 1:5000 | 42 | Bioss | BS-0061R |

# Supplementary Figures


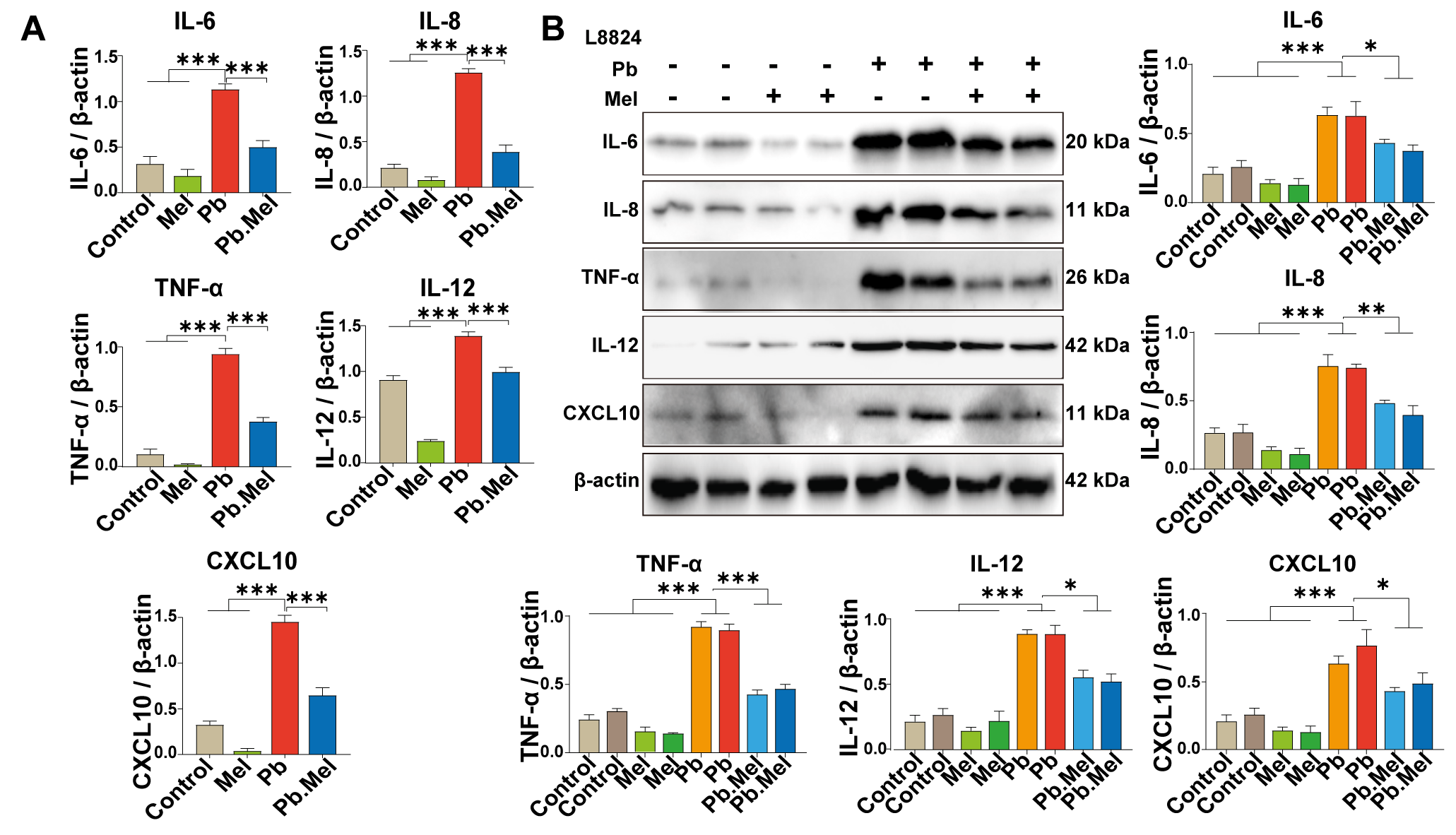


## Figure S1. Mel allieviates Pb-mediated inflammatory cytokines in protein manner. A) Statistical protein levels of inflammatory cytokines in carp liver. Each value represents the mean ± SD, **p* < 0.05, ** *p* < 0.01, and*** *p* < 0.001. B) The expression of inflammatory genes in the L8824 cell models.


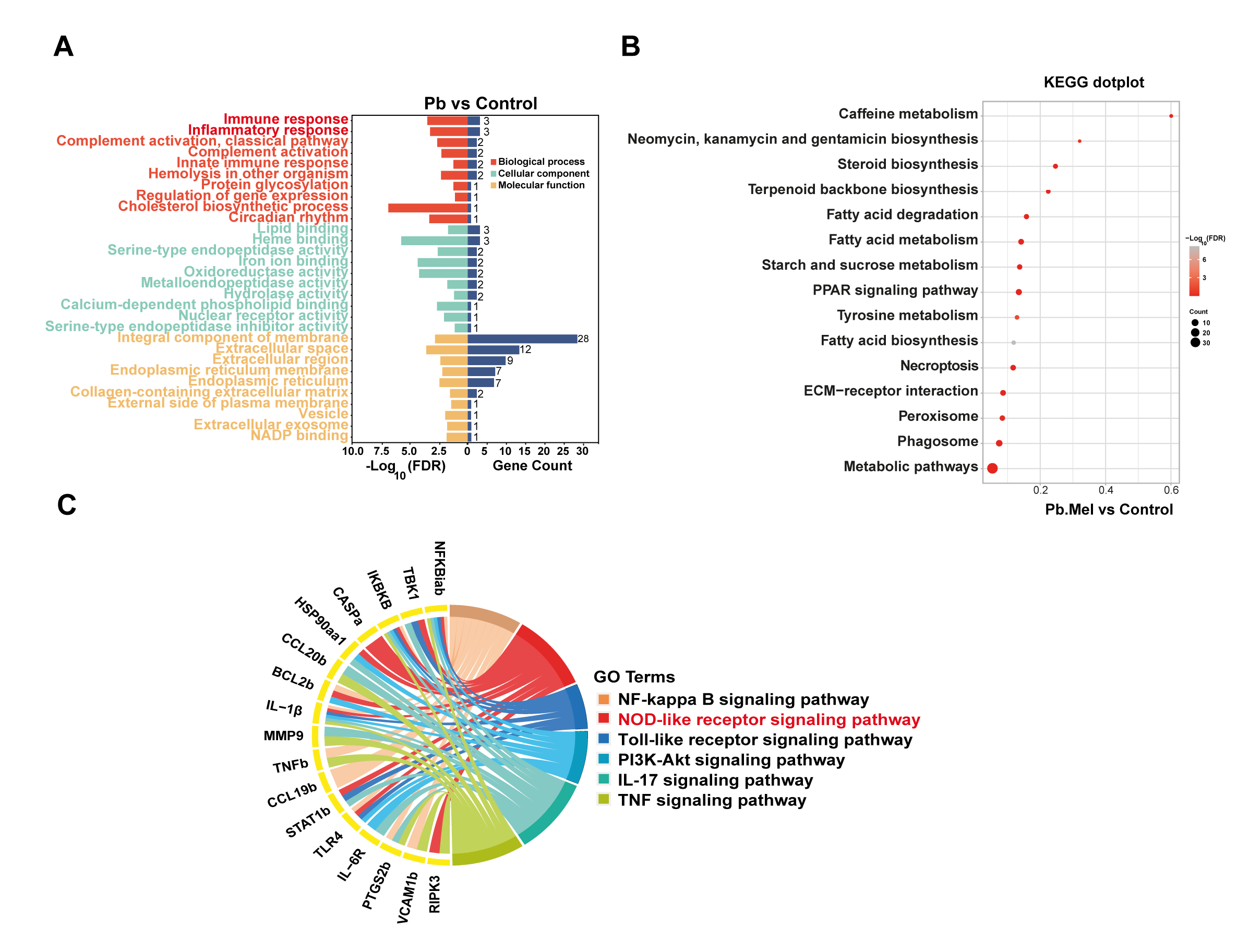


## Figure S2. Mel alleviates Pb-mediated pyroptosis. A) Go enrichment analysis results. B) KEGG enrichment analysis between the Pb.Mel and Pb group. C) The pathway analysis for inflammatory-related DEGs.


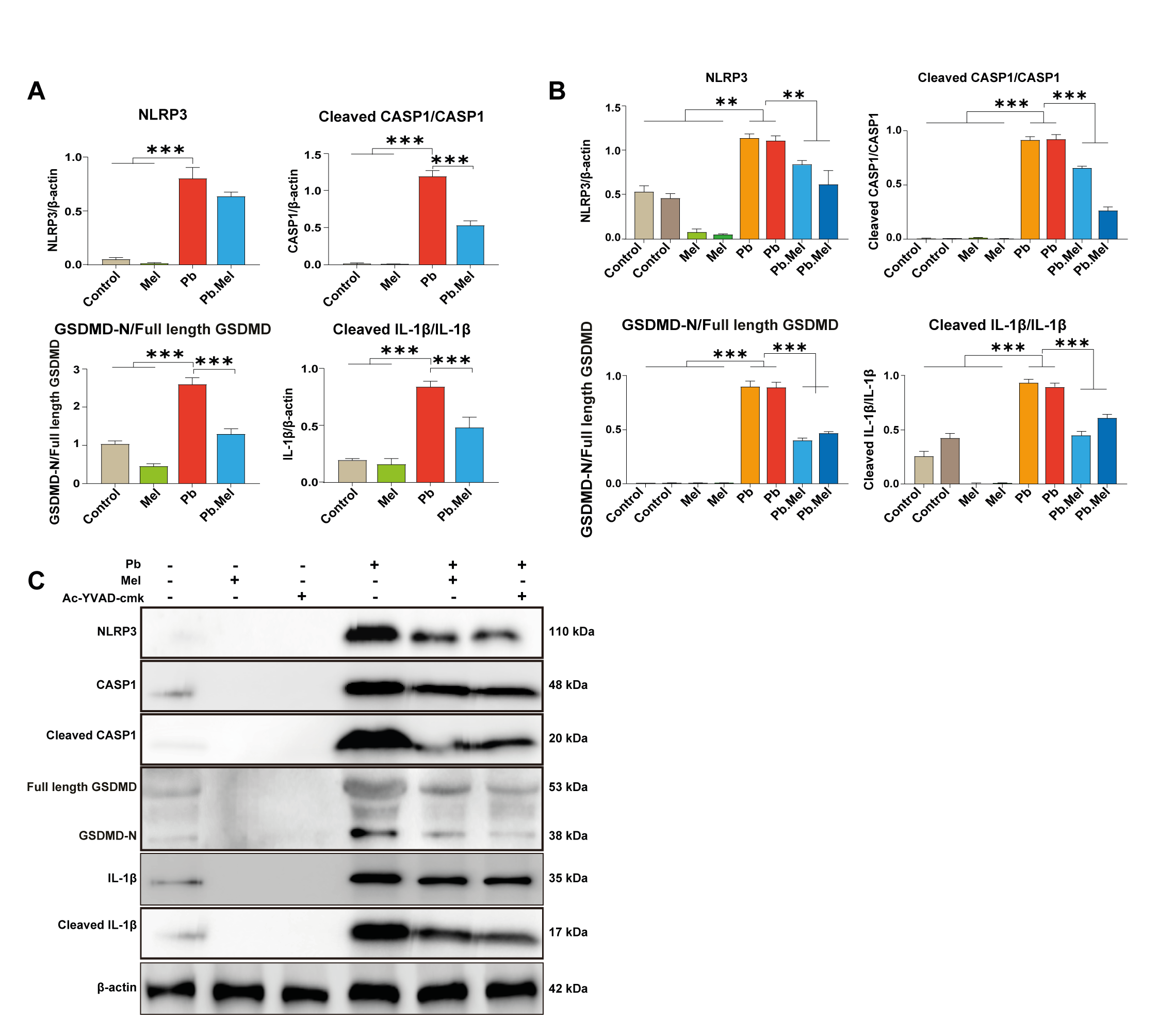


## Figure S3. Statistical protein levels of pyroptosis-related genes in carp liver and L8824 cell models.


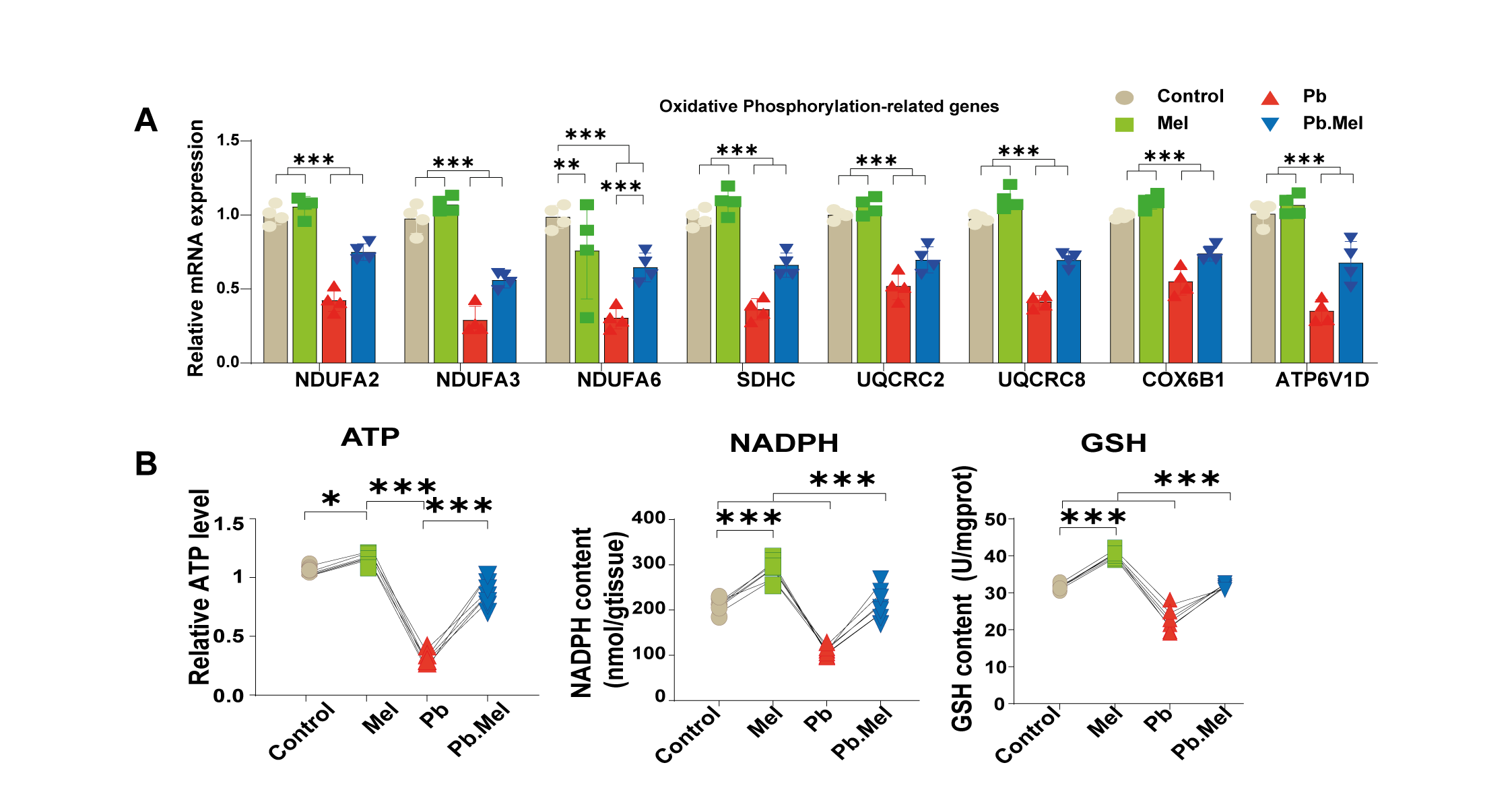


## Figure S4. Mel alleviates Pb-mediated glucose metabolism reprogramming. A) Relative mRNA expression of oxidative phosphorylation-related genes in carp liver (n=4). B) The ATP level and content of NADPH and GSH in carp liver.


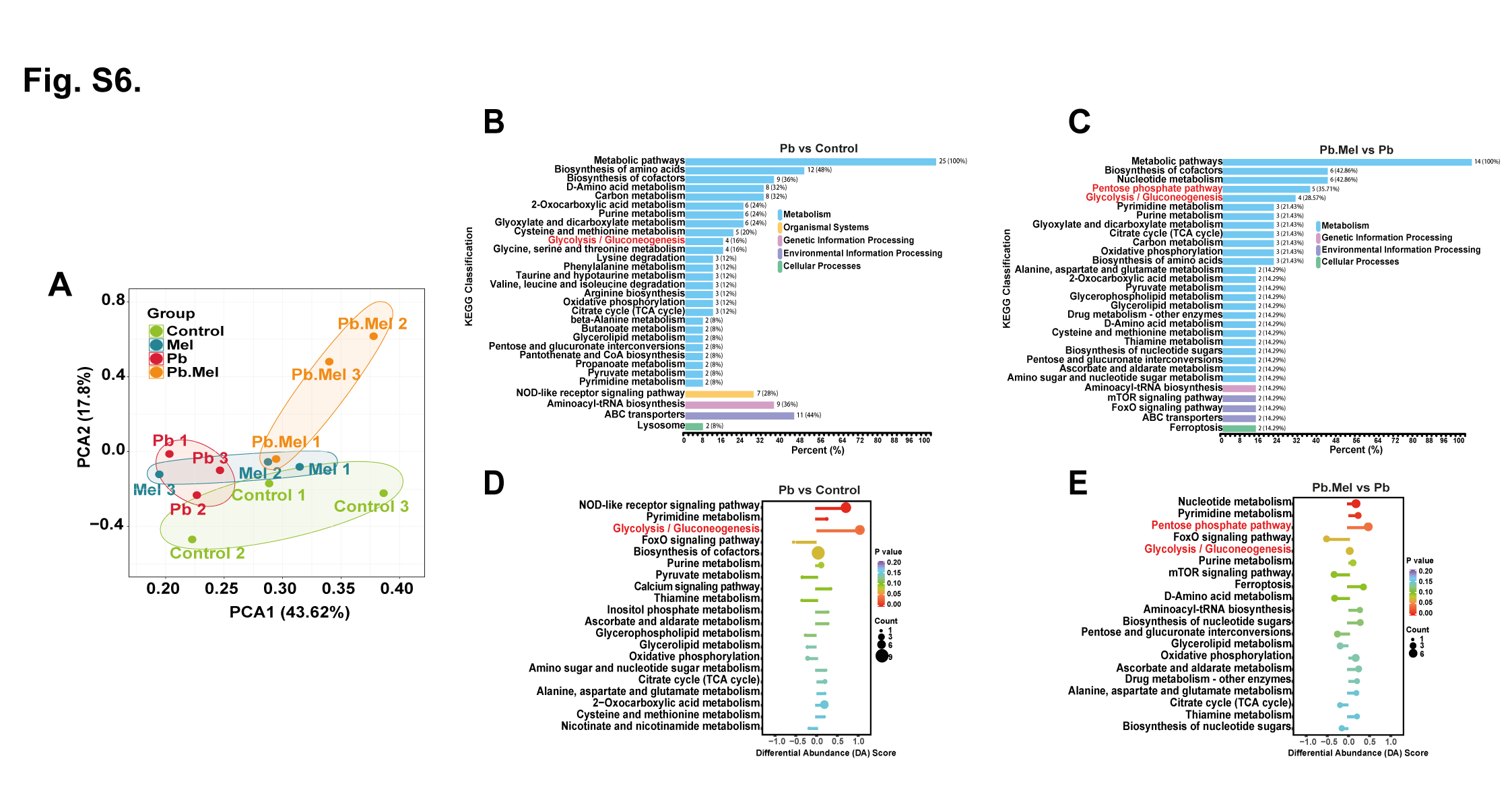


## Figure S5. Mel alleviates Pb-mediated glycolysis by promoting PPP. A) Principal component analysis of targeted metabolites in carp liver. B)-C) KEGG enrichment in targeted metabolomics of carp liver tissues. D)-E) Top 20 pathways were enriched between the Pb and Control group, and between the Pb.Mel and Pb group.


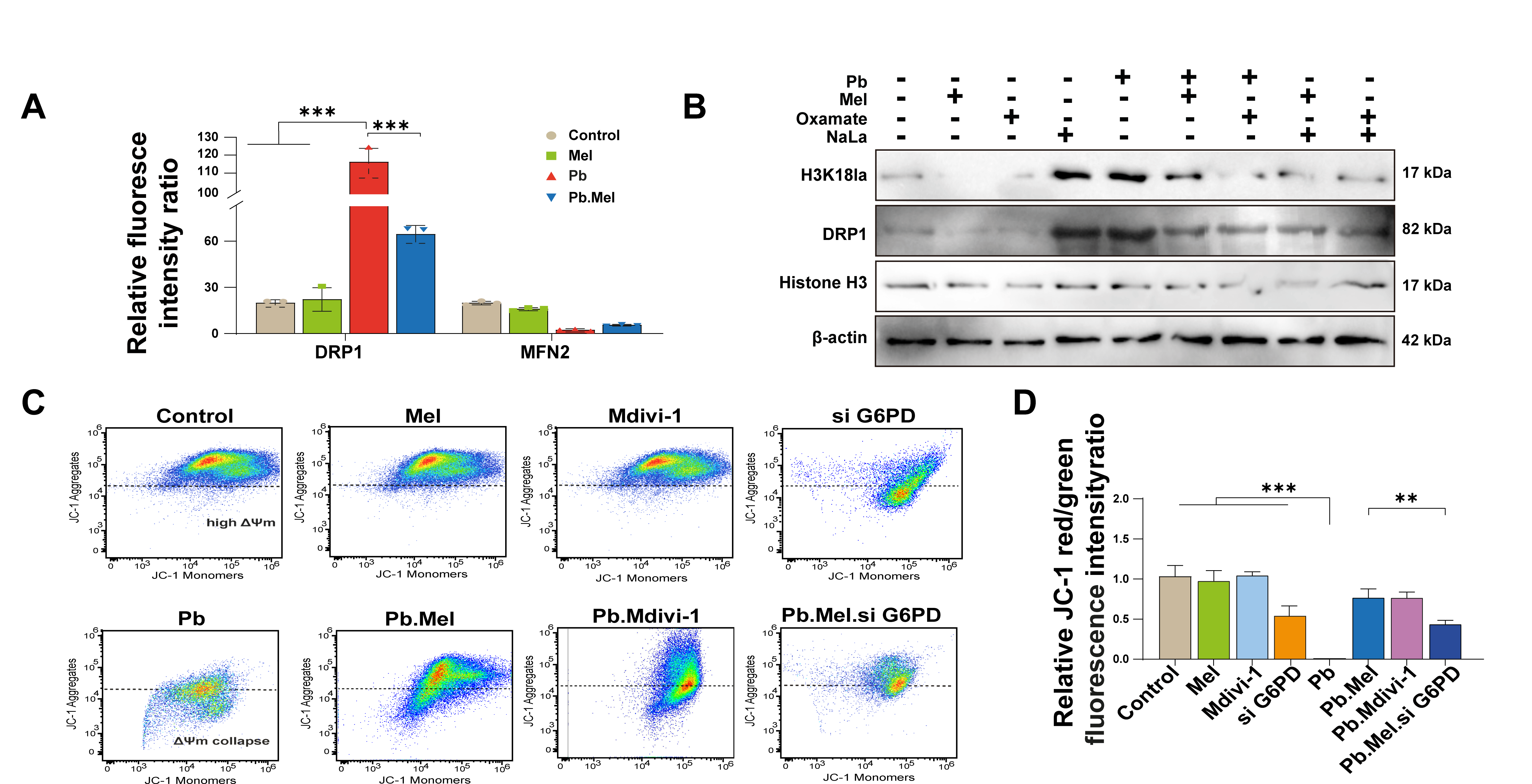


## Figure S6. Mel inhibits mitochondrial fission and alleviates Pb-triggered mtDNA leakage. A) Quantification of fluoresce intensity of DRP1 and MFN2 in liver tissue. B) The expression of DRP1 was positively correlated with H3K18la. C) The JC-1 staining assessed with flow cytometry in vitro. D) Quantification of JC-1 fluoresce intensity in cell models.


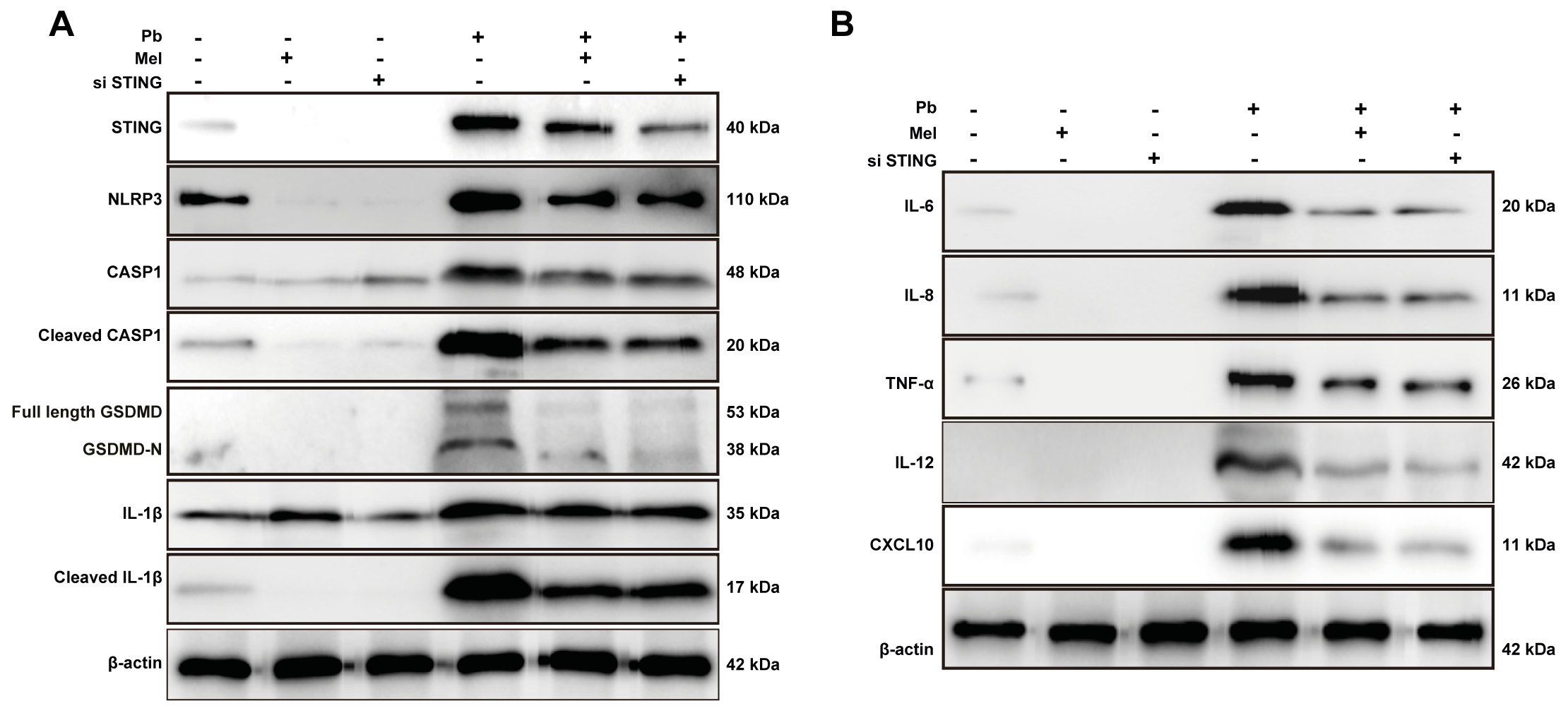


## Figure S7. Mel alleviates Pb-mediated glycolysis by promoting PPP. A) Principal component analysis of targeted metabolites in carp liver. B)-C) KEGG enrichment in targeted metabolomics of carp liver tissues. D)-E) Top 20 pathways were enriched between the Pb and Control group, and between the Pb.Mel and Pb group.


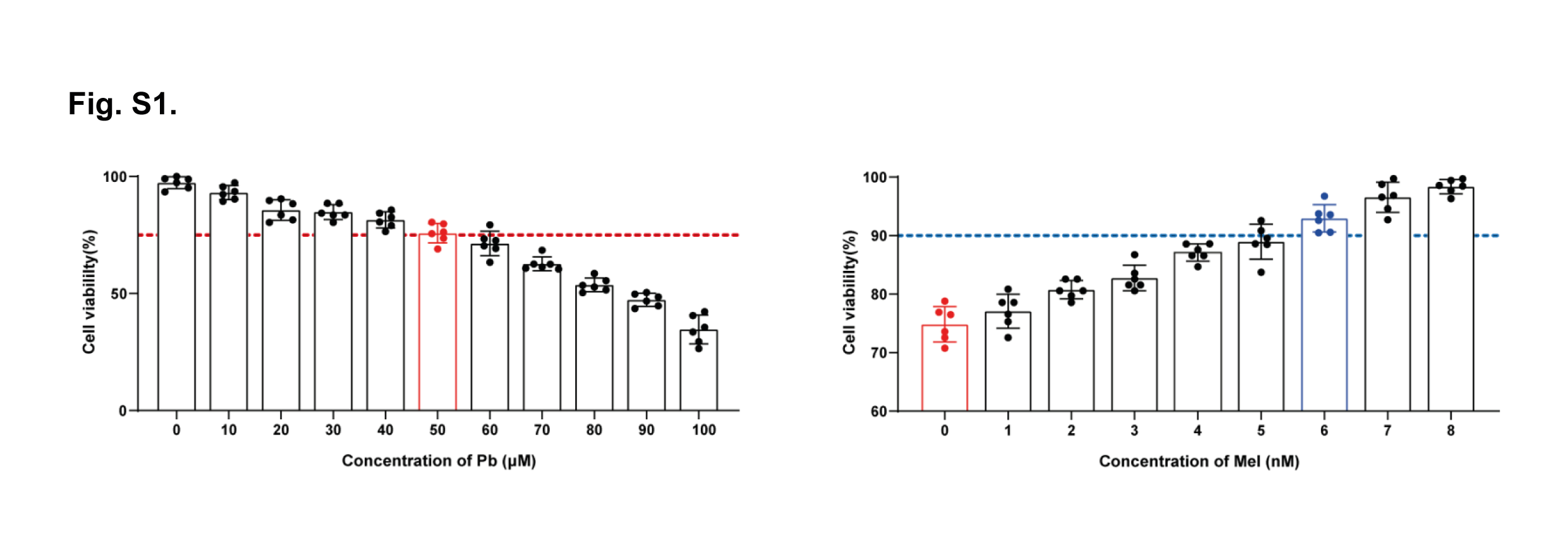


## Figure S8. Cell viability of L8824 cells under Pb and/or Mel treatment
